# Tracking Down the Evolution of Microorganisms by Exhaustive Bottom-Up Analysis of Proteomes

**DOI:** 10.3390/ijms27010109

**Published:** 2025-12-22

**Authors:** Dmitrii O. Kostenko, Natalya S. Bogatyreva, Alexey N. Fedorov

**Affiliations:** Federal State Institution, Federal Research Centre, Fundamentals of Biotechnology, Russian Academy of Sciences, Leninsky Prospect, 33, Build. 2, 119071 Moscow, Russia; dk0stenko@yandex.ru (D.O.K.); natali.bogatyreva@gmail.com (N.S.B.)

**Keywords:** k-mer frequencies, proteome, phylogenetic tree, hierarchical clustering

## Abstract

Proteomes are typically analyzed at the level of individual proteins or protein families. In this study, we introduce a bottom-up approach that treats proteomes as holistic entities by examining the properties of k-mers within entire proteomes and protein groups. We performed a comprehensive analysis of short amino acid k-mer (k = 1, 2, 3) distributions across all proteins in a given proteome. Using 86 bacterial proteomes representing 18 clades, we evaluated whether k-mer frequencies characterize uniquely the analyzed organisms. Remarkably, in a post hoc analysis, we found that the k-mer frequency vector unambiguously coevolves with the entire proteome—a pattern not observed even within specific protein groups, such as conserved ribosomal proteins or more variable nucleotide-binding proteins. This finding holds regardless of the k-mer calculation parameters or the distance metrics employed. Our results show that even a simple analysis based on tripeptide frequencies can precisely position proteomes within the k-mer space. Moreover, relationships derived from k-mer comparisons highly correlate with evolutionary relationships derived from phylogenetic trees, reaching up to 99% match with reference classification of the proteomes within major bacterial clades. These findings establish k-mer-based proteomic analysis as an additional robust and powerful feature for characterizing evolutionary relationships, opening new pathways in phylogenetics and evolutionary genomics.

## 1. Introduction

Reconstructing evolutionary history is one of the central challenges in evolutionary biology [1,2,3,4,5,6]. This task is crucial for several reasons, including: (i) understanding the evolutionary relationships among organisms and identifying their common ancestors, (ii) uncovering gene functions in comparative genomics, and (iii) studying the evolution and spread of pathogens during epidemics, such as COVID-19. Reconstructing evolutionary history involves detecting differences among organisms within a studied group, with the results typically represented as phylogenetic trees.

Alignment-free and compositional methods have emerged as powerful alternatives to traditional alignment-based approaches for large-scale proteome analysis. These methods represent sequences as numerical objects derived from short subsequences (k-mers), enabling rapid comparison of whole proteomes without the need for multiple sequence alignment. Within this framework, natural vector approaches encode each sequence as a fixed-length vector that summarizes k-mer counts together with positional statistics, preserving both composition and coarse spatial information along the sequence [7]. Other composition-vector methods, such as Feature Frequency Profiles (FFP), treat a genome or proteome as a “bag of k-mers” and compare organisms via distances between normalized k-mer frequency vectors, providing scalable alignment-free phylogenies at genome and proteome scales [8,9].

Recent developments have extended k-mer-based representations in several directions. Pipelines such as Snekmer combine amino-acid recoding with k-mer frequency vectors, grouping residues into reduced alphabets based on physicochemical properties to enhance sensitivity to remote homology while controlling dimensionality [10]. Chaos Game Representation (CGR) offers a complementary view by mapping sequences to fractal-like geometric patterns whose density reflects the frequencies of all k-mers simultaneously, allowing proteome signatures to be analyzed using image-based or multifractal descriptors [11]. Together, these natural vector, Snekmer-style, FFP, and CGR approaches illustrate the growing ecosystem of alignment-free, k-mer-based methods for proteome representation, each emphasizing different aspects of sequence composition, positional structure, and evolutionary signal [7,8,10,11].

The primary goal of our study was to analyze proteomes as unified entities using a “bottom-up” approach. We performed a rigorous, exhaustive statistical analysis of the distribution of all possible short amino acid fragments—single residues, dipeptides, and tripeptides—across every protein within a given proteome. Hereafter, these amino acid fragments are referred to as k-mers, with k values of 1, 2, or 3, respectively. This approach can be applied to solve such problems as evolutionary relationships analysis, protein function and translation efficiency prediction, defining relations between biochemical properties and k-mers composition and de novo determination of protein families [9,10,12,13,14,15,16,17,18,19]. We investigate here the potential of using k-mers in proteomes to reflect evolutionary relationships across organisms and, particularly, to accurately reconstruct phylogenetic trees.

For a dataset of 86 diverse bacterial proteomes spanning 18 distinct bacterial clades, we computed k-mer frequencies. After that, in a post hoc analysis, we demonstrate that amino acid 3-mer occurrences in proteomes are sufficient to reconstruct phylogenetic trees with remarkable accuracy—achieving up to 99% match with reference classification of the proteomes. This suggests that the evolution of microorganisms can be effectively traced through the evolution of short amino acid k-mers in their proteomes, whereas the whole proteomes represent operative evolutionary units.

## 2. Results

The aim of this study is to evaluate whether k-mer frequencies can effectively reflect interrelations between proteomes, exploring evolutionary relationships among organisms using proteomes as whole entities and thus be used to explore evolutionary relationships among organisms at the whole-proteome level. To achieve this, all proteins within the proteomes were segmented into overlapping k-mers, with *k* values of 1, 2, or 3, followed by an exhaustive analysis of the k-mers frequencies. Each proteome was represented as a $20^{k}$-dimensional vector, where each dimension corresponds to the frequency of a specific k-mer occurrence within that proteome. Intuitively, proteomes with similar k-mers distributions are expected to be positioned close to each other in this high-dimensional space, such that increasing dissimilarity in k-mer occurrence patterns corresponds to greater distance between proteomes.

For this analysis, we selected 86 bacterial proteomes representing 18 distinct clades to perform unsupervised clustering in the $20^{k}$-dimensional space (see Section 4). We aimed to resolve their relative positions and subsequently compare this spatial organization with an established phylogenetic tree derived from genome taxonomy databases. The established phylogenetic tree served as a gold standard in our research: bacteria belonging to the same clade were expected to locate close to each other in the k-mer space. Methodologically, the particular aim of this study was to determine the optimal proteome representation k-mer space, define appropriate distance metrics within this space, and explore how concordance between proteome k-mer-based clustering and evolutionary relationships inferred from conventional phylogenetics.

### 2.1. Hierarchical Clustering and Construction of Phylogenetic Tree

We evaluated hierarchical unsupervised clustering of the 86 proteomes using the Agglomerative Clustering method [20,21], testing all combinations of the following parameters presented in Section 4:

- *k* ∈ {1, 2, 3};
- Distance metrics *L*1, *L*2, *corr*(*p*, *q*), and *inter_a_*(*p*, *q*) for *a* ∈ {0.1, 0.05};
- Protein groups: whole proteome, membrane, non-membrane, nucleotide-binding, non-nucleotide-binding, ribosomal;
- Amino acid alphabet of 20 symbols or its reduction to 5;
- “Single”, “average” and “complete” clustering linkage parameter values.

**Figure 1:**
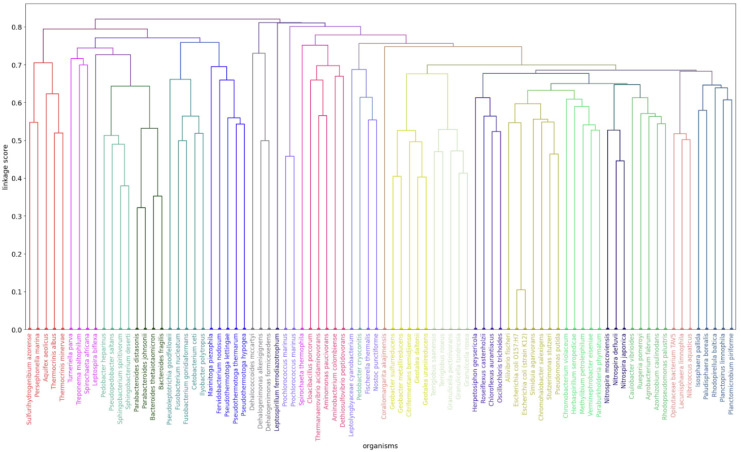
Binary tree obtained by means of hierarchical clustering. The Binary tree was constructed by Agglomerative Clustering based on 3-mers frequencies for ribosomal proteins on a 20-residue alphabet with *L1* metric and average linkage. The obtained clustering is represented by the structure of the binary tree while organisms belonging to the same clade are marked in one color for comparison. Close groups of leaves are expected to have the same color without other color leaves inclusion. Rand = 0.9929.

We then built the binary trees using the distances between proteomes in k-mer space as described in Section 4. (Distance matrices can be found in the “dist_matrices” folder within the Supplementary File S1). We visually analyzed all the obtained binary trees by comparing them with the known clade membership of the proteomes. The aim was to find the best values separately for each parameter. The closest to reference classification dendrograms were obtained with *k* = 3, while with *k* = 1 and *k* = 2 the clustering quality worsens. In turn, “average” linkage showed itself to be the best for estimating the distances between clusters on average for our task. The clustering quality significantly improves if we consider only ribosomal proteins of proteomes. Narrowing the alphabet to 5 symbols (see Section 4) significantly worsens the clustering results, so from the two considered variants it is much better to use the original alphabet of 20 amino acids. Among all the considered measures of distance between proteomes, the *L*1 metric showed good results a bit more often than other metrics. Using the combination of all the above parameters, we obtained the clustering depicted in Figure 1.

Here, reference classification was used only to colorize leaves (proteomes) of the dendrogram. Note that the clustering itself was performed in an unsupervised manner. All the constructed dendrograms are included in the “dendrograms” folder within Supplementary File S1. To verify results of the visual analysis, we applied the Rand index reflecting the degree of similarity between the estimated clustering and the reference classification (see Section 4). Our choice of the best individual parameters values was confirmed by averaging Rand values with fixing every single parameter value and varying other parameters. Mean and standard deviation of the obtained Rand index values are presented in Table 1.

It turned out that additionally, clustering with sufficient Rand index can be achieved using other parameter combinations. The five best combinations based on the Rand index are presented in Table 2 (full data can be obtained from “clustering_scores/aggl_clust_score.json” file within Supplementary File S1).

As evident from Table 2, the combination of parameters corresponding to the clustering in Figure 1 is indeed one of the best (it ranks second). At the same time, there are other combinations that rank among the top five in terms of Rand index. However, when making point changes to individual parameters, the previously considered combination is the most stable because all the other parameters keep their best values according to Table 1. Therefore, we will use this combination as our default. In accordance with Table 2, the choice of *k*, Alphabet, Protein group and Linkage values is clear and unambiguous, whereas the metric selection is less obvious, but taking into account Table 1, we assume that *L1* is the most appropriate metric. The proteome representation parameters should not be considered as parameters of the applied clustering method. We propose them as the way to establish k-mer space for bacterial proteomes which best represents evolutionary distances between them.

**Figure 2:**
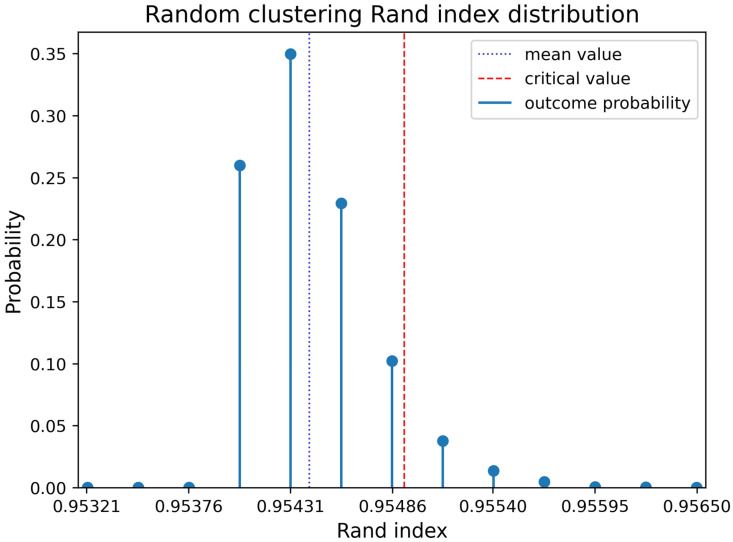
Random Agglomerative Clustering Rand index distribution.

To verify the statistical significance of our results, we generated 10,000 completely random binary trees. The mean value of the Rand index turned out to be 0.9544 with a standard deviation of 0.0003 (blue dotted line in Figure 2). A discrete distribution of the Rand index values is shown in Figure 2.

By applying *distfit* Python package (version 1.8.9) we found out that such a distribution is best approximated as a shifted and scaled Student’s t-distribution with 8.14 degrees of freedom. Let us consider a statistical hypothesis about the randomness of the Rand index value with a right-sided critical region. Let us assume that Rand index values of random clusterizations follow Student’s t-distribution. Then a Rand index value can be considered statistically significant with a significance level of 0.05 if it exceeds 0.9549 (red dotted line in Figure 2). Thus, all the previously considered clustering results can be declared statistically significant.

Such relatively high values of Rand index for random phylogenetic trees are related to an unlimited number of clusters which can be created in the process of transforming hierarchical clustering into non-hierarchical form by means of the algorithm presented in Section 4.4. The algorithm uses reference classification in order to match tree nodes to clusters in a reasonable way. There is a possibility to create a new clustering label for every leaf which does not belong to the same class as its sibling leaf in a random hierarchical clustering. At the same time proteomes which belong to the same class and randomly become siblings in such hierarchical clustering always get the same clustering label. Thus, it can be stated that transformation to non-hierarchical form maximizes Rand index for a given tree so even for a random tree it is usually quite high. That is why the statistical test was necessary for correct understanding of the results represented in Table 1 and Table 2.

### 2.2. Non-Hierarchical Clustering

**Figure 3:**
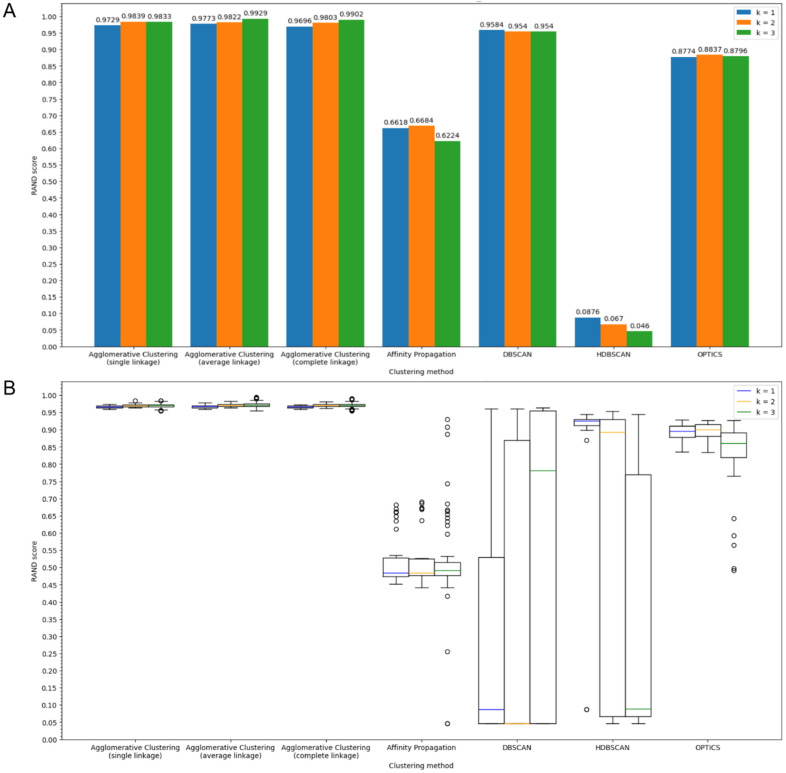
Dependence of the Rand index on the clustering method: (**A**) A histogram showing dependence of the Rand index on the clustering method using default parameters (20 symbols alphabet, ribosomal proteins group, *L1* distance function); (**B**) Boxplots for different clustering methods showing Rand index values distribution for various Alphabet, Protein group and Metric parameter values (statistical outliers are shown as circles).

We clustered the proteomes from the sample using non-hierarchical clustering methods, specifically Affinity Propagation, DBSCAN, OPTICS, and HDBSCAN using a set of clustering parameters. The quality of clustering was assessed using the Rand index. Then we compared these results with those obtained using Agglomerative Clustering. We constructed Rand index histograms for all metrics in case of *k* = 3 and metrics *corr*, *L*2, and *L*1 for *k* = 1 and *k* = 2 across all previously considered protein groups. Below is a Rand index histogram (Figure 3A) for ribosomal proteins using the *L1* metric as an example and a box plot generated using Rand values from different parameter (Alphabet, Protein group and Metric values) combinations to visualize the overall trend.

It can be concluded that the highest Rand index values are consistently observed in clustering results obtained using Agglomerative Clustering. This trend is also evident for other protein groups and when using different metrics. However, superiority of Agglomerative Clustering may be related partly to the algorithm for transforming hierarchical clustering into non-hierarchical form, which uses reference classification to best match tree nodes to clusters (see Section 4). Thus, Agglomerative Clustering indeed allows one to get the best clustering compared to other methods, but it does not guarantee that it will be obtained if the tree nodes and clusters are incorrectly matched.

As evident from Figure 3B, the clustering values derived from methods other than Agglomerative clustering exhibit much less predictability: altering a single parameter can cause the Rand index to fluctuate dramatically, ranging from near 0 to near 1 and vice versa. In contrast to Agglomerative Clustering, where *k* = 3 almost invariably yields the best results, such a consistent pattern is not observed with other methods. All histograms and raw data are included in the “clustering_scores” folder within Supplementary File S1.

### 2.3. The Set of Most Abundant k-Mers

In this section, we determine the extent to which sets of the most common k-mers in different bacteria overlap with each other. We focused on k-mers with a length of 3 (*k* = 3). For each bacterium in our sample, we selected those k-mers that fell within the top *I_t_%* most common in their proteomes, and considered them common if they were present in at least *I_c_%* of the proteomes from the sample. The values for *I_t_* were set at 5%, 10%, and 15%, while *I_c_* was set at 90% and 100%. In this analysis, we not only examined entire proteomes but also checked individual groups of proteins (see Section 4). The results obtained for *I_t_*% = 10% and *I_c_*% = 90% are presented in Table 3.

The table shows that membrane proteins have the largest number of common most abundant k-mers, whereas ribosomal proteins have the fewest. This pattern persists across different *I_t_* and *I_c_* values. All relevant data and tables (including specific k-mers) are provided in “intersection” and “inter_tables” folders within Supplementary File S1.

### 2.4. Clustering of Protein Groups

In the previous sections, we investigated the possibility of clustering proteomes based on their k-mer statistics in a manner that preserves taxonomic relationships between them. However, equally interesting is the possibility of clustering proteins within one proteome to identify groups of (non-)membrane proteins, (non-)nucleotide-binding proteins, and (non-)ribosomal proteins. In particular, based on Table 3, one can conclude that membrane proteins are similar to each other in different bacteria and, therefore, it might be possible to identify a group of them inside one bacterium by clustering using k-mer statistics. We attempted to cluster proteins from the *E. coli* proteome using previously mentioned non-hierarchical clustering methods as well as the *k*-means method [22], but we observed no separation of proteins by their properties into distinct clusters; instead, the results appeared to be random. Therefore, we decided to test directly the feasibility of dividing these proteins into such groups based on their k-mer statistics.

To investigate the possibility for protein clustering within a proteome, we calculated within-group distances *D_i_* (1) and between-group distances *D_ij_* (2) as it is described in Section 4. We did this separately for 18 bacteria (one representative for each clade in the sample), the UniProt organism identifier is given in paranthesis: *Aquifex aeolicus* (UP000000798), *Azorhizobium caulinodans* (UP000000270), *Coraliomargarita akajimensis* (UP000000925), *Chloroflexus aurantiacus* (UP000002008), *Cetobacterium ceti* (UP000191153), *Dehalococcoides mccartyi* (UP000002506), *Escherichia coli* (UP000000625), *Fervidobacterium nodosum* (UP000002415), *Geobacter sulfurreducens* (UP000000577), *Granulicella tundricola* (UP000000343), *Herbaspirillum seropedicae* (UP000000329), *Nitrospira defluvii* (UP000001660), *Parabacteroides distasonis* (UP000000566), *Planctopirus limnophila* (UP000002220). Since the results were practically the same for these 18 bacteria, we averaged the within-group and inter-group distances (data for all organisms are given in JSON format in “protein_groups_dist” folder within Supplementary File S1). The distances for the *L1* metric and *k* = 3—our optimal and recommended parameters—are presented in Table 4 and Table 5. Table 4 displays within-group distances, while Table 5 provides between-group distances obtained with these parameters.

It can be observed that for membrane proteins, the average within-group distance is the smallest, and for ribosomal proteins, it is the largest. This is consistent with the results presented in Table 3 and indicates that in terms of k-mer statistics, membrane proteins are generally more similar to each other than proteins constituting other protein groups. In contrast, ribosomal proteins differ significantly from one another.

As can be seen from Table 5, the between-group distances do not exceed the within-group distances, and therefore, it is impossible to separate unambiguously protein groups without overlapping when clustering by 3-mer composition. For *k* = 2 and *k* = 1 the character of the picture remains largely unchanged (see “protein_groups_dist” folder within Supplementary File S1) while, as it was shown previously, proteomes clustering quality depends considerably on *k* value. It is also clear from Table 5 that ribosomal proteins differ greatly in their k-mer composition not only from each other but also from proteins in other groups.

For comparison, we calculated both within-group and between-group distances between proteomes. For a group of ribosomal proteins using *k* = 3 and *L1* metric, the within-group distances across all bacterial clades represented in our sample was found to be 0.603, on average (*m*_1_), with a standard deviation of 0.072 (*σ*_1_). The corresponding values for between-group distances were *m*_2_ = 0.779, on average, with a standard deviation: *σ*_2_ = 0.063. Thus, even if we move one standard deviation from mean values towards each other (i.e., reduce between-group distance by its standard deviation and increase within-group distance by its standard deviation), between-group distances will still exceed within-group distances: (*m*_2_ − *σ*_2_) − (*m*_1_ *+ σ*_1_) = (0.779 − 0.063) − (0.603 *+* 0.072) = 0.041 > 0. For most other parameter combinations, between-group distances are significantly larger than within-group distances as well. All data regarding within-group and between-group distances for proteomes are given in “proteomes_groups_dist” folder within Supplementary File S1.

## 3. Discussion

To elucidate the evolutionary relationships among organisms and reconstruct their evolutionary history, it is essential to identify differences among them. These differences supposedly should correlate with evolutionary distance; that is, the greater the evolutionary divergence between organisms, the more pronounced the differences should be. Traditionally, multiple sequence alignments of genomic and proteomic sequences have been employed to reconstruct evolutionary histories. For both types of sequences, such alignments facilitate the tracing of accumulated changes—substitutions, insertions, and deletions—at specific positions within sequences. Also, alignment-free methods are applied for phylogenetic analysis and taxonomic classification. Most of these methods are based on DNA, RNA or protein k-mers exact matching or statistics [9,12,13,14,19]. While the traditional approach focuses on specific genes or individual proteins, our aim here is to explore the whole proteome as an “evolutionary unit”.

To this goal, we explored the feasibility of utilizing an integral parameter that characterizes the proteome or its subsets (protein groups), specifically the occurrence of short fragments of length *k* (k-mers), as a descriptor of inter-organismal differences. As a benchmark for success and proof of concept, we focused on constructing binary trees and assessing their correspondence with the actual phylogenetic tree of 86 bacterial species across 18 clades. Our findings indicate that for *k* = 1 and *k* = 2, the statistics of k-mer occurrences often proved less sufficient (but still statistically significant) for reliably positioning organisms on the phylogenetic tree relative to one another [23]. However, for *k* = 3, we achieved an accuracy of up to 0.9937.

Clearly, there is an upper limit for statistically reliable analysis depending on the size of the proteome. For example, the *E. coli* proteome contains approximately 1,350,000 amino acids (and a similar number of k-mers). For k = 4, there are 160,000 possible k-mer types, which corresponds to an average of 8.44 occurrences per type. However, because proteins are not random heteropolymers, k-mers are distributed very unevenly across k-mers: even for k = 3, some k-mers are absent, and for k ≥ 4 the k-mer vectors become increasingly sparse. This sparsity impairs the performance of many mathematical and machine-learning methods. For this reason, we believe, for bacterial proteomes the top *k* value is 3. Also, the approach can be applied to particular groups of proteins, e.g., ribosomal, soluble cytoplasmic or membrane ones. In addition, increasing k substantially raises both the computation time and the memory requirements for working with such representations.

Our analysis revealed that utilizing only a group of ribosomal proteins in conjunction with the *L1* metric (also known as Manhattan or taxicab distance) and complete connectivity in graph calculations yielded stable results. We employed the Agglomerative Clustering method; alternative clustering approaches produced unstable outcomes. Notably, we utilized the original alphabet of 20 amino acids; attempts to reduce this alphabet to five symbols (as detailed in Section 4) significantly degraded our results.

The high-quality clustering observed can be attributed to the fact that, for the chosen metric, the average distance between ribosomal proteins from proteomes within a single clade is significantly less than that between proteomes from different clades. It is conceivable that as the number of analyzed organisms increases, the k-mer space may become more densely populated, necessitating an exploration of higher values of *k*. However, we did not investigate higher *k*-values in this study due to the substantial increase in computational cost, as our aim was to perform a fast yet accurate analysis of our sample involving 86 bacterial species.

Interestingly, ribosomal proteins exhibited the highest potential for differentiating proteomes despite the ribosome machinery being among the most conserved elements of genomic architecture [24,25,26]. This conservation is why ribosomal 16S rRNA sequences are commonly employed to align evolutionarily distant organisms; these sequences exhibit minimal evolutionary change. It is reasonable to anticipate that ribosomal proteins would similarly display high conservation. However, our analysis revealed that ribosomal proteins surprisingly exhibited significant diversity in k-mer statistics: only 56 out of 800 (7%) of the most abundant 3-mers were common across at least 90% of the studied bacteria (see Table 3). In contrast, membrane proteins showed a much higher overlap rate, with 261 out of 800 (32.6%) being observed.

**Figure 4:**
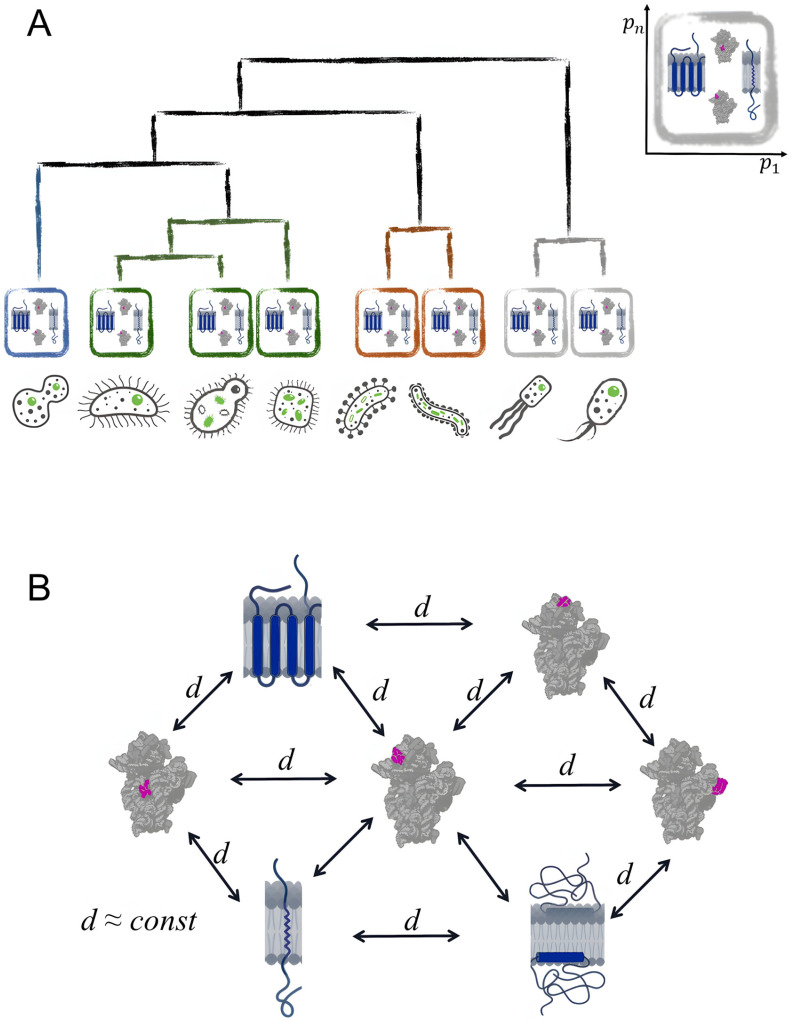
Proteomes and proteins clustering: (**A**) Proteomes can be arranged in a close to the reference phylogenetic tree considering only k-mers frequencies; (**B**) Proteins of an arbitrary proteome are not separable into such groups as (non-)membrane proteins, (non-)nucleotide-binding proteins, and ribosomal proteins by means of k-mers frequencies analysis; distances between vectors representing proteins form different groups are approximately equal to distances between vectors representing proteins within one group.

It is noteworthy that despite ribosomal proteins’ demonstrated capacity to distinguish proteomes within k-mer space—unlike other protein groups—the k-mer statistics alone were insufficient to differentiate ribosomal proteins from membrane proteins or any other distinct protein groups within the same organism. It can be related to the sparsity of vectors representing separate proteins. The difficulties of arranging proteins within a proteome by means of k-mer frequencies analysis compared to proteomes clustering capabilities are illustrated with Figure 4.

From a technical point of view, our findings enable accurate and statistically significant clustering of proteomes based on their k-mer composition. The results underscore that amino acid k-mer statistics and their corresponding placement within k-mer space provide a powerful and interpretable framework for uncovering evolutionary relationships among organisms.

From a perspective of primordial evolution, the obtained results allow us to draw the following conclusions:

The distribution of k-mers in proteomes of different evolutionary diverse bacteria could help us to find out the most prevalent amino acid stretches among the majority if not all species with expectation to find out preferable “building blocks” for successfully folding units in primordial polypeptide chains. This follows from the fact the first and essential pattern shared by all proteins is their ability to fold into a particular unique tertiary structure. One of the apparent problems of protein evolution is that given an obvious enormous variety of protein sequences, $20^{n}$ for each considered protein length *n*, it was impossible in the evolution to try even a tiny fractions of all possible polypeptide combinations to fold into particular stable conformation and carry out functionality essential for maintaining cell viability. Hence the idea could be to find some conservative subset of short peptide sequences which might have been used as blocks in polypeptide chains allowing them to rather successfully fold into stable structures and then to become a subject of further evolution for proper functionality, stability, etc. Using peptide blocks instead of full variability of single amino acids in a polypeptide chain would have critically reduced enormous variability of protein structures and greatly improve chances for folding into at least somewhat stable conformation.

The obtained results show that, generally speaking, there is no substantial set of common prevalent short peptides (k-mers) among if not all but a majority of the tested bacterial proteomes. However, such peptides are present among membrane proteins, i.e., proteins folding and functioning in the hydrophobic phase. This line of work requires further in- depth analysis of much larger proteome sets, including eukaryotic organisms.

## 4. Materials and Methods

### 4.1. Representation of Proteins and Proteomes

In this paper, we represent proteins as vectors *p* = (*p*_1_, *p*_2_, …, *p_n_*), where *n* is the size (i.e., the cardinality) of the set of all k-mers defined for the given *k* for the amino acid alphabet *A*. Thus, *n* = *|A|^k^* and *|A|* = 20 for proteins; $p_{i}=\frac{c_{i}}{\sum_{j=1}^{n}c_{j}}$, where *c_i_* is the number of *k*-mers of *i*-th type in the given protein. A proteome is represented by a vector of the same dimensionality *n*: *P* = (*P*_1_, *P*_2_, …, *P_n_*), where $P_{i}=\frac{\sum_{j\;=\;1}^{n}c_{ij}}{\sum_{i\;=\;1}^{n}\sum_{j\;=\;1}^{m}c_{ij}}$, and *c_ij_* is the number of k-mers of *i*-th type in *j*-th protein in the given proteome, *m* being the number of proteins in the proteome.

We considered several metrics of the distance between proteins and proteomes. Let *q* and *p* be two proteins or two proteomes under consideration. Then a general formula for distance is defined as $d(p,q)=\sqrt[{a}]{\sum_{i\;=\;1}^{n}{\left|p_{i}-q_{i}\right|}^{b}}$, where *a* and *b* are parameters. For *a* = *b* = 1 we get the *L*1 (Manhattan, taxicab) distance, while the case of *a* = *b* = 2 represents *L*2 (Euclidean) distance. We also used correlation distance $corr(p,q)=\frac{1-r(p,q)}{2}$, where *r*(*p*, *q*) is the Pearson correlation coefficient estimate of random variables *p* and *q* represented by samples of k-mer probability values (both samples include *n* measurements where *n* = *|A|^k^*). Additionally, we considered distance ${inter}_{a}(p,q)=\frac{\left|P_{a}\cap Q_{a}\right|}{a\times n}$, where *P_a_* and *Q_a_* are subsets of *a* × *n* of the most frequent k-mers in *p* and *q*, respectively; here *a* ∈ (0, 1) is a coefficient responsible for the number of k-mers taken into account (we used *a* ∈ {0.1, 0.05}).

### 4.2. Proteome Dataset and Its Processing

**Figure 5:**
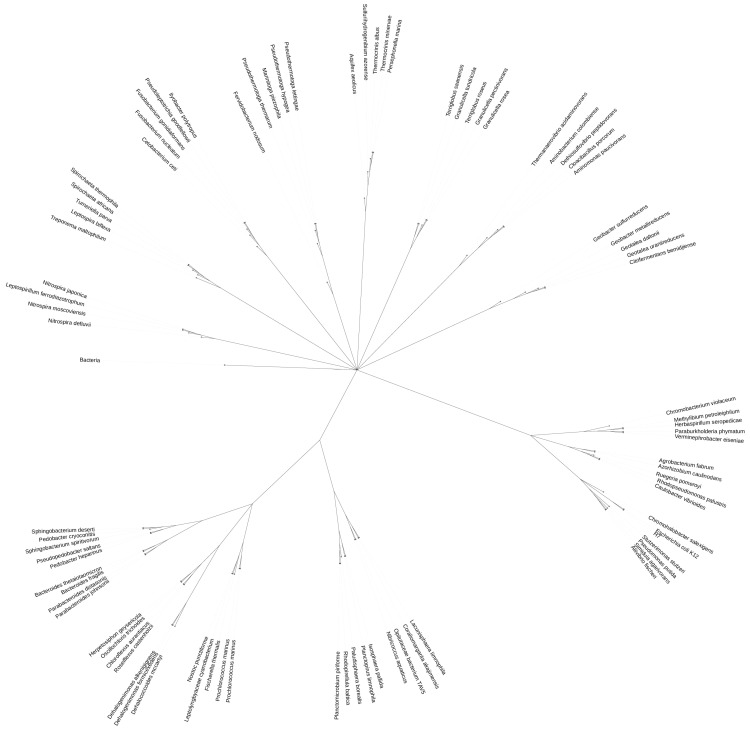
Sample. For this study, 86 bacterial proteomes were obtained. The sample included 3–6 representatives from each of 18 different bacterial clades: *gammaproteobacteria*, *betaproteobacteria*, *alphaproteobacteria*, *spirochaetia*, *fusobacteriia*, *cyanophyceae*, *terriglobia*, *sphingobacteriia*, *dehalococcoidia*, *chloroflexia*, *thermotogae*, *desulfuromonadia*, *bacteroidia*, *synergistia*, *aquificae*, *planctomycetia*, *nitrospira*, *opitutae*.

Bacterial proteomes for this study were obtained from the UniProt database. We selected 86 bacterial proteomes based on the following criteria: a low number of gaps and duplicates according to BUSCO assessment [27], and “standard” or “close to standard” values as evaluated by the CPD assessment on the UniProt website. The sample included 3–6 representatives from each of 18 different bacterial clades: *gammaproteobacteria*, *betaproteobacteria*, *alphaproteobacteria*, *spirochaetia*, *fusobacteriia*, *cyanophyceae*, *terriglobia*, *sphingobacteriia*, *dehalococcoidia*, *chloroflexia*, *thermotogae*, *desulfuromonadia*, *bacteroidia*, *synergistia*, *aquificae*, *planctomycetia*, *nitrospira*, *opitutae*. A corresponding phylogenetic tree (Figure 5) was obtained by means of iTOL [28]. The clades were manually selected to ensure uniform coverage of the bacterial phylogenetic trees [29,30]. A file “sample_data.json” containing the proteome identifiers included in the sample is provided in the Supplementary File S1.

In most calculations, we examined not only entire proteomes but also the following subsets (protein groups) of proteomes: membrane proteins, nucleotide-binding proteins, and ribosomal proteins. Additionally, we considered the following complementary subsets within the proteome: non-membrane and non-nucleotide-binding proteins. We did not analyze the non-ribosomal protein group separately in this study due to the limited number of ribosomal proteins. As a result, including them would not significantly impact the overall statistics.

We also explored reducing the 20-amino acid alphabet to 5 classes:

- Aliphatic: Alanine, Isoleucine, Leucine, Methionine, Proline, Valine.
- Aromatic: Phenylalanine, Tryptophan, Tyrosine.
- Uncharged: Cysteine, Glycine, Asparagine, Glutamine, Serine, Threonine.
- Positively charged: Histidine, Lysine, Arginine.
- Negatively charged: Aspartic acid, Glutamic acid.

While reducing the alphabet results in some loss of information, it also decreases the dimensionality of the vectors representing proteins and proteomes (*n* = 5*^k^* instead of *n* = 20*^k^*). In contexts where the occurrence of a specific k-mer is treated as the realization of a random variable, this reduction allows us to obtain a less sparse vector. We did not consider k-mers containing amino acids pyrrolysine and selenocysteine.

### 4.3. Clustering Algorithms

**Figure 6:**
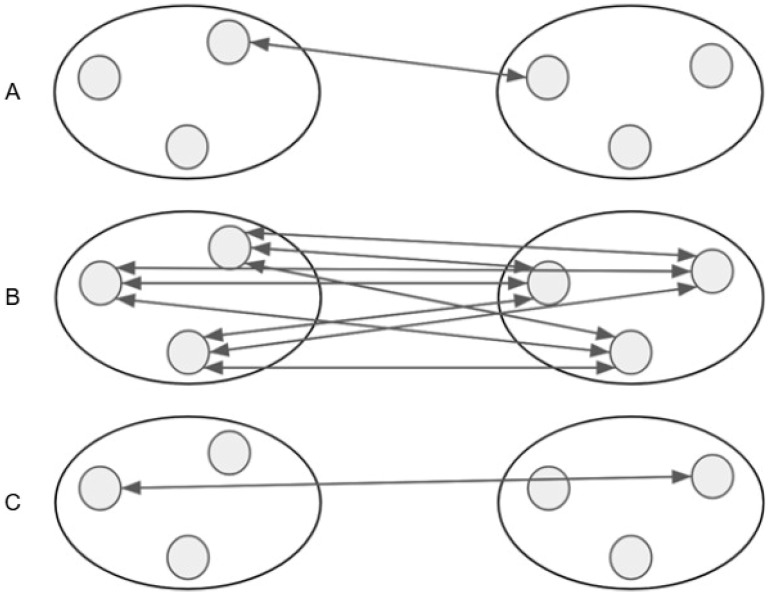
Linkage parameter values description (arrows show which distances between cluster elements are measured to obtain distance between clusters): (**A**) Single linkage refers to the distance between clusters defined as the smallest distance taken over all pairs of clusters representatives. (**B**) Average linkage refers to the average distance between all pairs of clusters representatives. (**C**) Complete linkage refers to the biggest distance between any pair of clusters representatives.

We used clustering methods implemented in the scikit-learn library (version 1.8.0) for the Python programming language, which are capable of processing input data in the form of a distance matrix. These methods included Agglomerative Clustering—a hierarchical clustering approach based on a bottom-up strategy realized as follows. Initially, each object is treated as an independent cluster. Then clusters with the smallest distance between them are merged iteratively until a single common cluster is formed. As a result, hierarchical clustering using the agglomerative clustering method produces a binary tree, where the leaves represent the clustered objects [20,21]. The distance between clusters is determined by the distances between their elements and the linkage parameter, which can take the values “single”, “average”, or “complete” (Figure 6). Let *P* = {*p*_1_, *p*_2_, …, *p_s_*} and *Q* = {*q*_1_, *q*_2_, …, *q_t_*} be two arbitrary clusters; *d* is a distance function defined on *P* and *Q*. Single linkage corresponds to the distance between *P* and *Q* defined as *D*(*P*, *Q*) = *min* {*d*(*p*, *q*): *p* ∈ *P*, *q* ∈ *Q*}. Average linkage corresponds to the distance between *P* and *Q* defined as $D(P,\;Q)=\;\frac{\sum_{p\in P}\sum_{q\in Q}d\left(p,\;q\right)}{\left|P|\times |Q\right|}$. Complete linkage corresponds to the distance between *P* and *Q* defined as *D*(*P*, *Q*) = *max*{*d*(*p*, *q*): *p* ∈ *P*, *q* ∈ *Q*}.

We also used the following non-hierarchical clustering methods: Affinity Propagation [31], DBSCAN [32], OPTICS [33], HDBSCAN [34].

All the mentioned clustering methods (including Agglomerative Clustering) use only a distance matrix as input. Different distance matrices are generated by calculating distance measures listed in Section 4.1 between the vector representations of the proteomes from our dataset (Section 4.2). Information about clade affiliations is not used.

### 4.4. Clustering Assessment

To assess the quality of clustering, we used the Rand index [35] conventionally used to estimate the performance in clustering tasks. The Rand index compares the estimated clustering with a known reference classification. To do this, all possible pairs of elements of the clusterized set are considered. *TP* (true positives) is the number of pairs of elements belonging to the same cluster and the same class, *FP* (false positives)—to the same cluster, but different classes, *FN* (false negatives)—to different clusters, but the same class, and *TN* (true negative)—to different clusters and different classes. The Rand index shows which fraction of all possible pairs of elements of the clusterized set are correctly positioned relative to each other (that is, they are *TP* or *TN*): $Rand=\frac{TP\;+\;TN}{TP\;+\;FP\;+\;FN\;+\;TN}$, Rand ∈ [0, 1], where Rand = 1 means a complete match of the estimated clustering with the correct classification. We use the term “accuracy” as a synonym of the Rand index.

**Figure 7:**
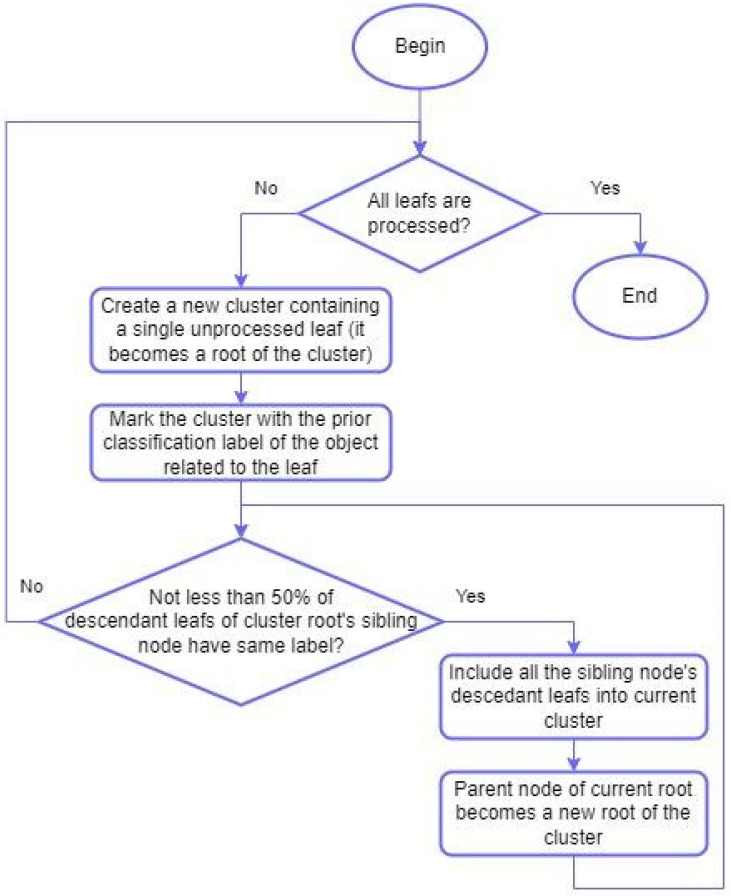
Algorithm for conversion of hierarchical clustering to non-hierarchical one.

In order to be able to compare the Agglomerative Clustering method with other clustering methods using the Rand index, we converted hierarchical clustering to non-hierarchical one by the following algorithm, which uses reference classification data to optimally limit cluster sizes during the conversion. It should be noted that these data are not used directly in the clustering, so we consider the comparison of results using the Rand index in this context to be valid. A generalized flowchart of the algorithm is presented below (Figure 7). The number of clusters may not match the number of actual classes due to imperfect clustering.

### 4.5. Clustering Feasibility

In this work, clustering is performed based on a distance matrix between proteomes or between proteins within a proteome. To evaluate the possibility of reasonable clustering, we compared the average distance between elements within a cluster (which characterizes the dispersion of the cluster’s point cloud) with the average distance between elements of different clusters. For high-quality clustering, the former should be much smaller than the latter. Let the set *Q*, which we aim to clusterize, consist of *t* reference classes (disjoint subsets): *Q*_1_∪*Q*_2_∪…∪ *Q_t_* = *Q*, *∀I* ∈ [*1*, *t*], *∀j* ∈ [*1*, *i*)∪(*i*, *t*]; *Q_i_*∩*Q_j_* = *∅*. Let *N_i_* be the number of elements in *Q_i_*. Then, the average within-group distance *D_i_* for *Q_i_* is calculated using the formula:

(1) $$D_{i}=\frac{2\sum_{p,q\in Q_{i},p\neq q}d(p,q)}{N_{i}\left(1+N_{i}\right)}$$

Here, *d*(*p*, *q*) is the distance between elements *p* and *q*. The average between-group distance *D_ij_* for *Q_i_* and *Q_j_* is calculated using the formula:

(2) $$D_{ij}=\frac{\sum_{p\in Q_{i},q\in Q_{j}}d\left(p,q\right)}{N_{i}N_{j}}$$

## 5. Conclusions

Our results, based on a comprehensive analysis of short amino acid k-mer frequency distributions across 86 bacterial proteomes from 18 clades, demonstrate that even a simple analysis of tripeptide frequencies can accurately position proteomes within k-mer space, which was shown in this study in post hoc manner. Evolutionary distances derived from k-mer comparisons allowed for the reconstruction of highly accurate phylogenetic trees, achieving up to 99% match with reference classification of the proteomes according to the Rand index. We evaluated various clustering parameters and identified a set that yielded clusters closely matching natural phylogeny. These findings indicate that k-mer frequency-based proteomic analysis is a powerful and interpretable approach for evolutionary studies, providing a robust framework for uncovering evolutionary relationships.

## Figures and Tables

**Table 1:** Mean and standard deviation Rand index values obtained by fixing a single parameter value and varying other parameters. Parameter values giving the highest mean Rand index are highlighted in blue.

k		Alphabet		Protein Group		Metric		Linkage	
Value	Mean Rand	Value	Mean Rand	Value	Mean Rand	Value	Mean Rand	Value	Mean Rand
1	0.9649 ± 0.0043	20 symbols	0.9706 ± 0.0067	all	0.9668 ± 0.0054	*L*1	0.9712 ± 0.0055	single	0.9668 ± 0.0061
2	0.9670 ± 0.0058	5 symbols	0.9640 ± 0.0049	membrane	0.9679 ± 0.0057	*L*2	0.9693 ± 0.0051	average	0.9680 ± 0.0073
3	0.9701 ± 0.0082			non—membrane	0.9663 ± 0.0048	*corr*	0.9691 ± 0.0056	complete	0.9677 ± 0.0068
				nucleotide—binding	0.9676 ± 0.0068	*inter*_0_._05_	0.9629 ± 0.0063		
				non—nucleotide—binding	0.9666 ± 0.0056	*inter*_0_._1_	0.9637 ± 0.0074		
				ribosomal	0.9700 ± 0.0103				

**Table 2:** Top 5 Agglomerative Clustering parameter combinations by Rand index (among all the considered protein groups and for complete proteome).

#	Rand	*k*	Alphabet	Protein Group	Metric	Linkage
1	0.9937	3	20 symbols	ribosomal	*corr*	average
2	0.9929	3	20 symbols	ribosomal	*L*1	average
3	0.9912	3	20 symbols	ribosomal	*L*2	average
4	0.9902	3	20 symbols	ribosomal	*L*1	complete
5	0.9899	3	20 symbols	ribosomal	*inter*_0_._1_	average
1	0.9776	3	20 symbols	all	*L*1	average
2	0.9762	3	20 symbols	all	*inter*_0_._1_	average
3	0.9748	3	20 symbols	all	*inter*_0_._1_	complete
4	0.9743	2	20 symbols	all	*L*1	average
5	0.9737	3	20 symbols	all	*corr*	complete
		2	20 symbols	all	*L*2	average

**Table 3:** Common most widespread k-mers among 86 bacteria.

Protein Group	Number (Out of 800)
All	226
Membrane	261
Non-membrane	191
Nucleotide-binding	155
Non-nucleotide-binding	228
Ribosomal	56

**Table 4:** Table of within-group distances in protein groups averaged over 18 proteomes.

Proteins Set	Average Within-Group Distance
All	1.893
Membrane	1.861
Non membrane	1.898
Nucleotide binding	1.889
Non-nucleotide binding	1.892
Ribosomal	1.932
Non ribosomal	1.891

**Table 5:** Table of between-group distances between protein groups averaged over 18 proteomes.

	All	Membrane	Non-Membrane	Nucleotide Binding	Non-Nucleotide Binding	Ribosomal	Non-Ribosomal
All	—	1.883	1.895	1.893	1.892	1.926	1.891
Membrane	1.883	—	1.891	1.890	1.882	1.927	1.882
Non-membrane	1.895	1.891	—	1.894	1.895	1.926	1.894
Nucleotide binding	1.893	1.890	1.894	—	1.895	1.919	1.893
Non-nucleotide binding	1.892	1.882	1.895	1.895	—	1.927	1.891
Ribosomal	1.926	1.927	1.926	1.919	1.927	—	1.927
Non-ribosomal	1.891	1.882	1.894	1.893	1.891	1.927	—

## Data Availability

The original contributions presented in this study are included in the article/Supplementary Material. Further inquiries can be directed to the corresponding author.

## Supplementary Materials

The following supporting information can be downloaded at: https://www.mdpi.com/article/10.3390/ijms27010109/s1. Data referenced in the article is provided as kmers_supplementary.zip archive file. The code applied to obtain presented results is available at https://gitlab.com/WingeDD/kmer_prot_repr (accessed on 18 December 2025).
