# Supplementary figures and images for: Tracking Down the Evolution of Microorganisms by Exhaustive Bottom-Up Analysis of Proteomes (part 1 of 2)
Source: Int J Mol Sci. 2025 Dec 22;27(1):109. doi: 10.3390/ijms27010109 (PMC12785394; doi:10.3390/ijms27010109)

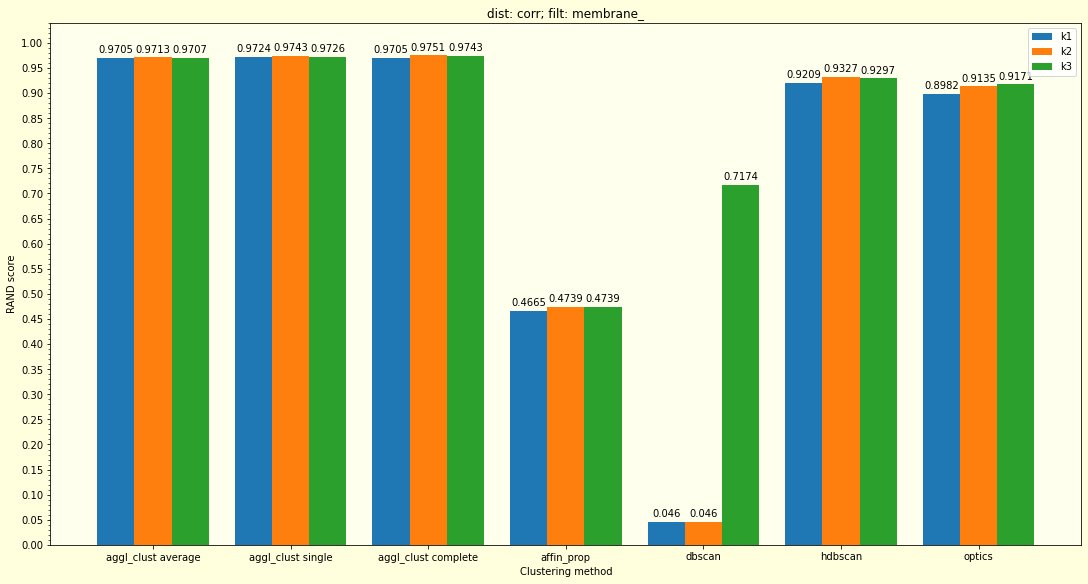

Supplement: Supplementary file 1 [file ijms-27-00109-s001.zip › kmers_supplementary/clustering_scores/corr_charts/membrane_.png]

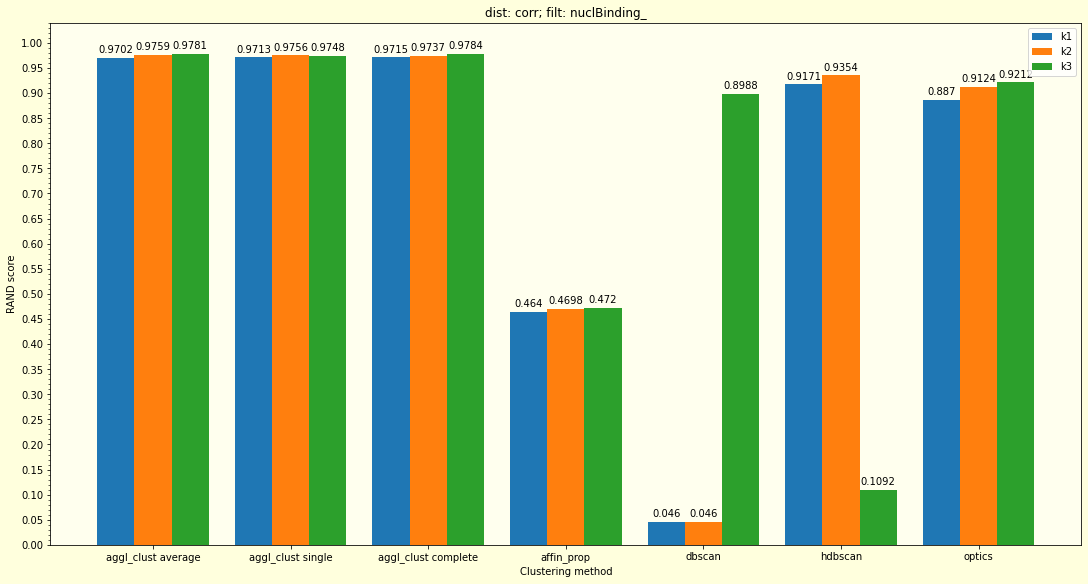

Supplement: Supplementary file 1 [file ijms-27-00109-s001.zip › kmers_supplementary/clustering_scores/corr_charts/nuclBinding_.png]

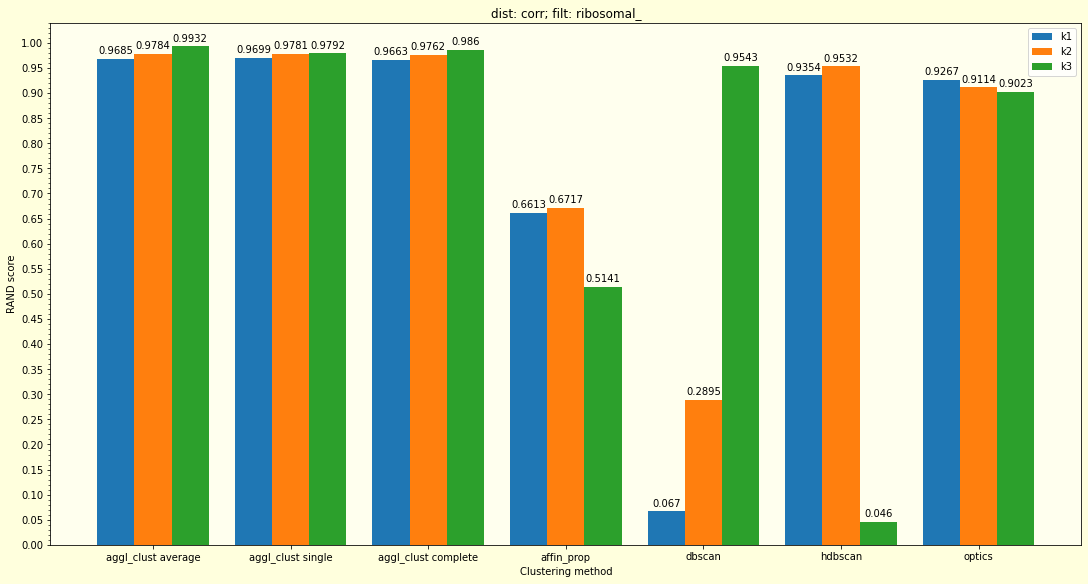

Supplement: Supplementary file 1 [file ijms-27-00109-s001.zip › kmers_supplementary/clustering_scores/corr_charts/ribosomal_.png]

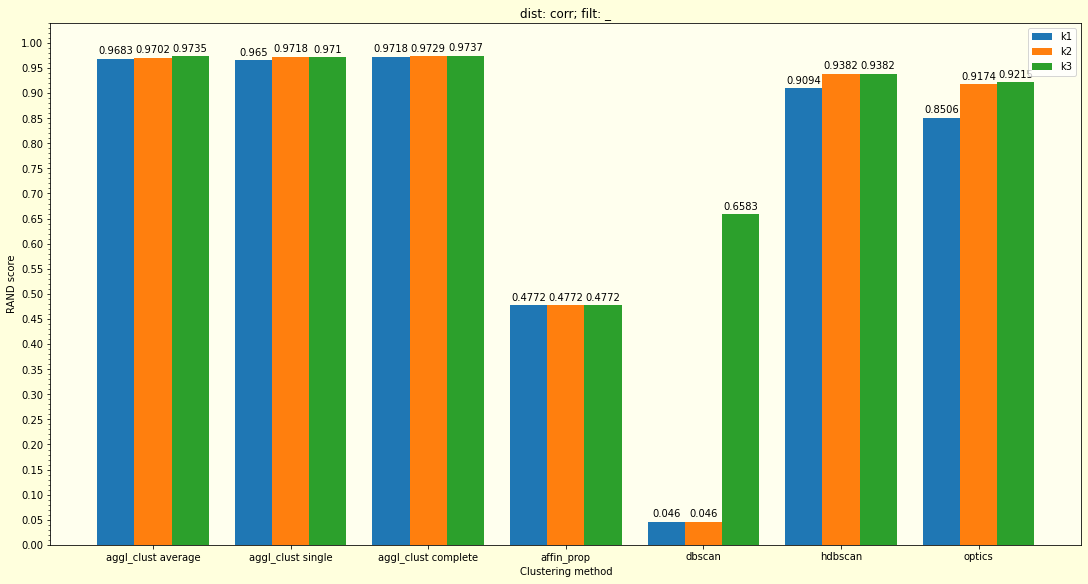

Supplement: Supplementary file 1 [file ijms-27-00109-s001.zip › kmers_supplementary/clustering_scores/corr_charts/_.png]

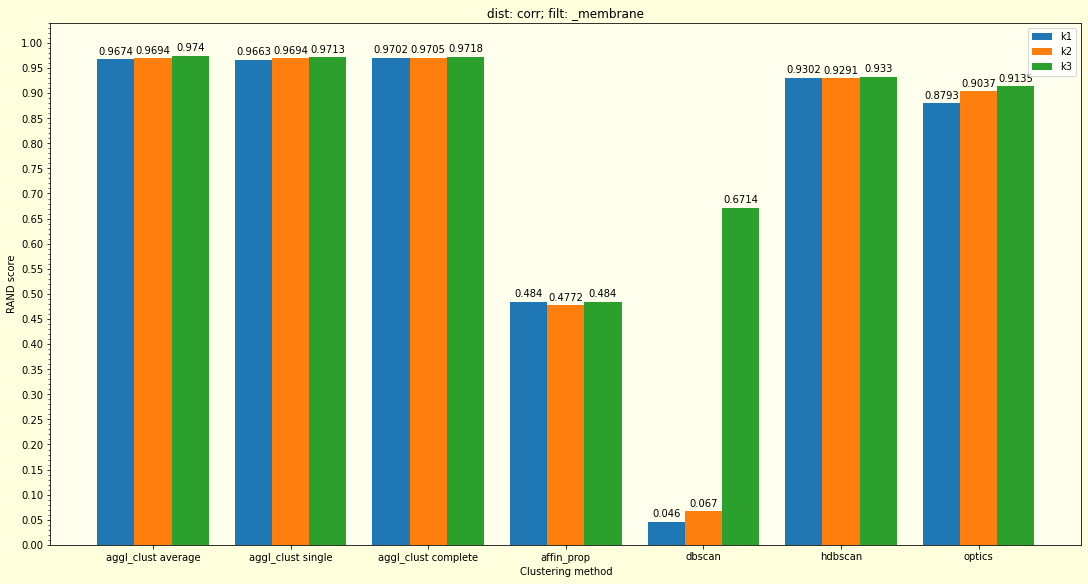

Supplement: Supplementary file 1 [file ijms-27-00109-s001.zip › kmers_supplementary/clustering_scores/corr_charts/_membrane.png]

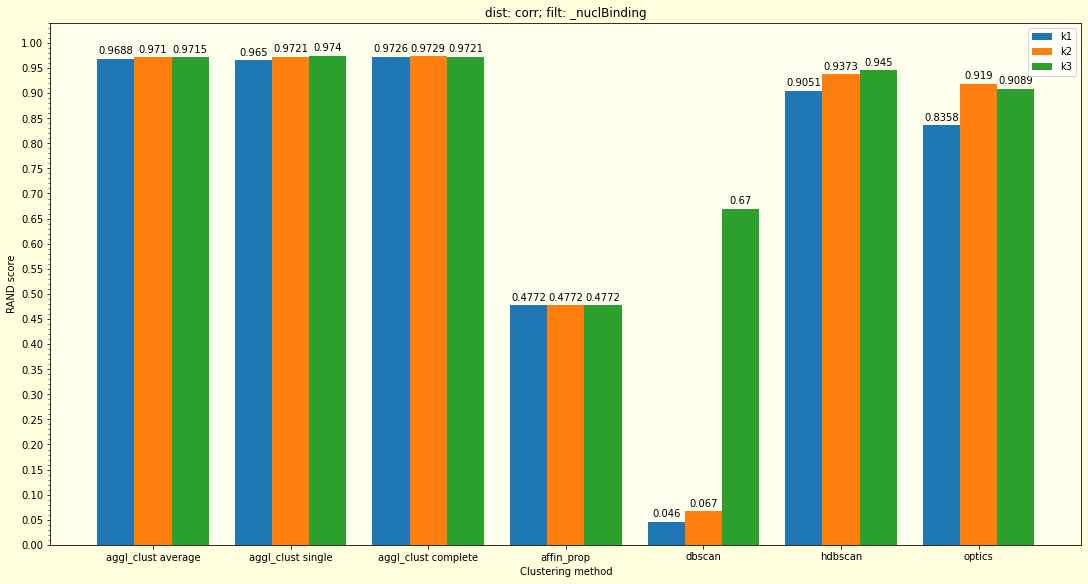

Supplement: Supplementary file 1 [file ijms-27-00109-s001.zip › kmers_supplementary/clustering_scores/corr_charts/_nuclBinding.png]

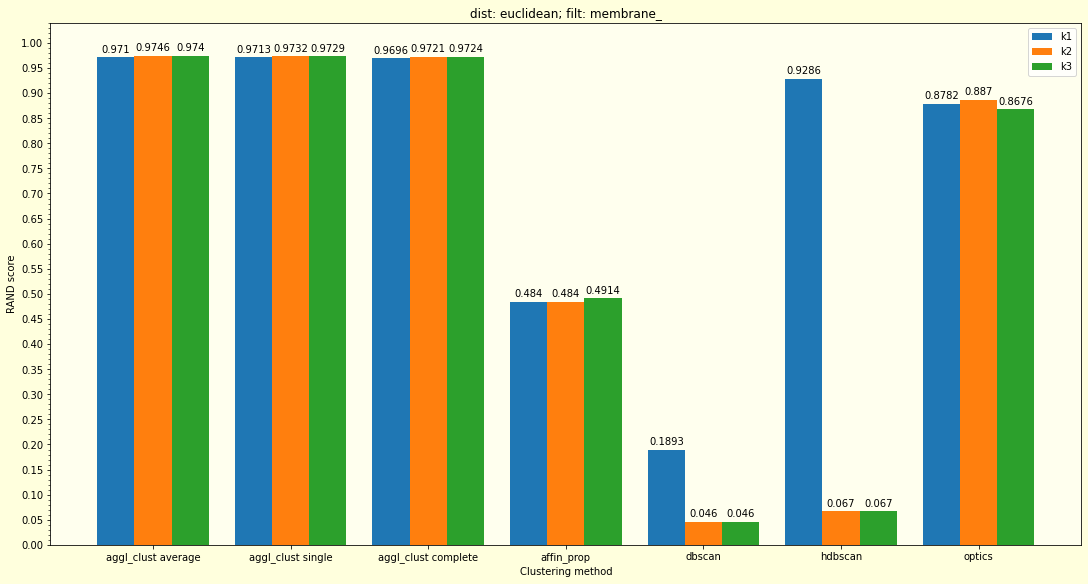

Supplement: Supplementary file 1 [file ijms-27-00109-s001.zip › kmers_supplementary/clustering_scores/euclidean_charts/membrane_.png]

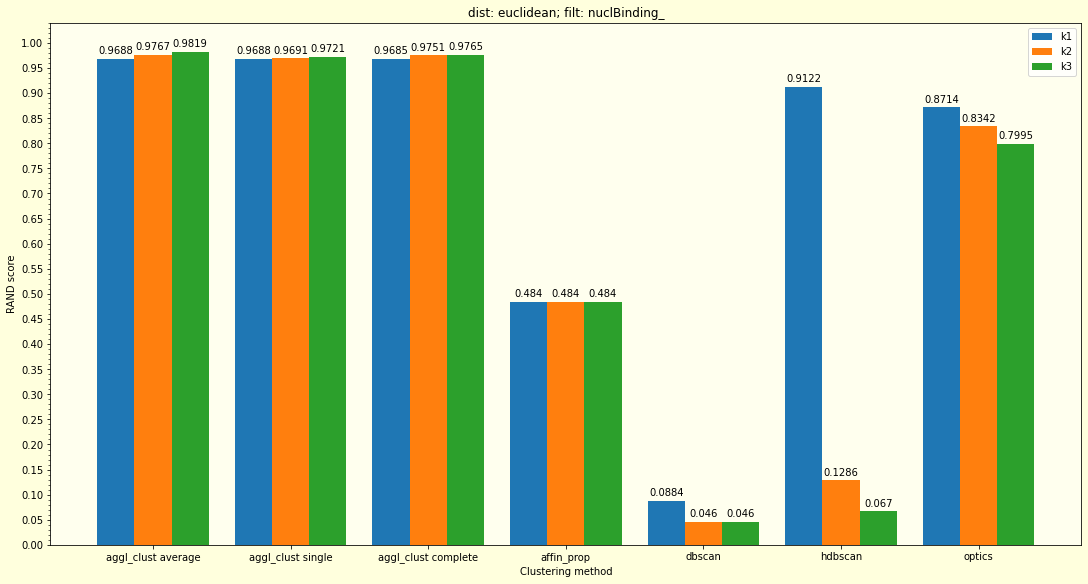

Supplement: Supplementary file 1 [file ijms-27-00109-s001.zip › kmers_supplementary/clustering_scores/euclidean_charts/nuclBinding_.png]

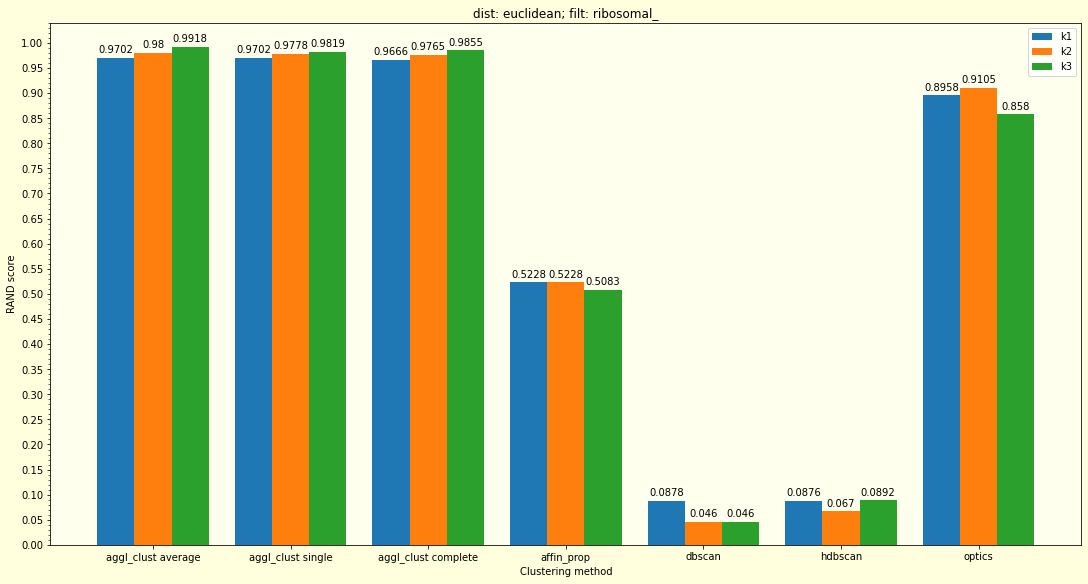

Supplement: Supplementary file 1 [file ijms-27-00109-s001.zip › kmers_supplementary/clustering_scores/euclidean_charts/ribosomal_.png]

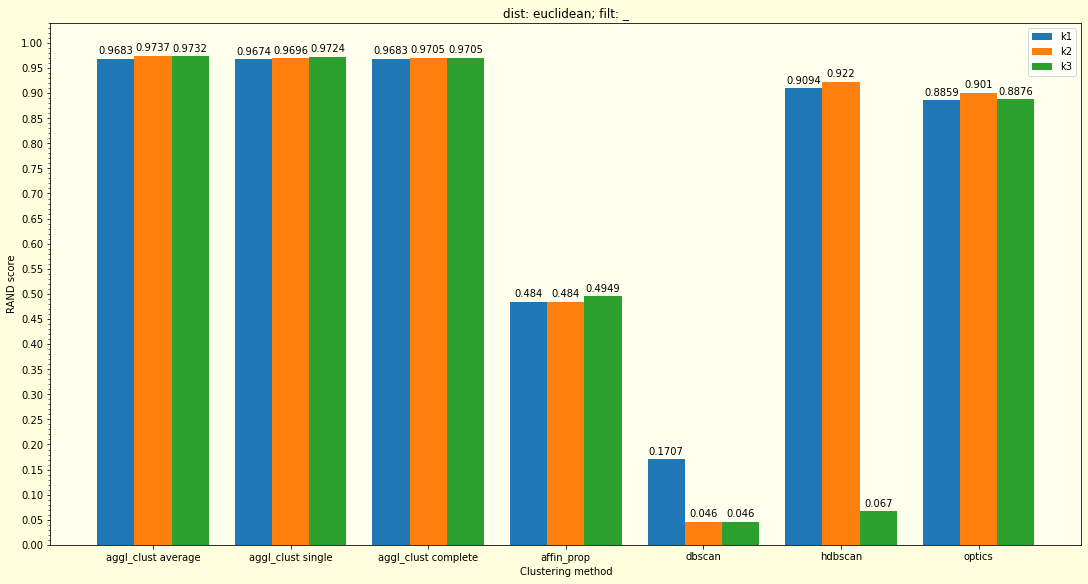

Supplement: Supplementary file 1 [file ijms-27-00109-s001.zip › kmers_supplementary/clustering_scores/euclidean_charts/_.png]

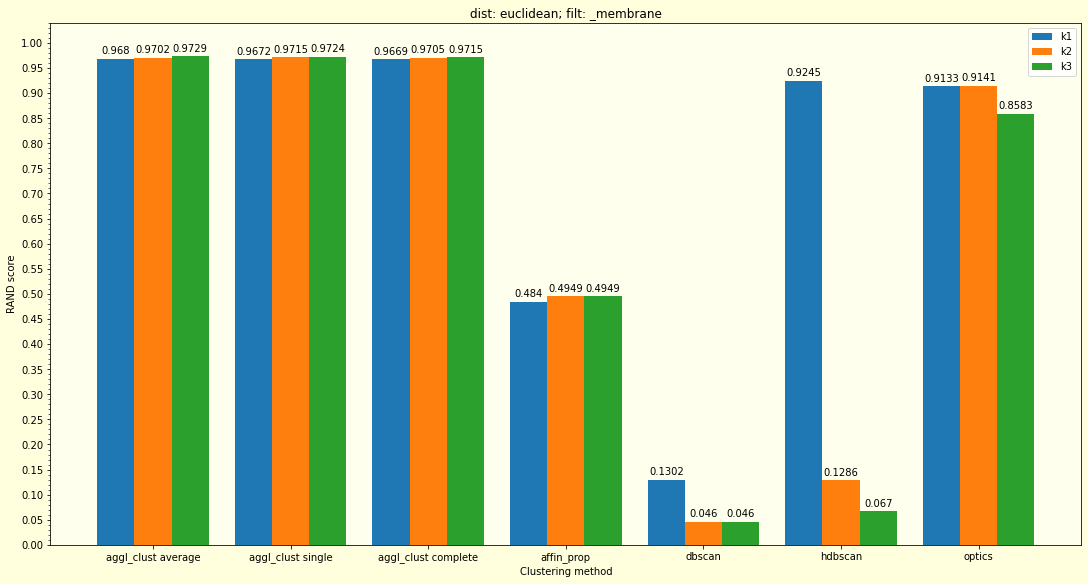

Supplement: Supplementary file 1 [file ijms-27-00109-s001.zip › kmers_supplementary/clustering_scores/euclidean_charts/_membrane.png]

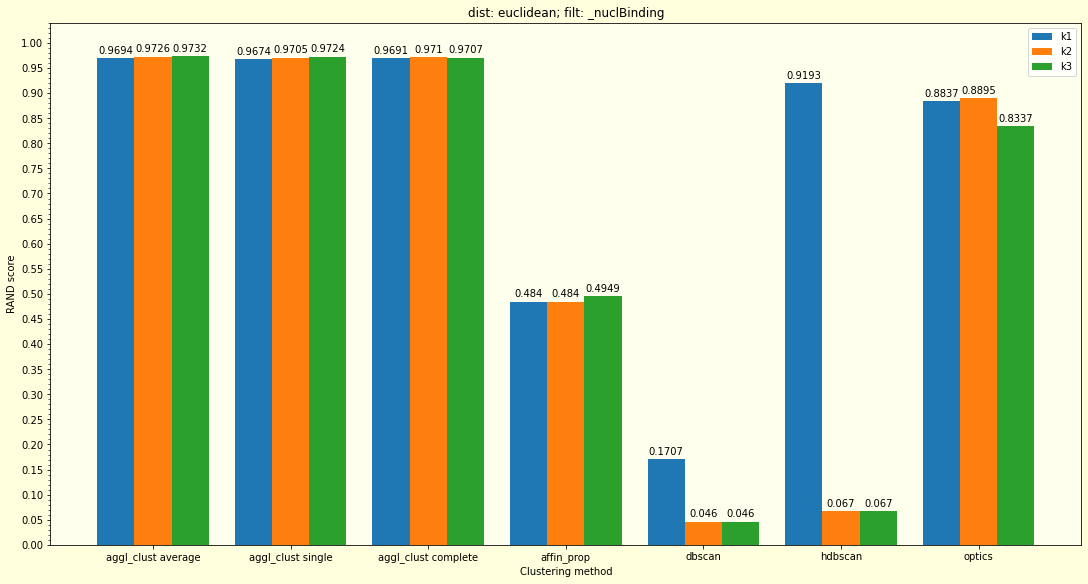

Supplement: Supplementary file 1 [file ijms-27-00109-s001.zip › kmers_supplementary/clustering_scores/euclidean_charts/_nuclBinding.png]

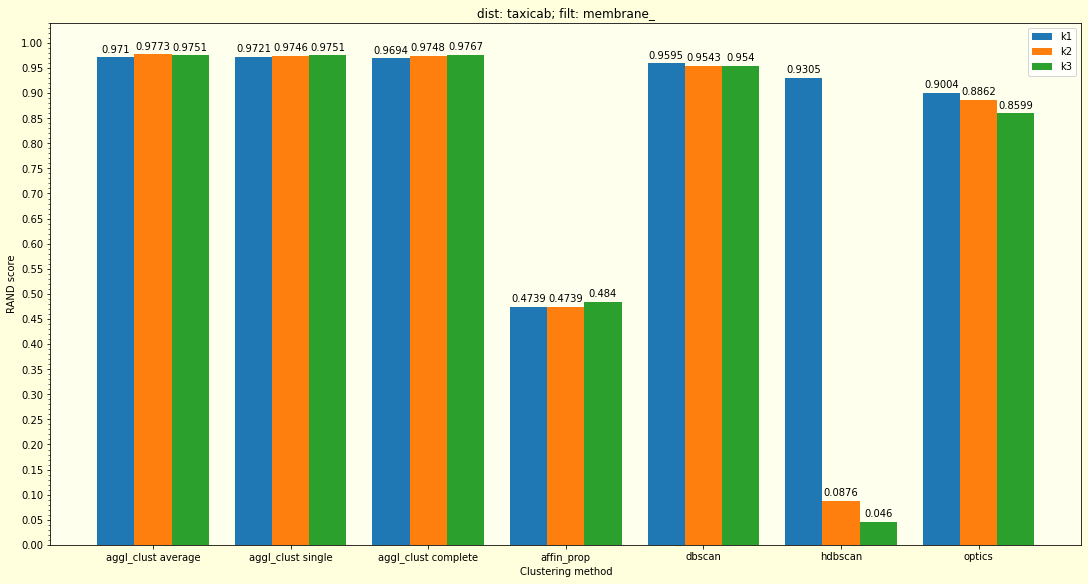

Supplement: Supplementary file 1 [file ijms-27-00109-s001.zip › kmers_supplementary/clustering_scores/taxicab_charts/membrane_.png]

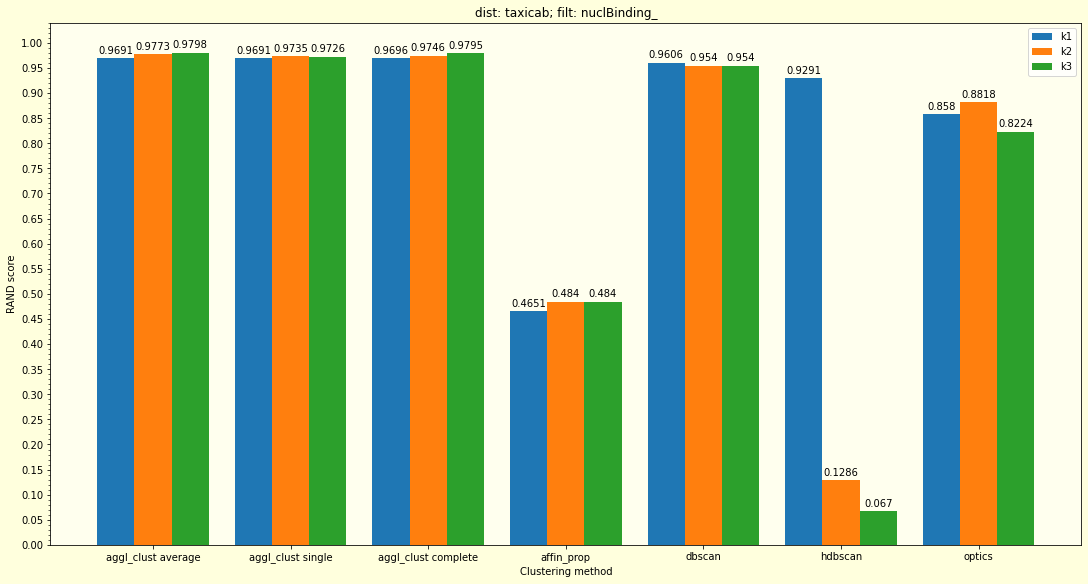

Supplement: Supplementary file 1 [file ijms-27-00109-s001.zip › kmers_supplementary/clustering_scores/taxicab_charts/nuclBinding_.png]

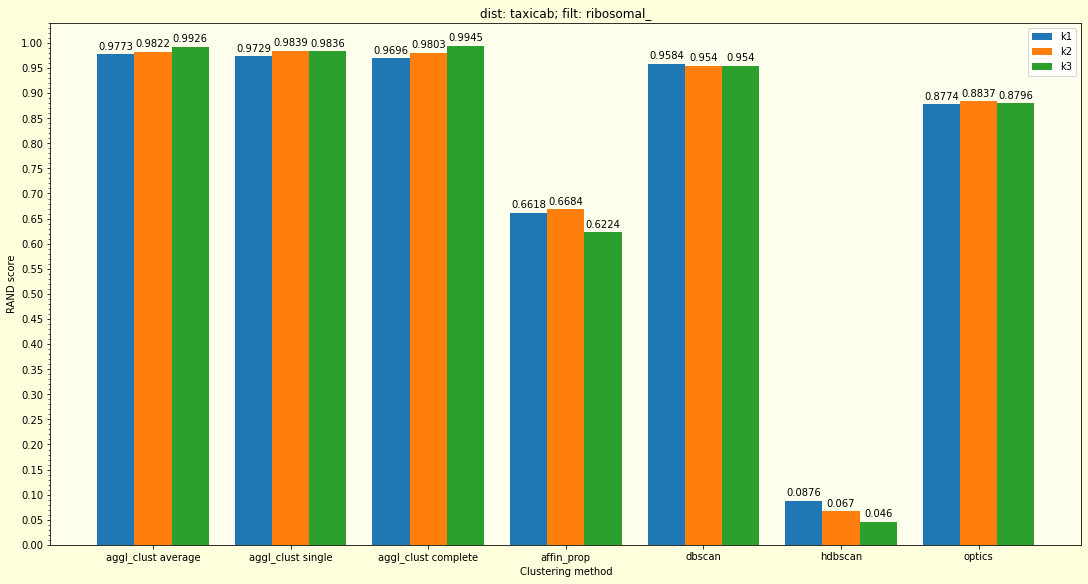

Supplement: Supplementary file 1 [file ijms-27-00109-s001.zip › kmers_supplementary/clustering_scores/taxicab_charts/ribosomal_.png]

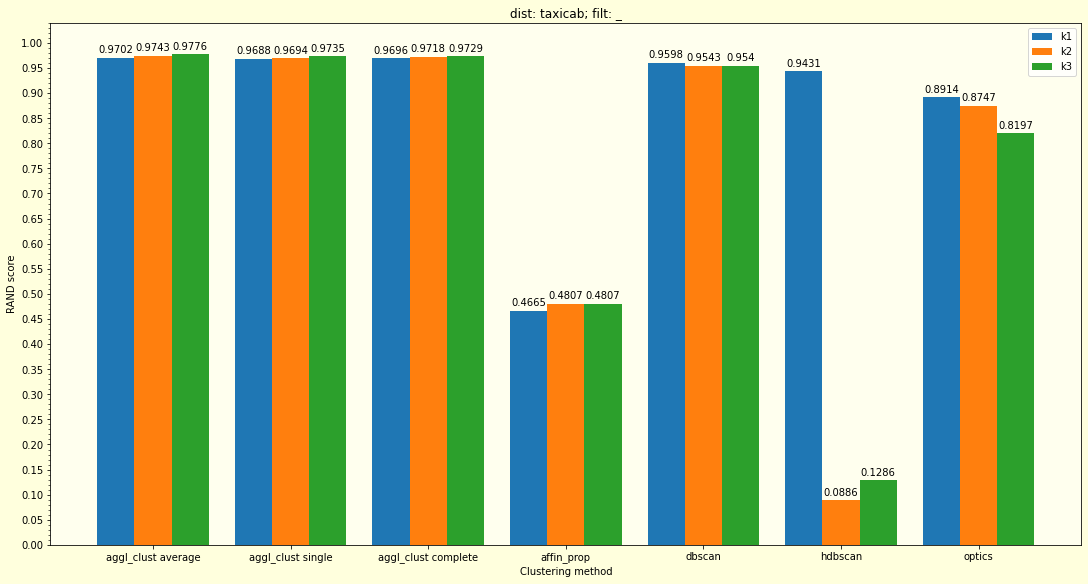

Supplement: Supplementary file 1 [file ijms-27-00109-s001.zip › kmers_supplementary/clustering_scores/taxicab_charts/_.png]

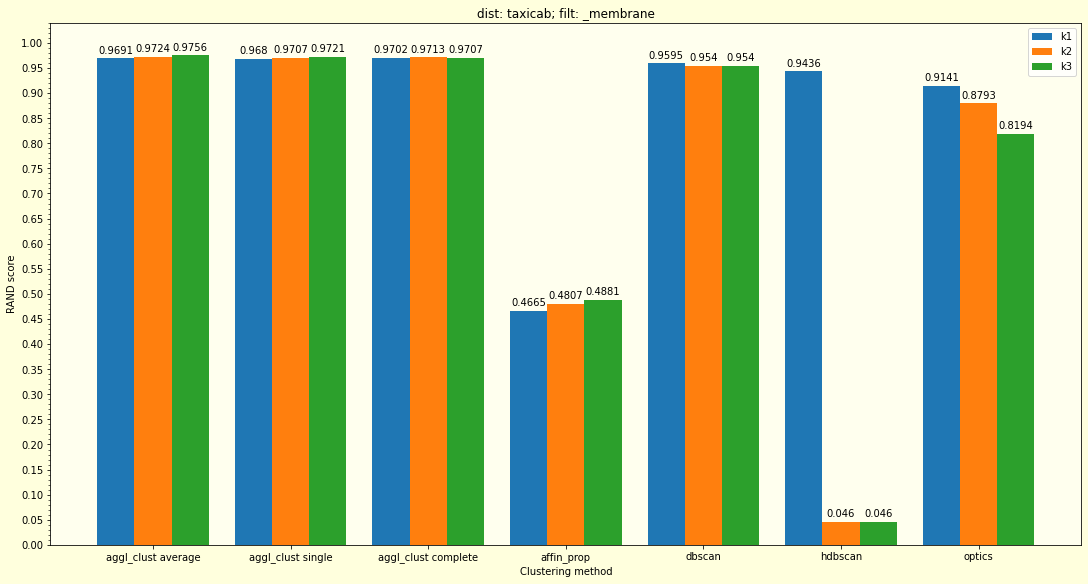

Supplement: Supplementary file 1 [file ijms-27-00109-s001.zip › kmers_supplementary/clustering_scores/taxicab_charts/_membrane.png]

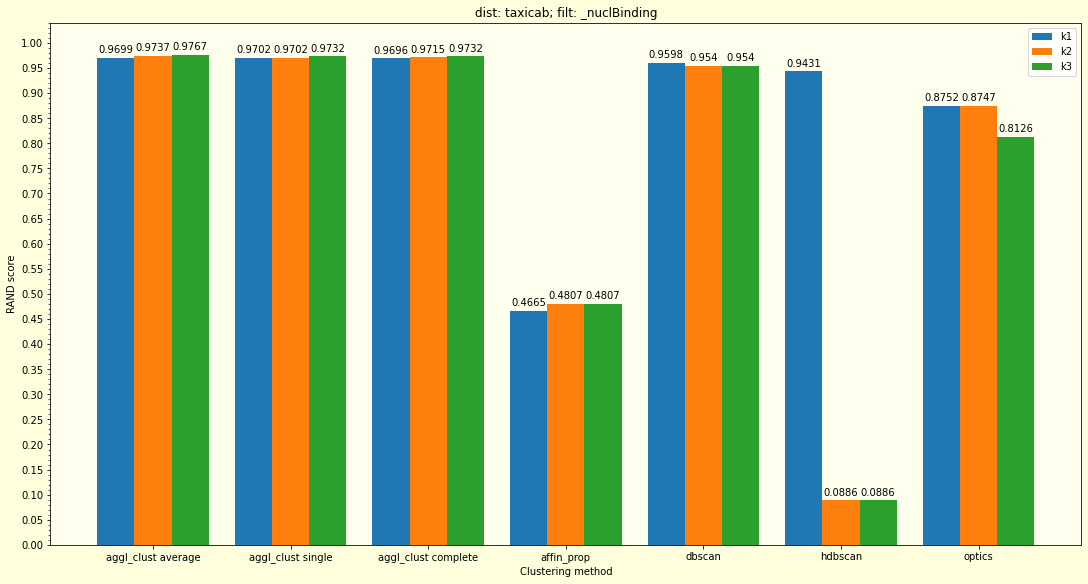

Supplement: Supplementary file 1 [file ijms-27-00109-s001.zip › kmers_supplementary/clustering_scores/taxicab_charts/_nuclBinding.png]

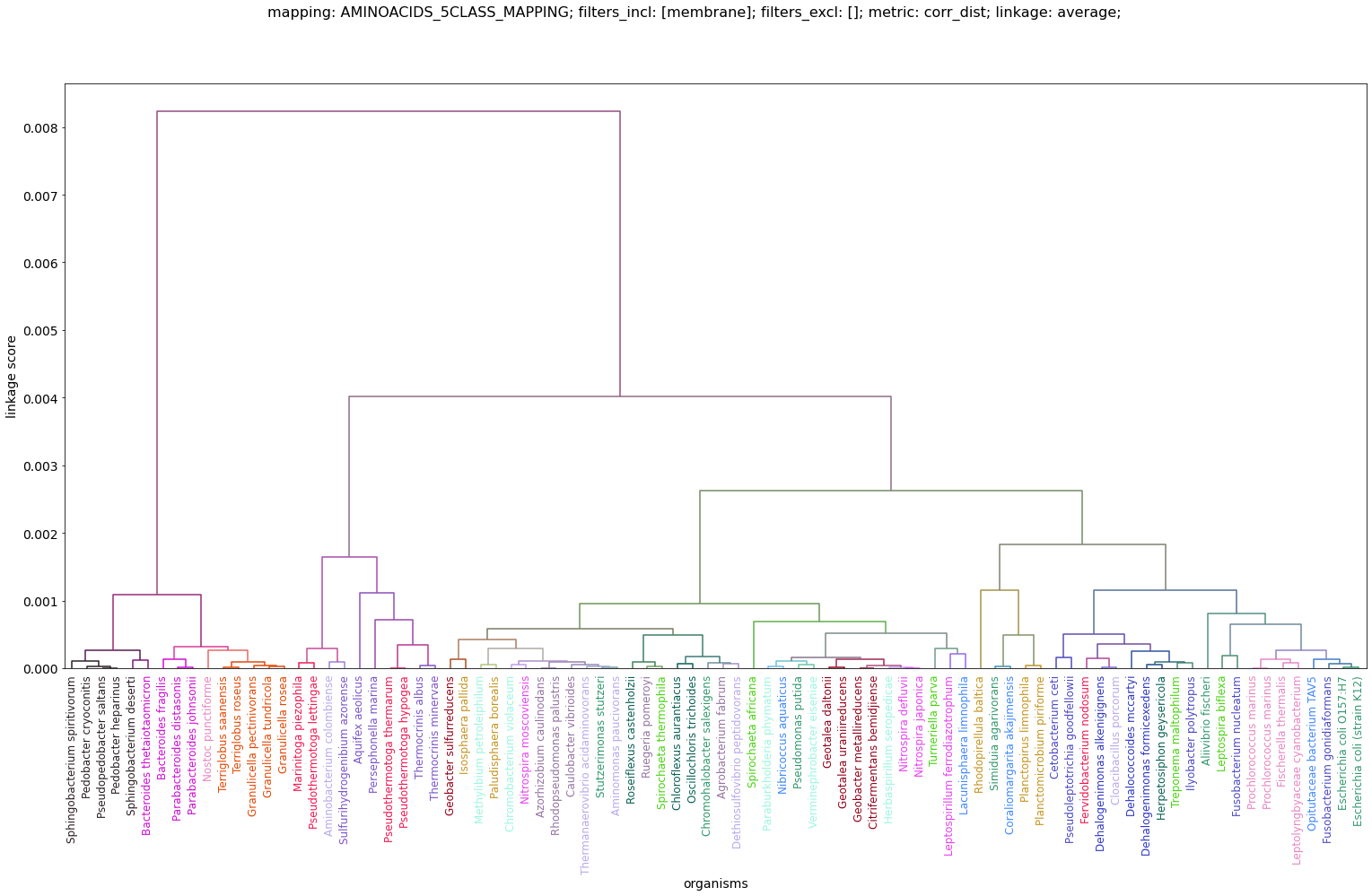

Supplement: Supplementary file 1 [file ijms-27-00109-s001.zip › kmers_supplementary/dendrograms/k1/AMINOACIDS_5CLASS_MAPPING/membrane_/corr_dist_average.png]

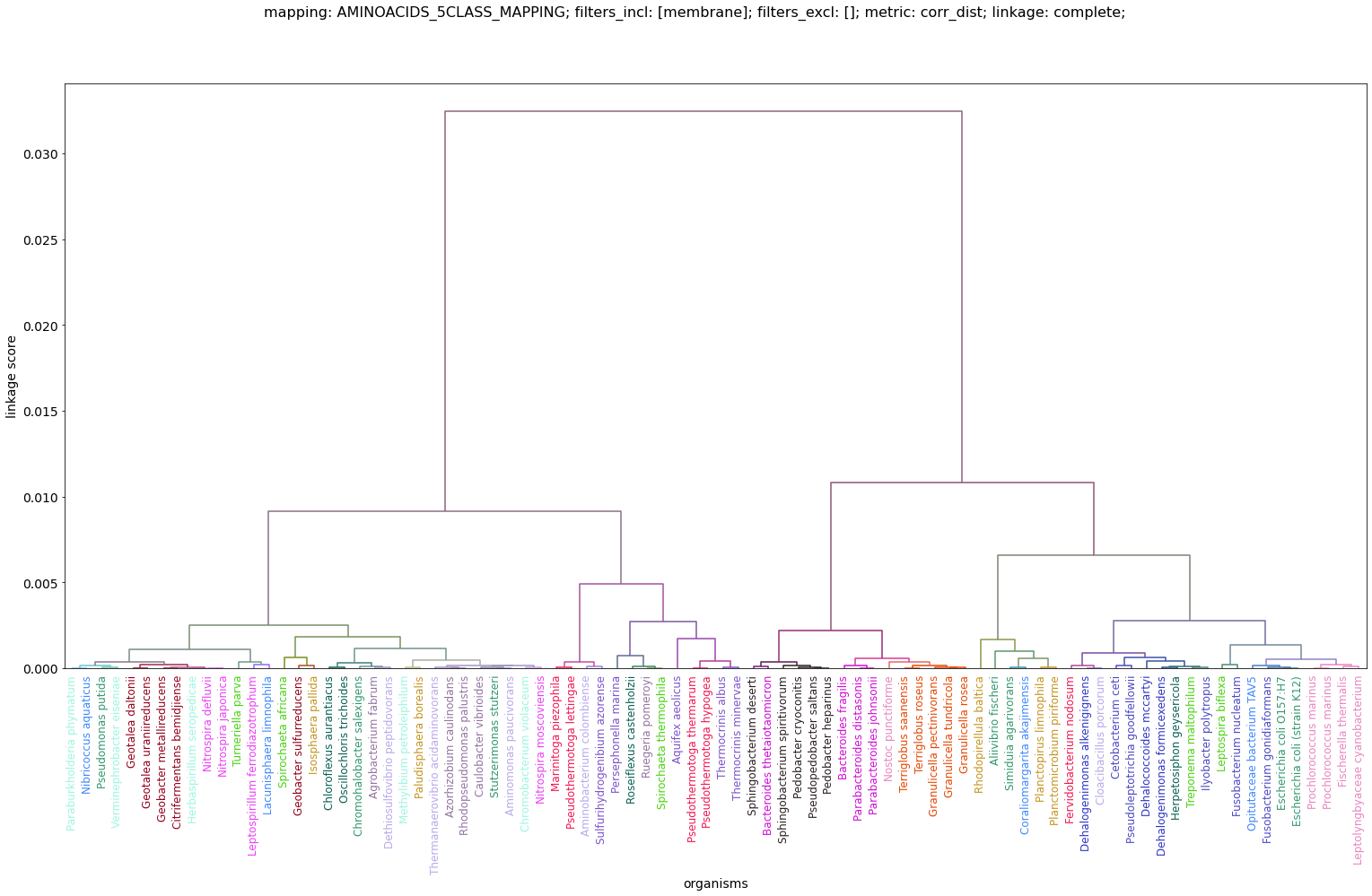

Supplement: Supplementary file 1 [file ijms-27-00109-s001.zip › kmers_supplementary/dendrograms/k1/AMINOACIDS_5CLASS_MAPPING/membrane_/corr_dist_complete.png]

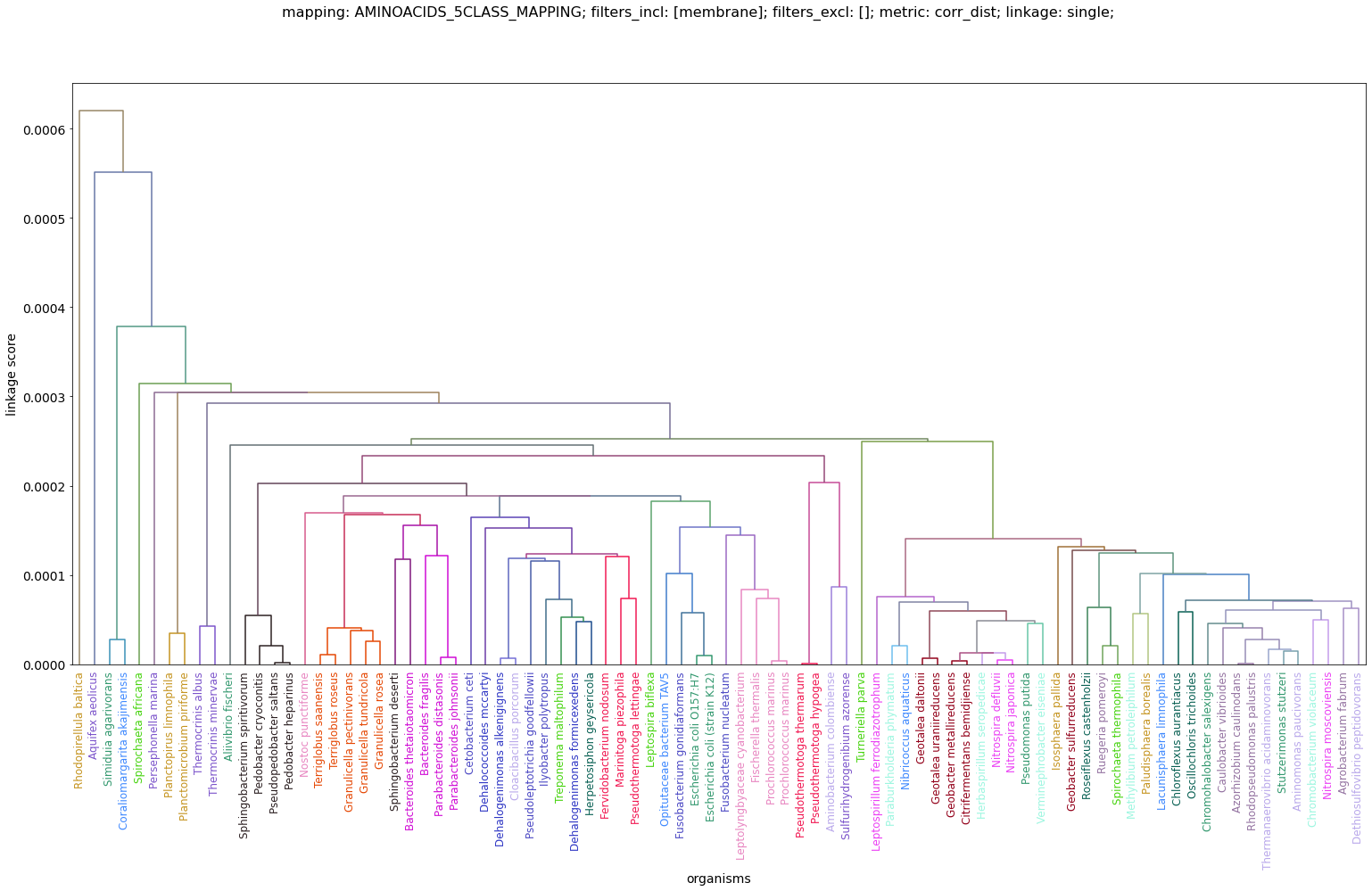

Supplement: Supplementary file 1 [file ijms-27-00109-s001.zip › kmers_supplementary/dendrograms/k1/AMINOACIDS_5CLASS_MAPPING/membrane_/corr_dist_single.png]

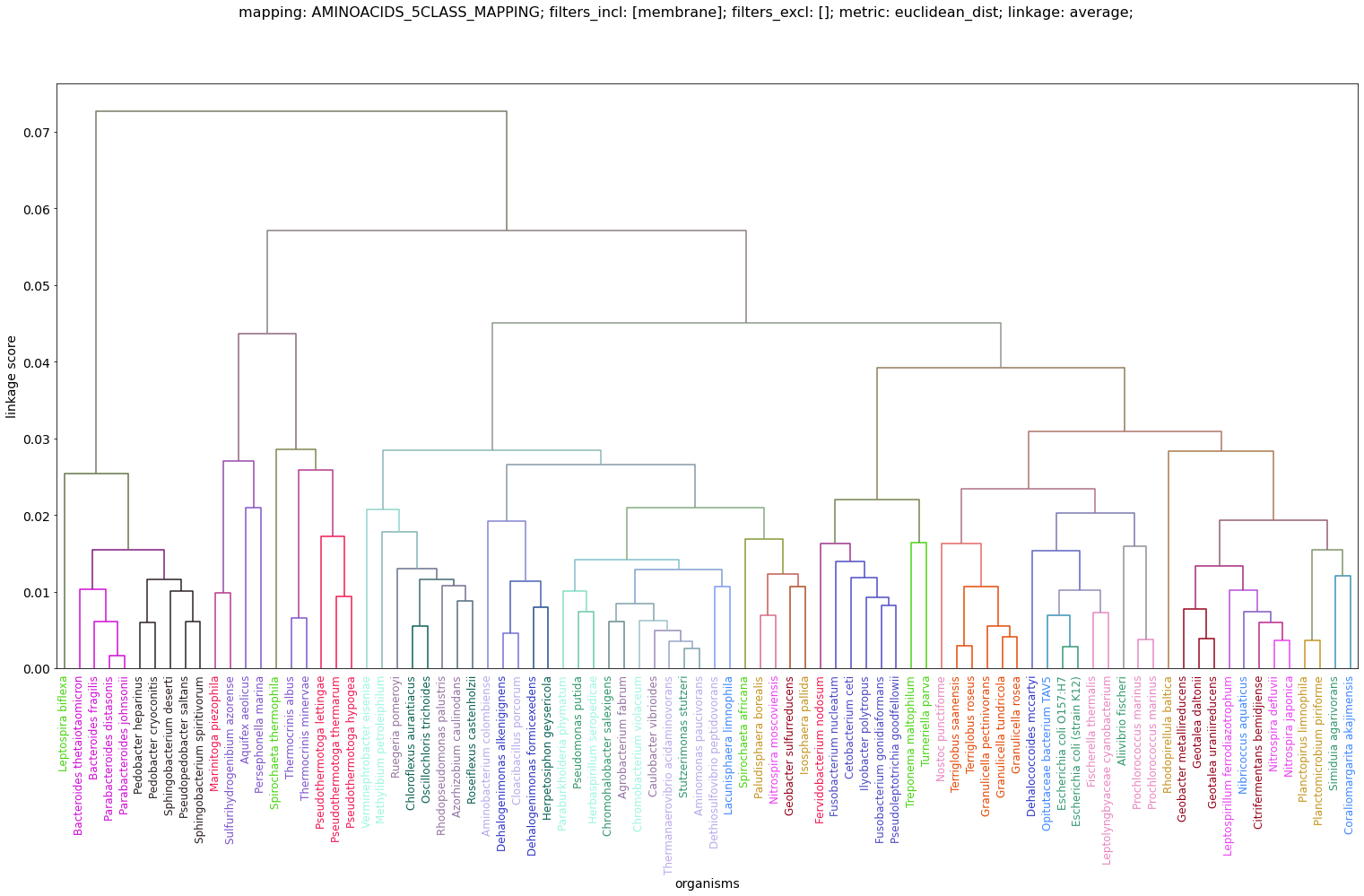

Supplement: Supplementary file 1 [file ijms-27-00109-s001.zip › kmers_supplementary/dendrograms/k1/AMINOACIDS_5CLASS_MAPPING/membrane_/euclidean_dist_average.png]

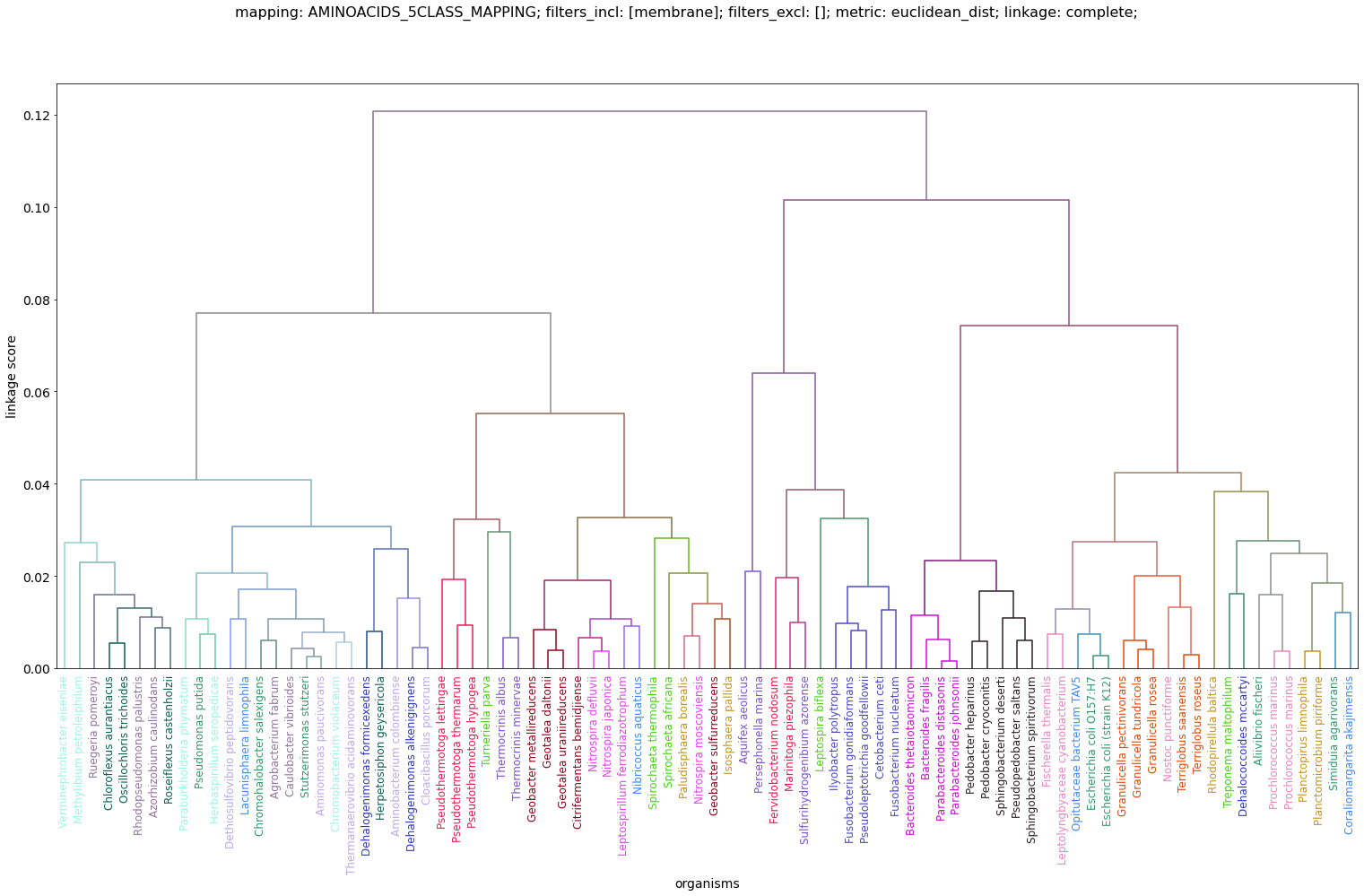

Supplement: Supplementary file 1 [file ijms-27-00109-s001.zip › kmers_supplementary/dendrograms/k1/AMINOACIDS_5CLASS_MAPPING/membrane_/euclidean_dist_complete.png]

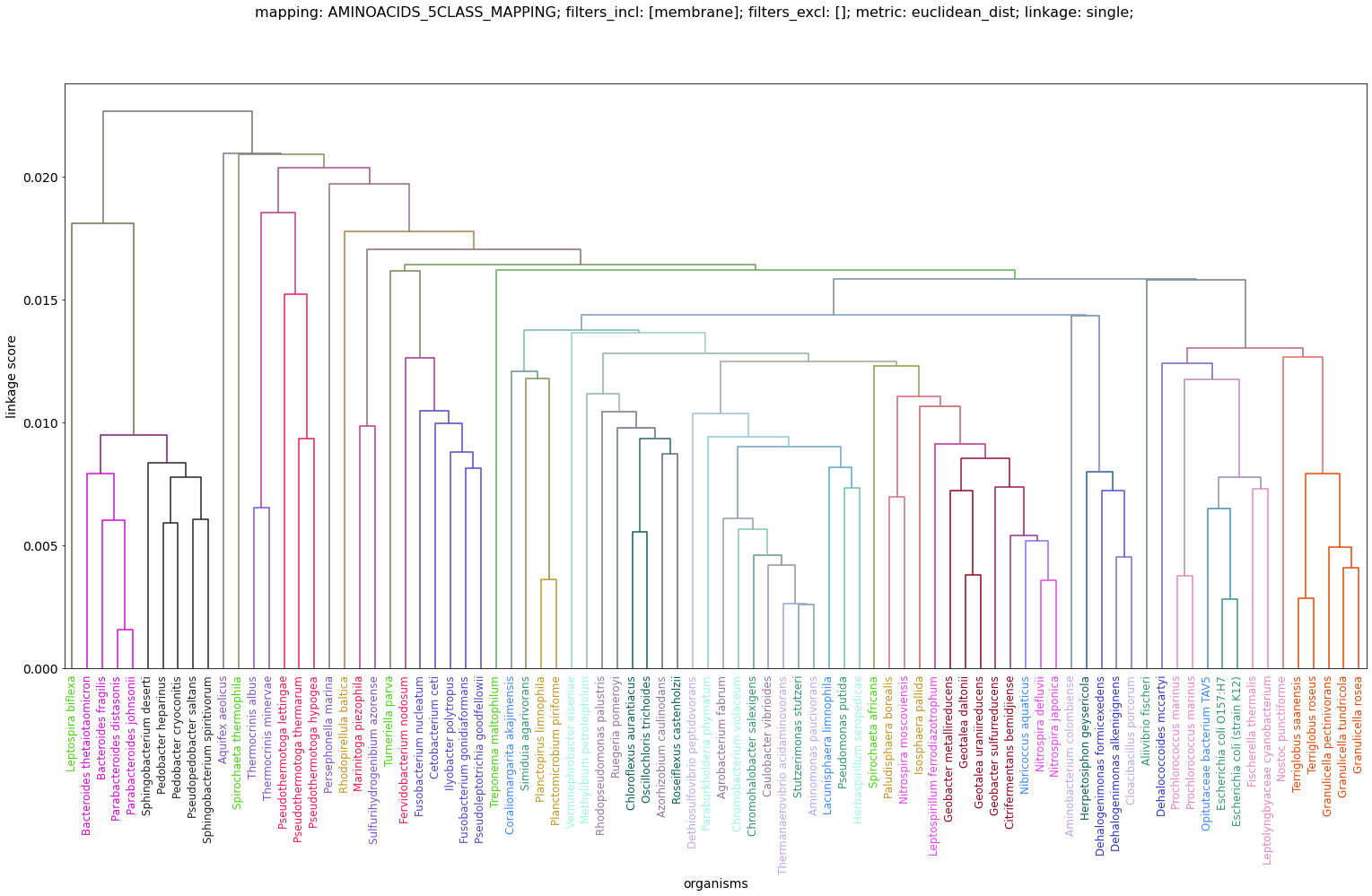

Supplement: Supplementary file 1 [file ijms-27-00109-s001.zip › kmers_supplementary/dendrograms/k1/AMINOACIDS_5CLASS_MAPPING/membrane_/euclidean_dist_single.png]

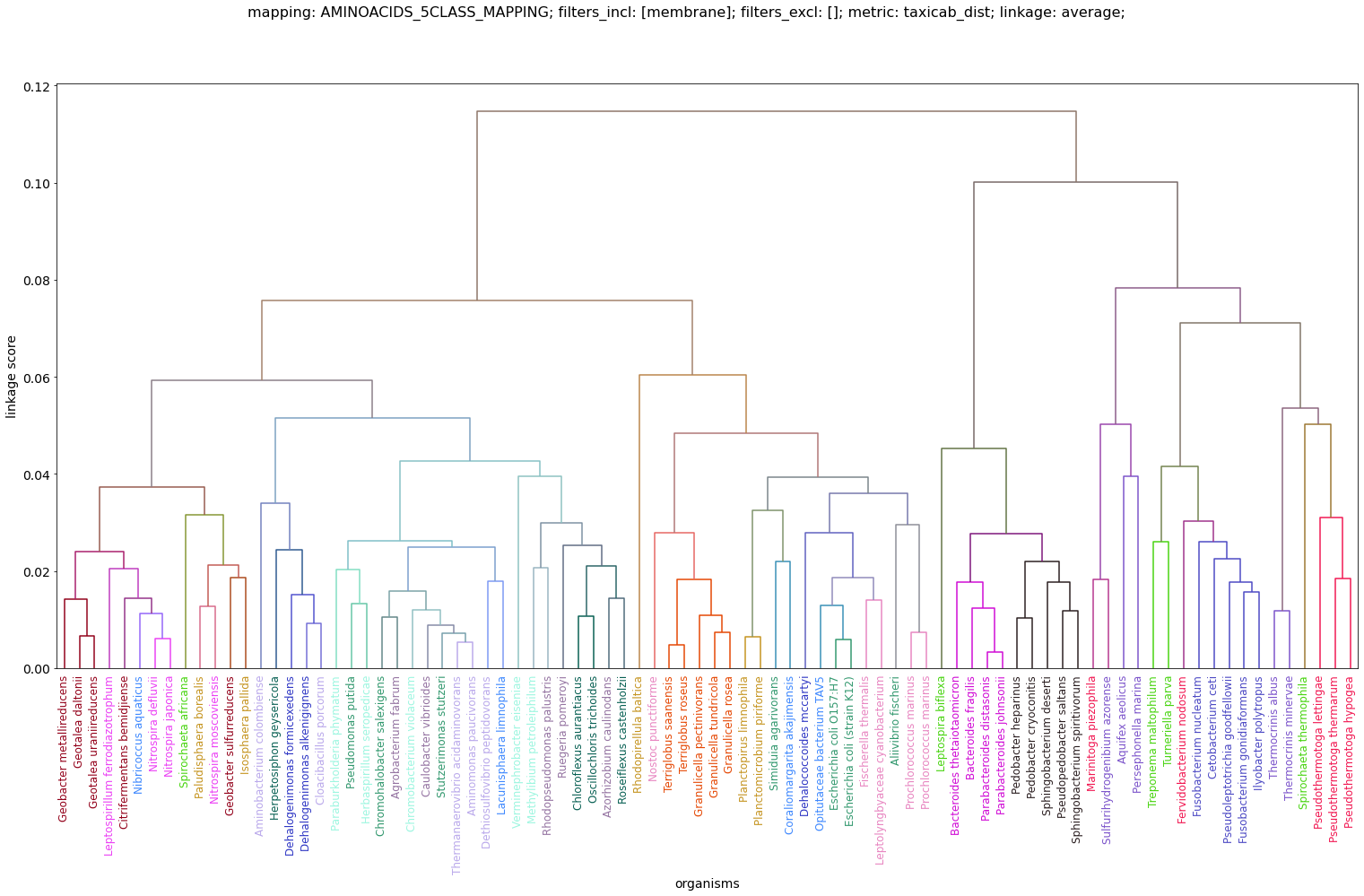

Supplement: Supplementary file 1 [file ijms-27-00109-s001.zip › kmers_supplementary/dendrograms/k1/AMINOACIDS_5CLASS_MAPPING/membrane_/taxicab_dist_average.png]

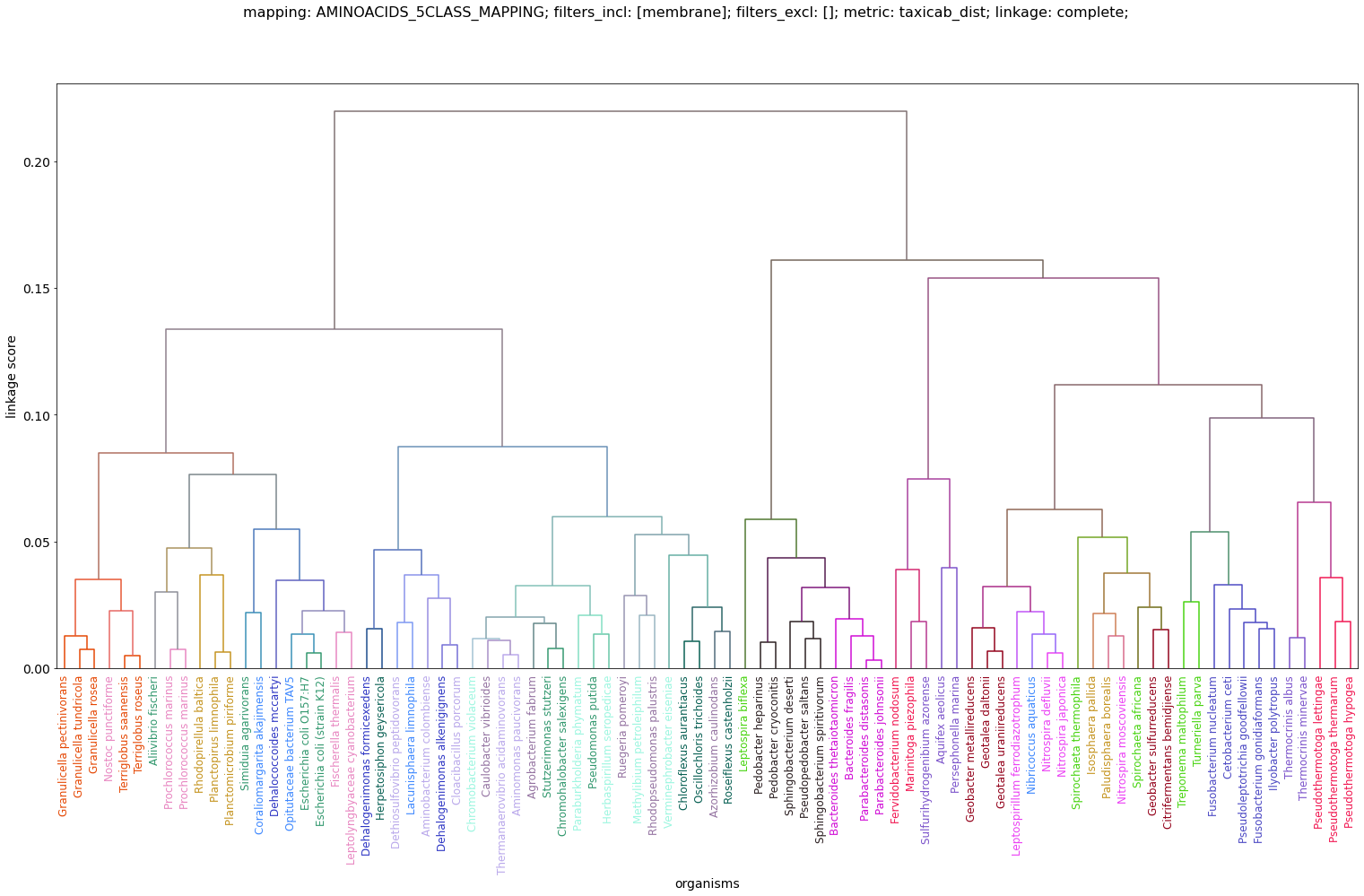

Supplement: Supplementary file 1 [file ijms-27-00109-s001.zip › kmers_supplementary/dendrograms/k1/AMINOACIDS_5CLASS_MAPPING/membrane_/taxicab_dist_complete.png]

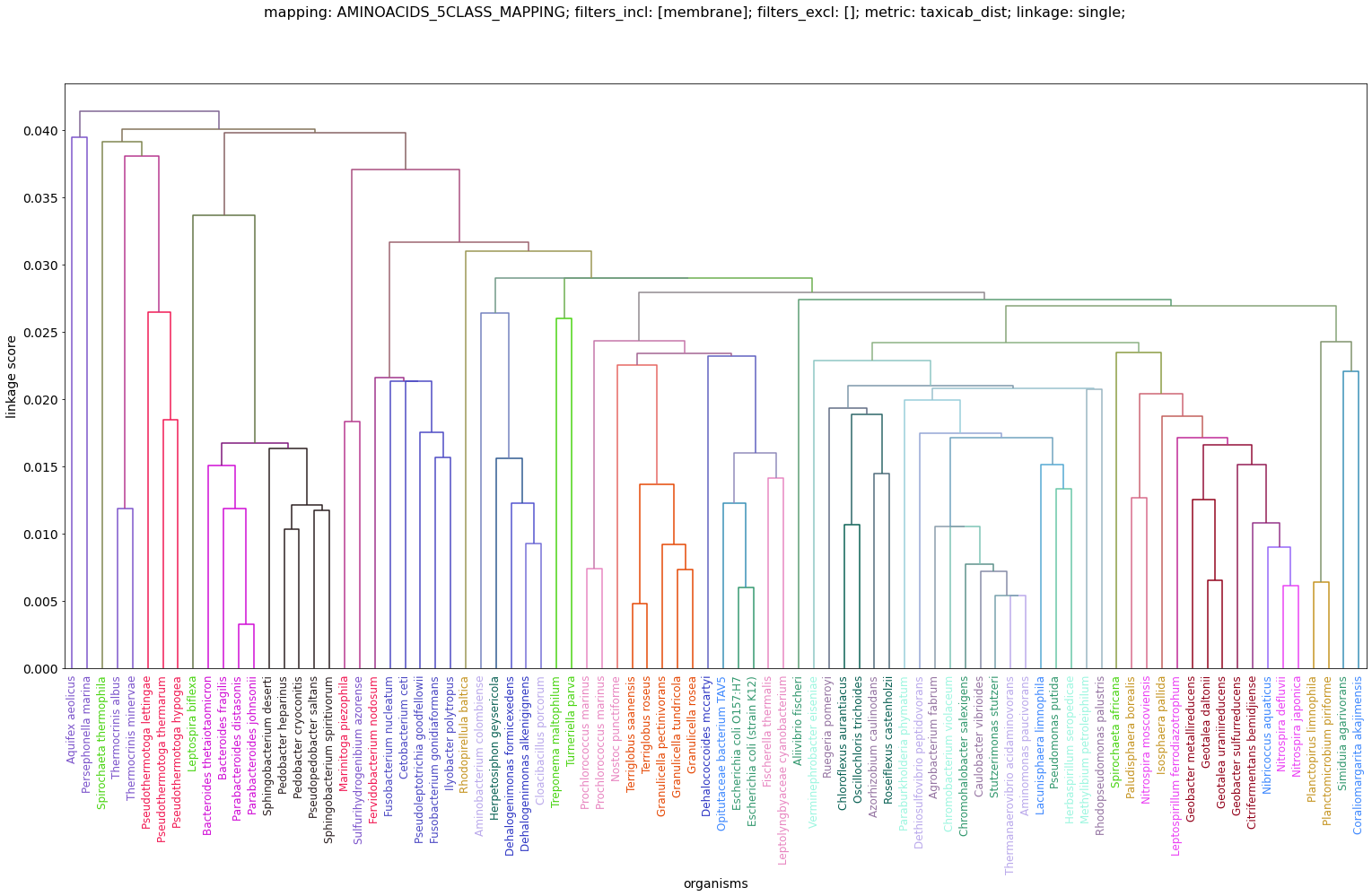

Supplement: Supplementary file 1 [file ijms-27-00109-s001.zip › kmers_supplementary/dendrograms/k1/AMINOACIDS_5CLASS_MAPPING/membrane_/taxicab_dist_single.png]

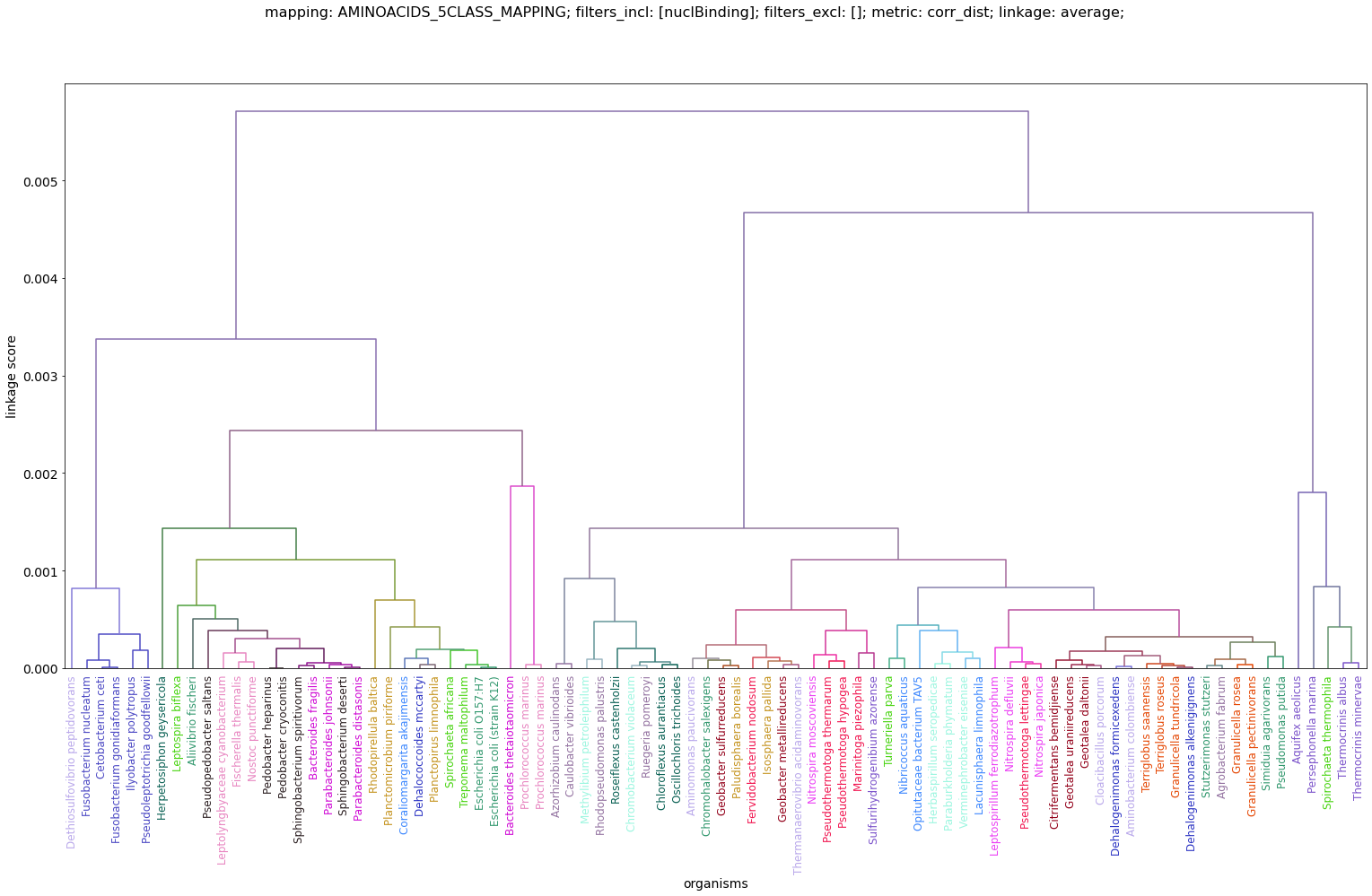

Supplement: Supplementary file 1 [file ijms-27-00109-s001.zip › kmers_supplementary/dendrograms/k1/AMINOACIDS_5CLASS_MAPPING/nuclBinding_/corr_dist_average.png]

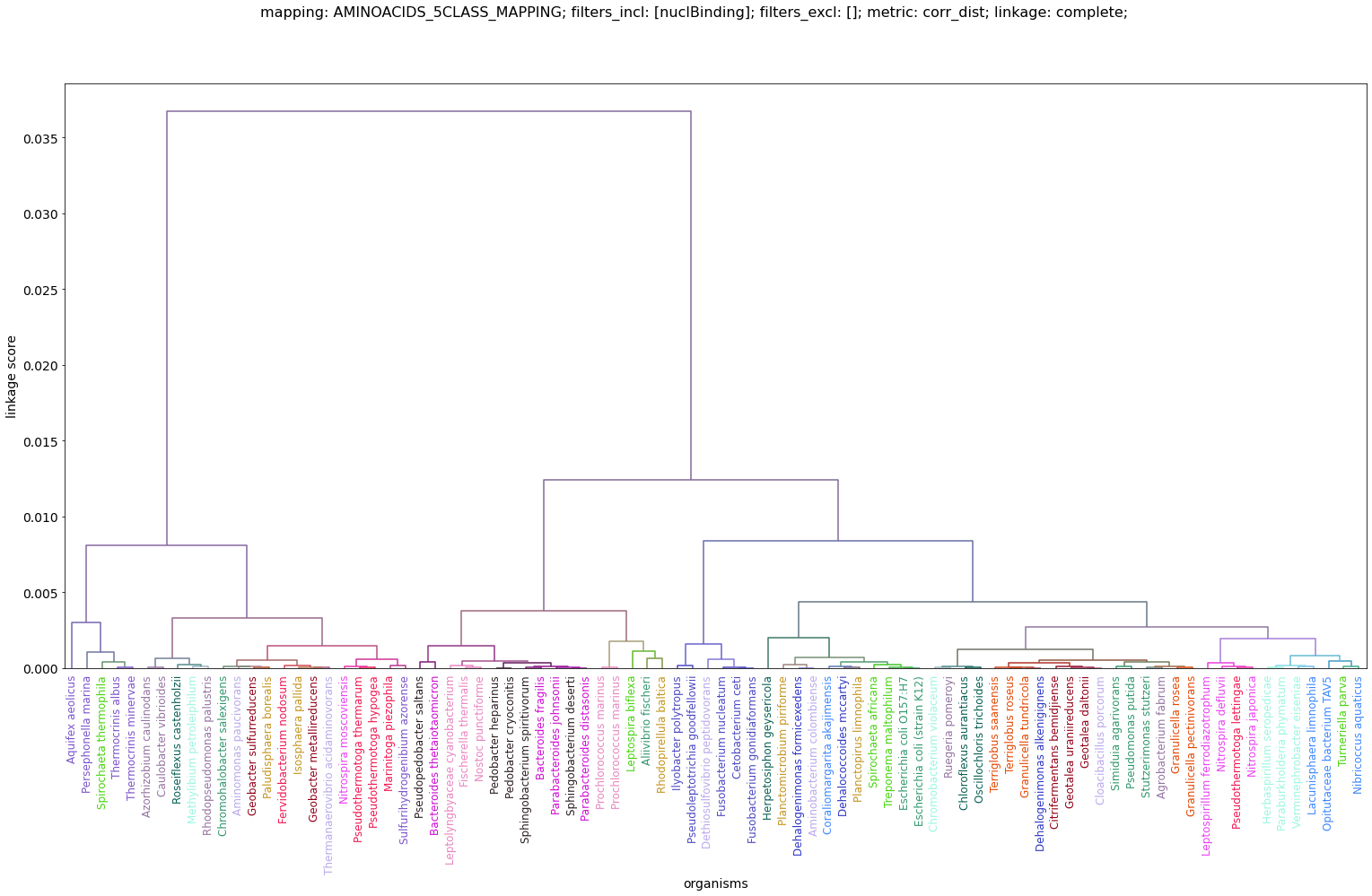

Supplement: Supplementary file 1 [file ijms-27-00109-s001.zip › kmers_supplementary/dendrograms/k1/AMINOACIDS_5CLASS_MAPPING/nuclBinding_/corr_dist_complete.png]

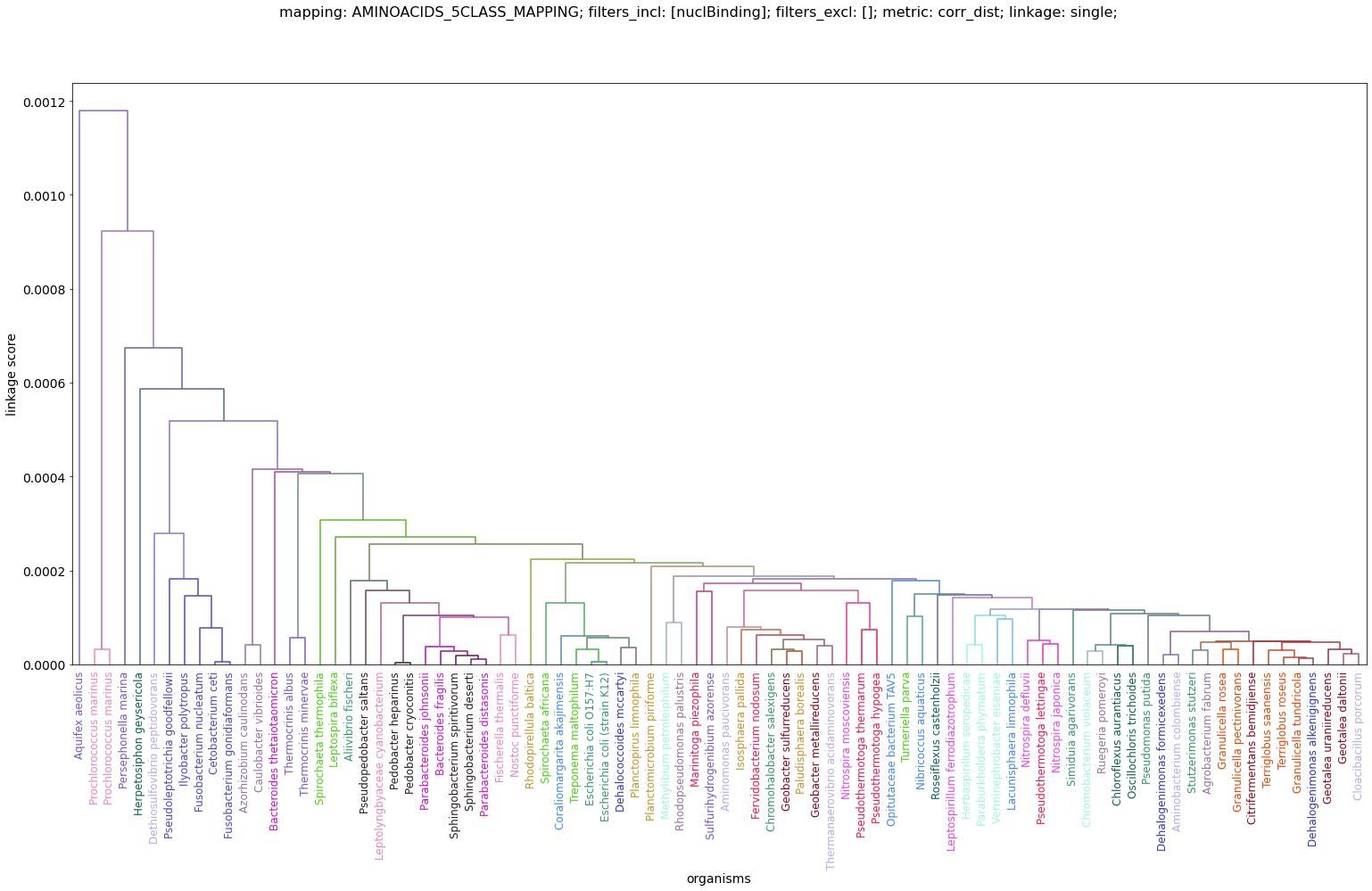

Supplement: Supplementary file 1 [file ijms-27-00109-s001.zip › kmers_supplementary/dendrograms/k1/AMINOACIDS_5CLASS_MAPPING/nuclBinding_/corr_dist_single.png]

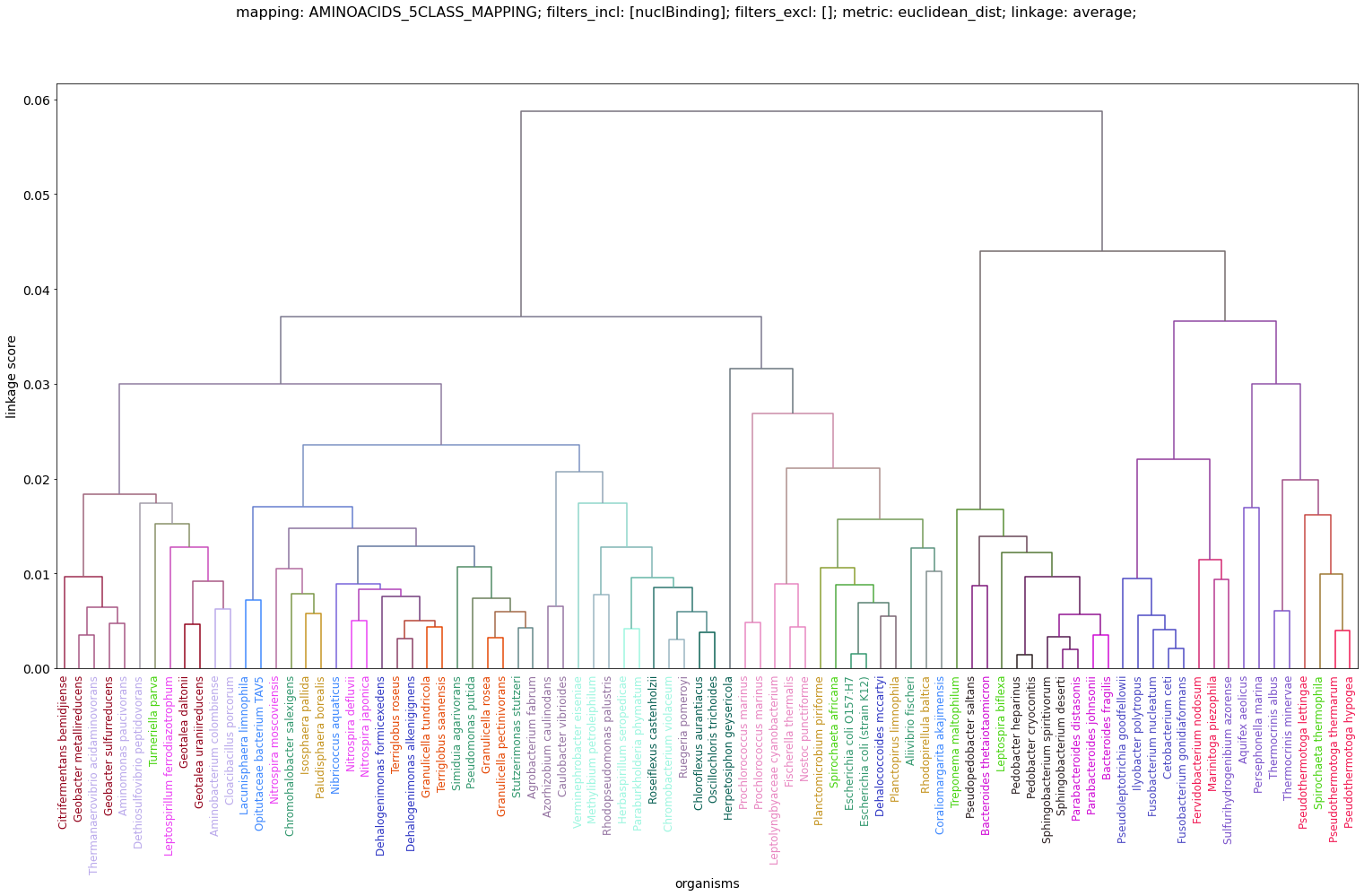

Supplement: Supplementary file 1 [file ijms-27-00109-s001.zip › kmers_supplementary/dendrograms/k1/AMINOACIDS_5CLASS_MAPPING/nuclBinding_/euclidean_dist_average.png]

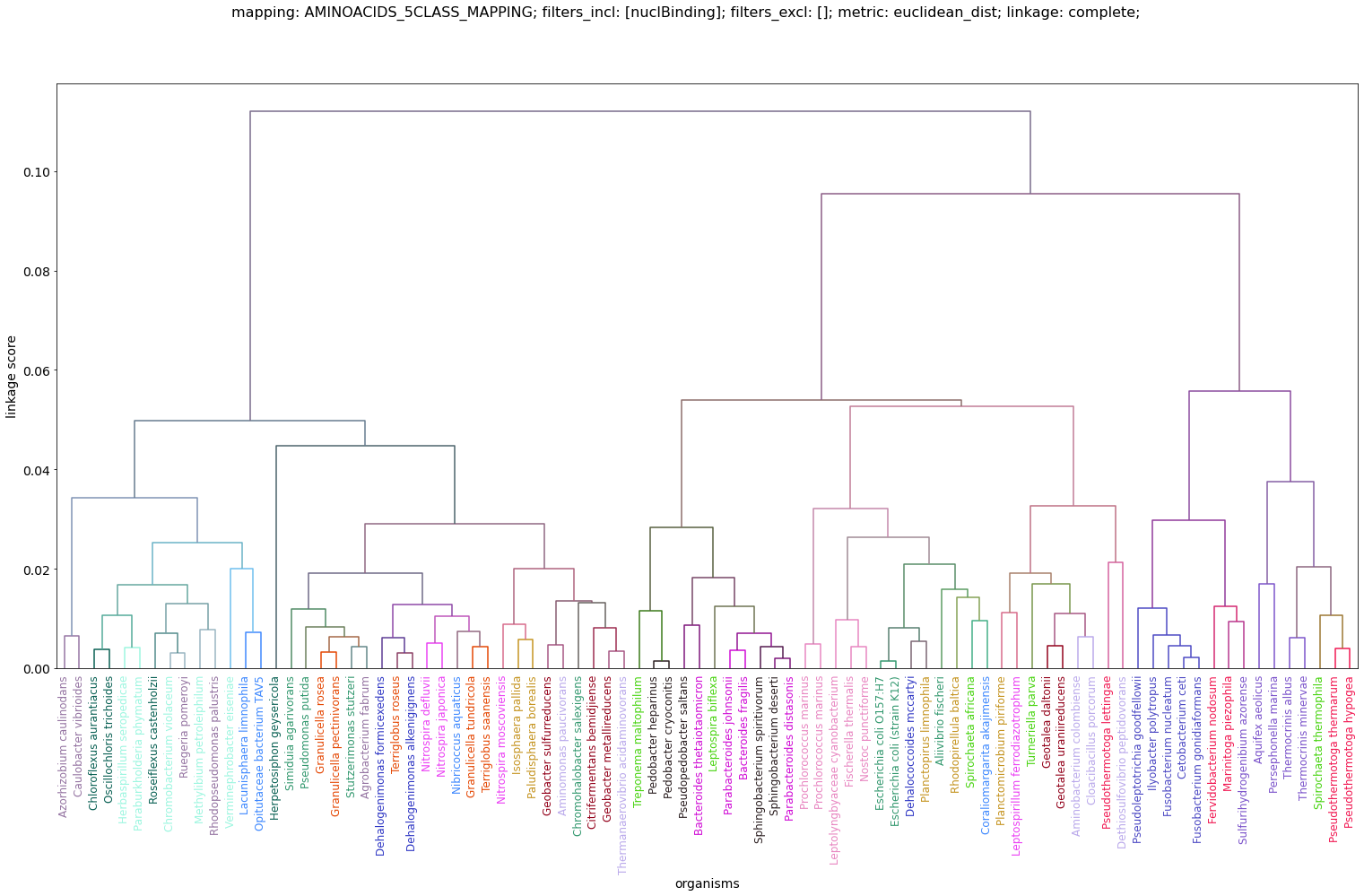

Supplement: Supplementary file 1 [file ijms-27-00109-s001.zip › kmers_supplementary/dendrograms/k1/AMINOACIDS_5CLASS_MAPPING/nuclBinding_/euclidean_dist_complete.png]

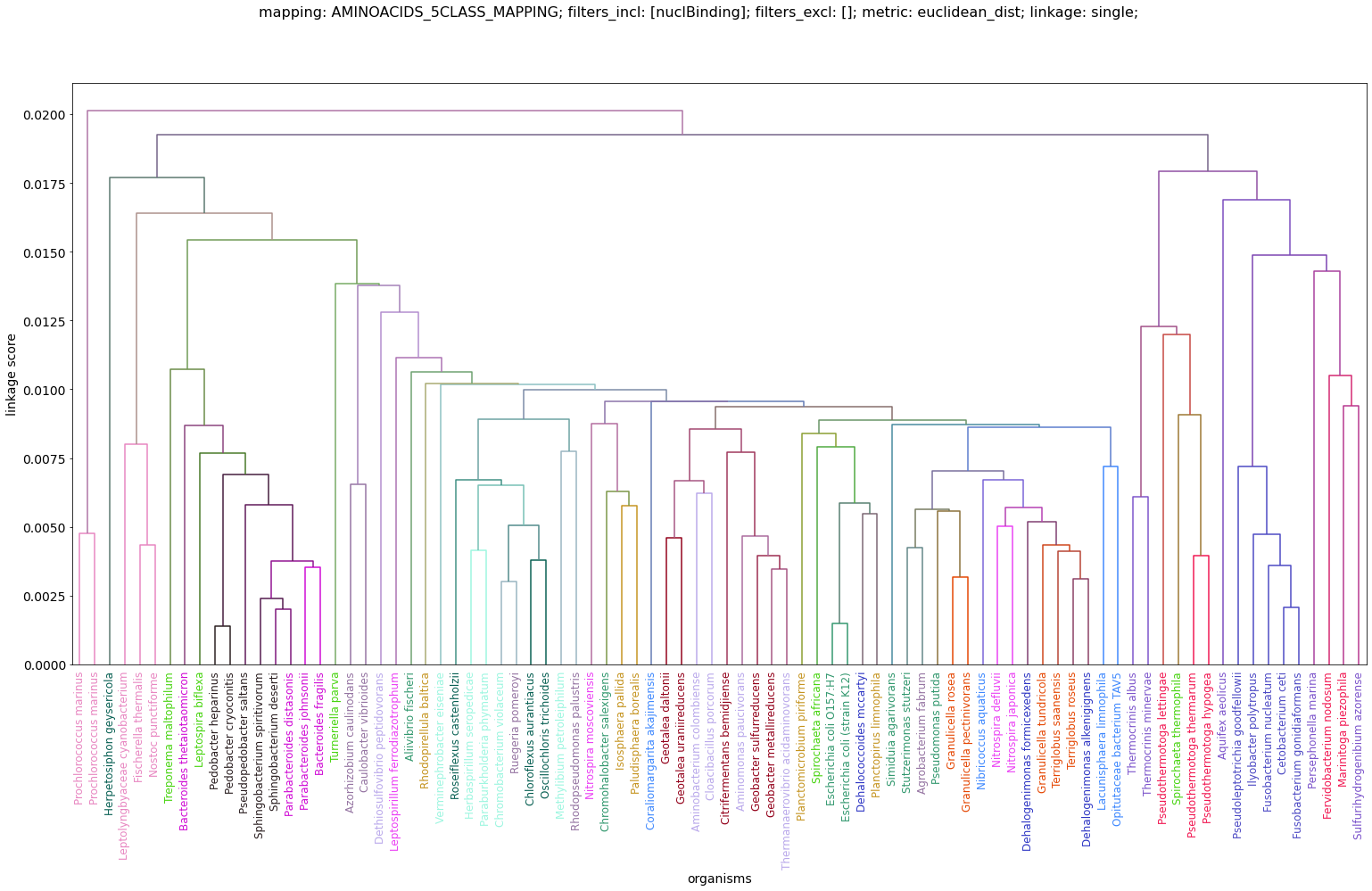

Supplement: Supplementary file 1 [file ijms-27-00109-s001.zip › kmers_supplementary/dendrograms/k1/AMINOACIDS_5CLASS_MAPPING/nuclBinding_/euclidean_dist_single.png]

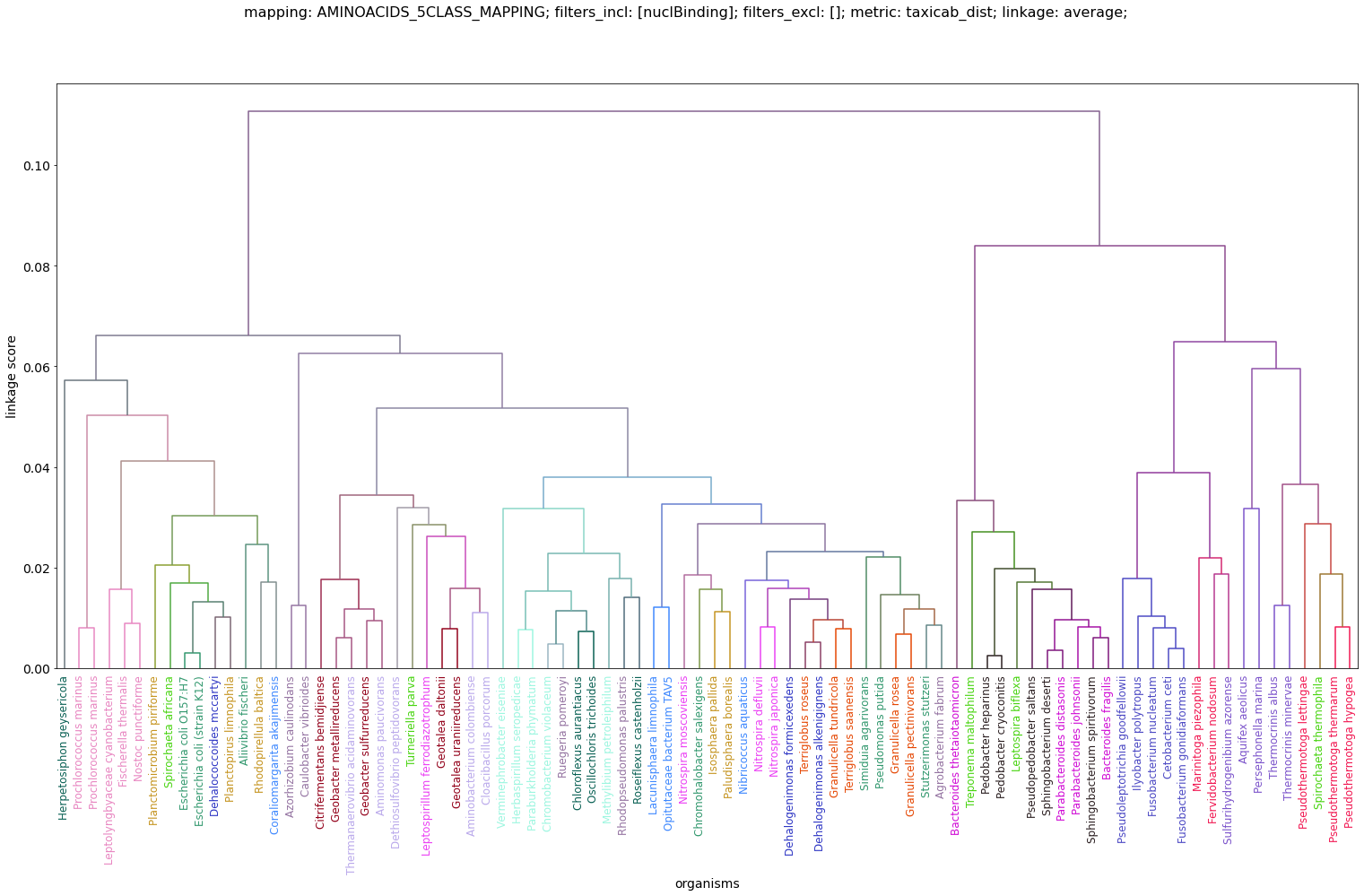

Supplement: Supplementary file 1 [file ijms-27-00109-s001.zip › kmers_supplementary/dendrograms/k1/AMINOACIDS_5CLASS_MAPPING/nuclBinding_/taxicab_dist_average.png]

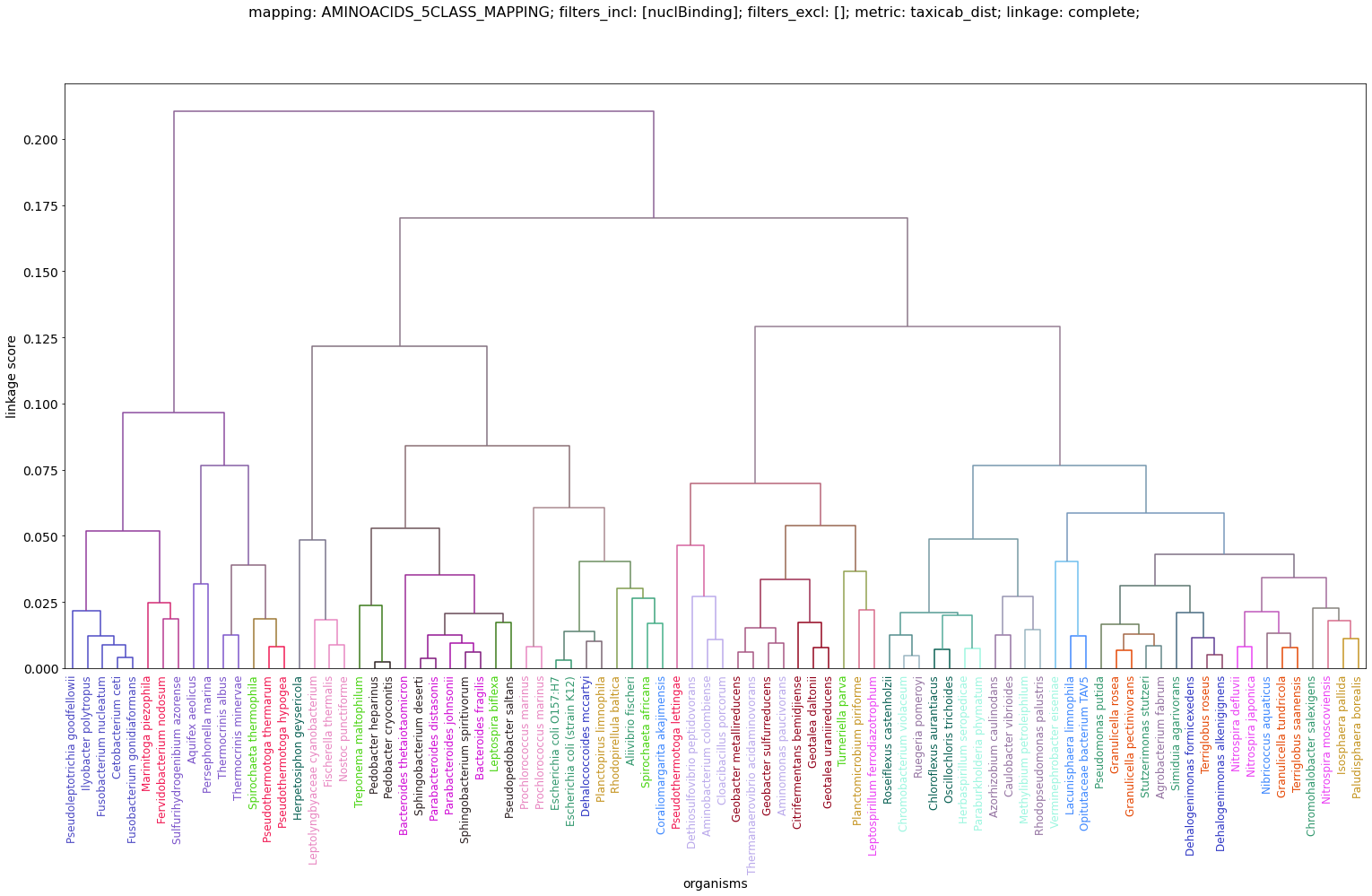

Supplement: Supplementary file 1 [file ijms-27-00109-s001.zip › kmers_supplementary/dendrograms/k1/AMINOACIDS_5CLASS_MAPPING/nuclBinding_/taxicab_dist_complete.png]

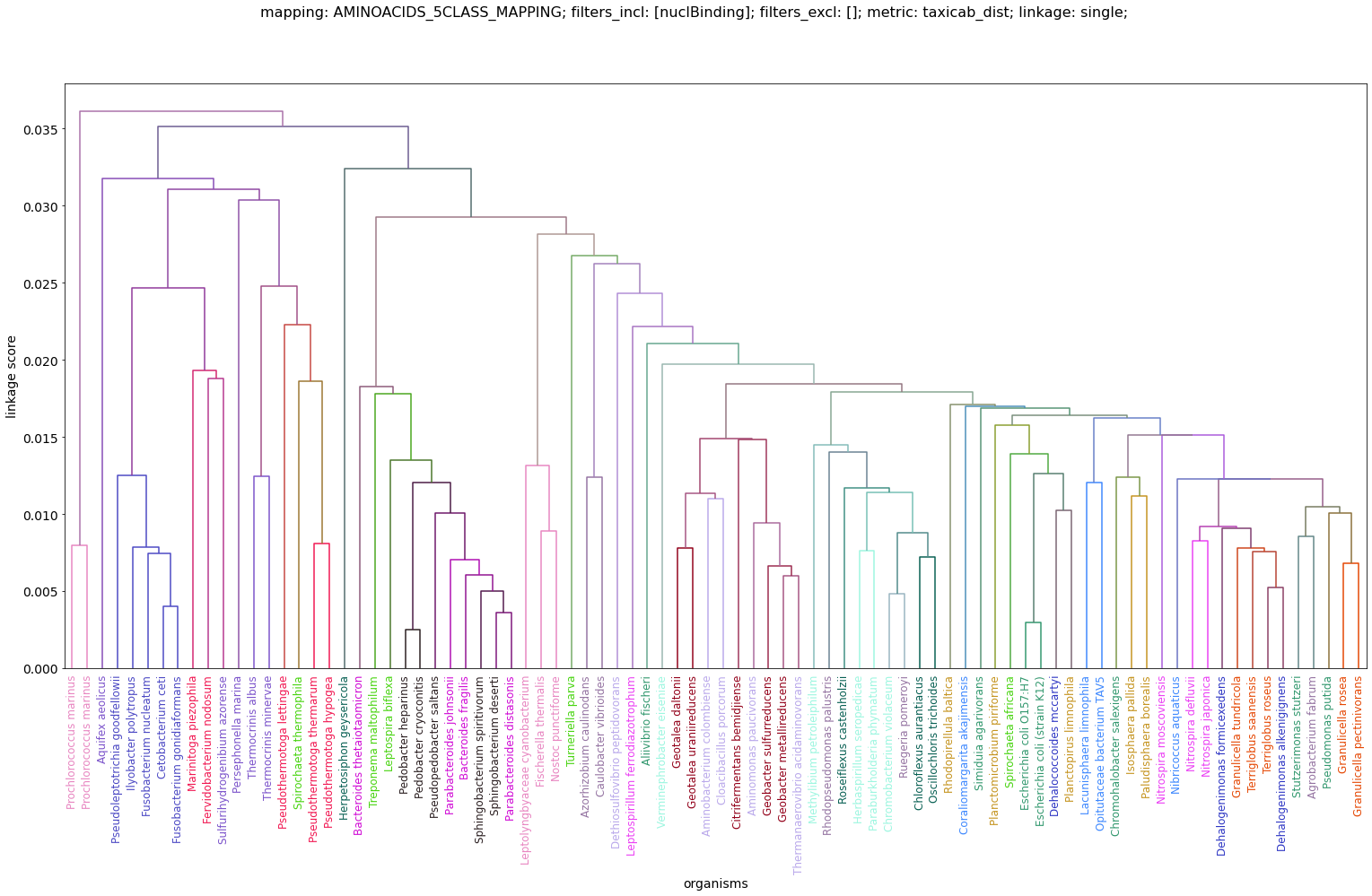

Supplement: Supplementary file 1 [file ijms-27-00109-s001.zip › kmers_supplementary/dendrograms/k1/AMINOACIDS_5CLASS_MAPPING/nuclBinding_/taxicab_dist_single.png]

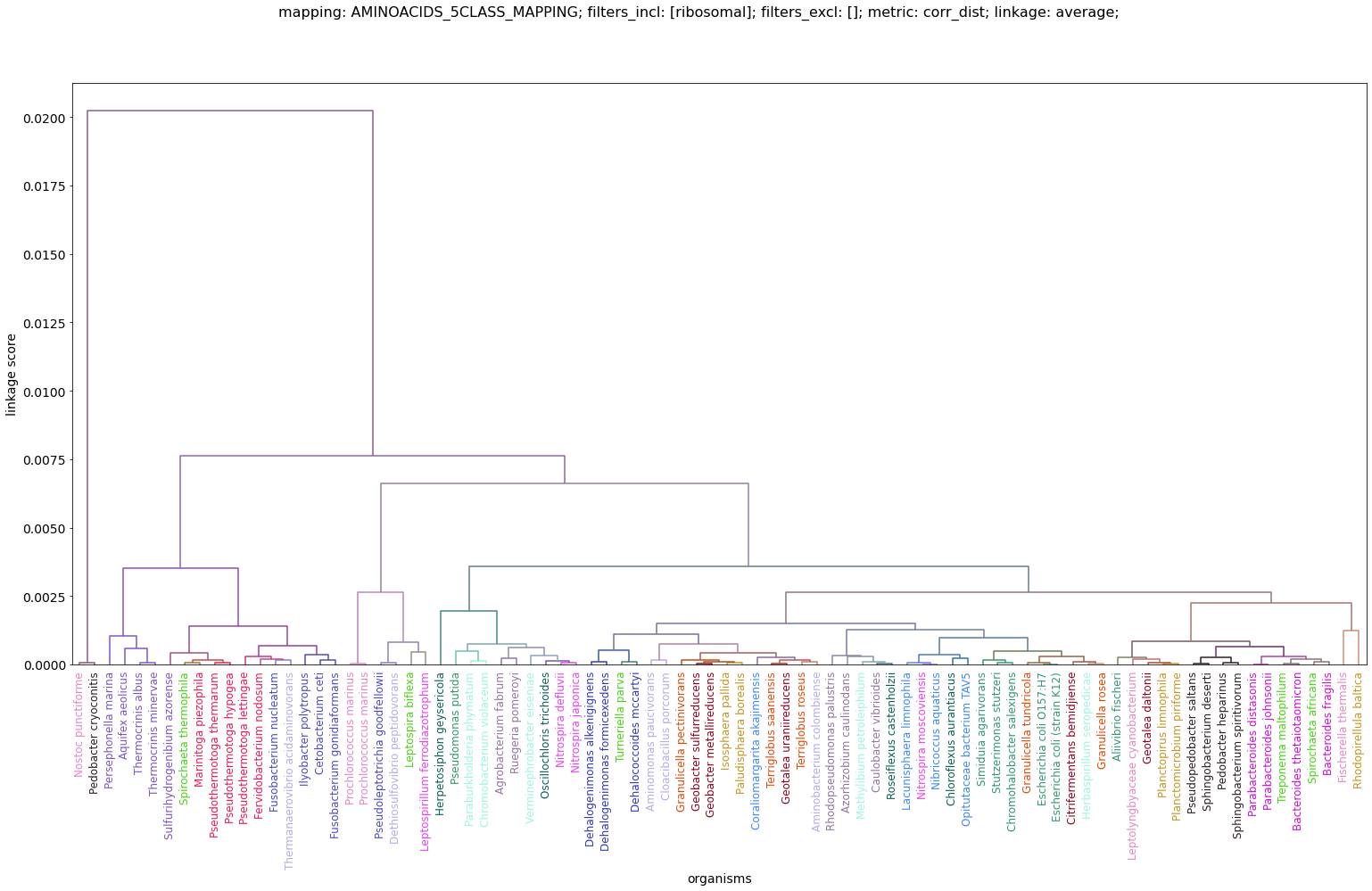

Supplement: Supplementary file 1 [file ijms-27-00109-s001.zip › kmers_supplementary/dendrograms/k1/AMINOACIDS_5CLASS_MAPPING/ribosomal_/corr_dist_average.png]

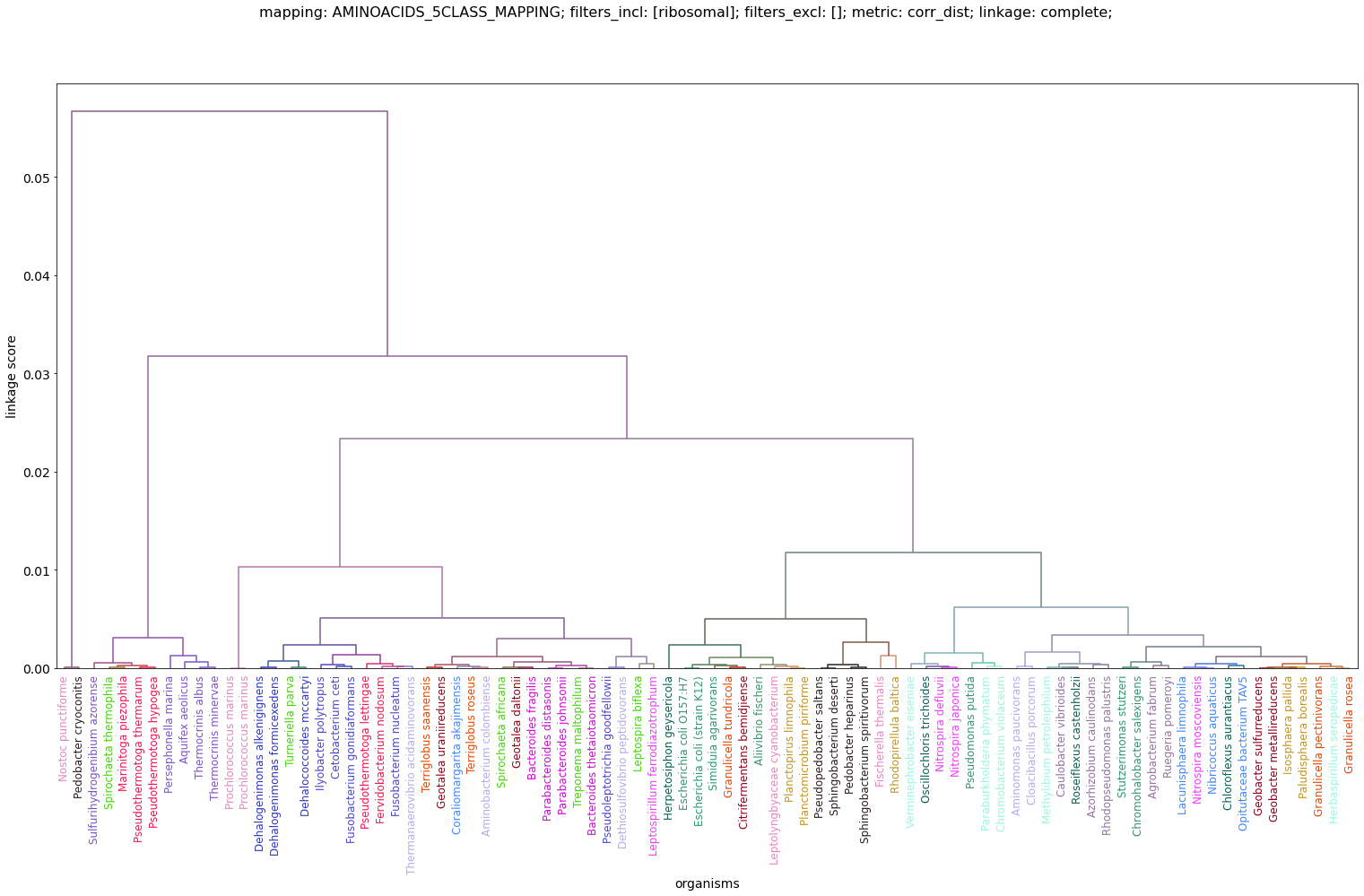

Supplement: Supplementary file 1 [file ijms-27-00109-s001.zip › kmers_supplementary/dendrograms/k1/AMINOACIDS_5CLASS_MAPPING/ribosomal_/corr_dist_complete.png]

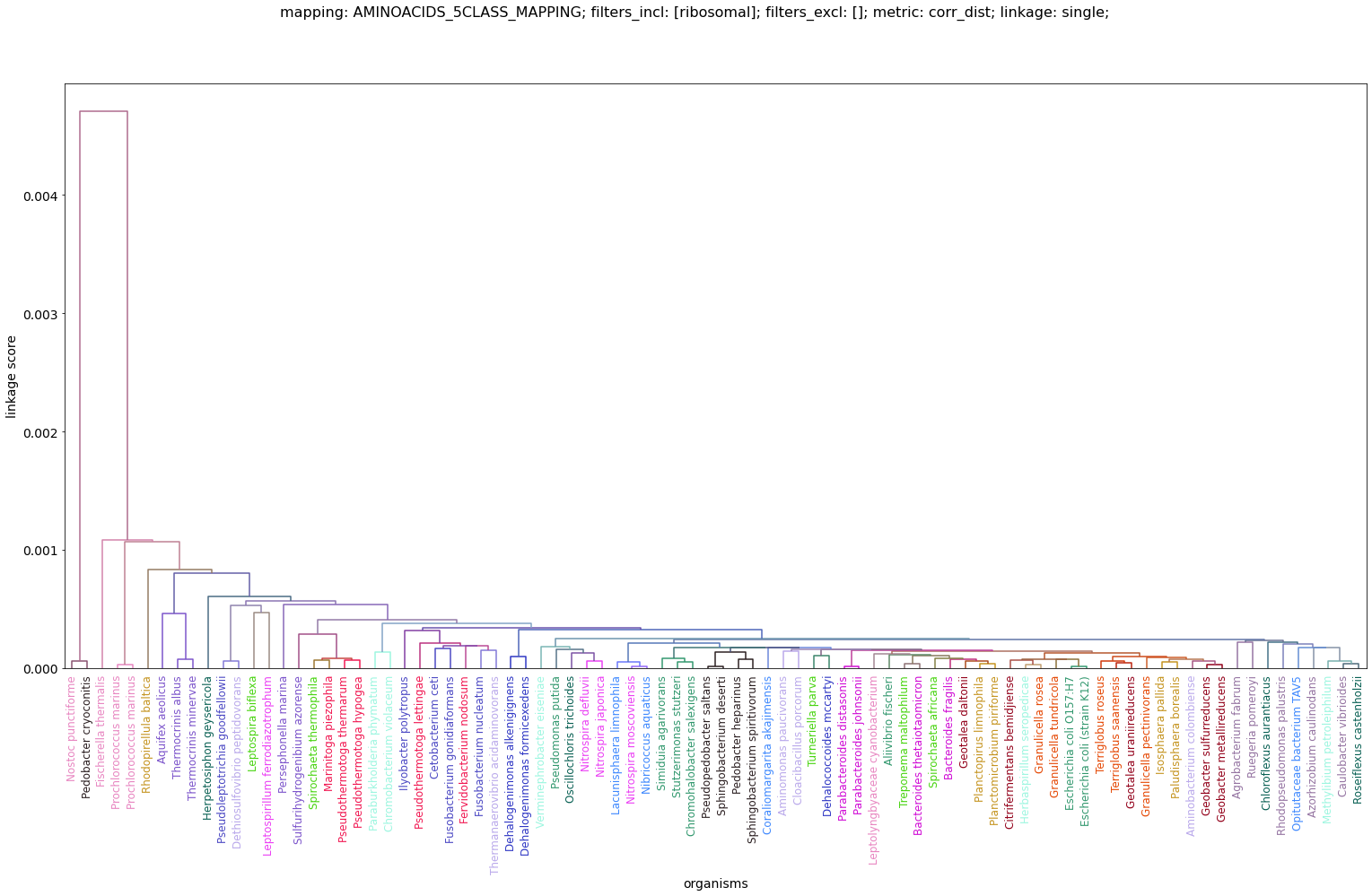

Supplement: Supplementary file 1 [file ijms-27-00109-s001.zip › kmers_supplementary/dendrograms/k1/AMINOACIDS_5CLASS_MAPPING/ribosomal_/corr_dist_single.png]

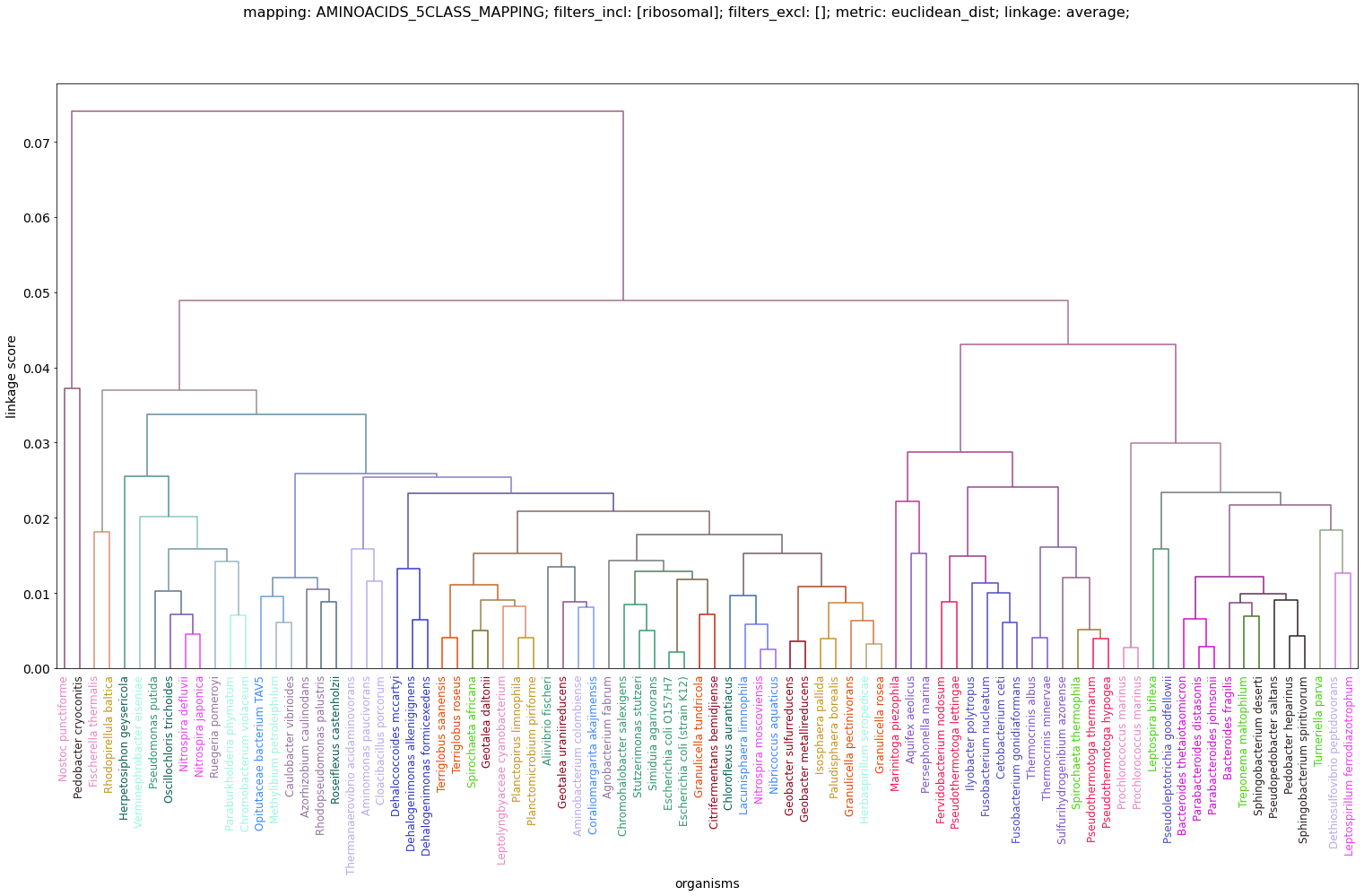

Supplement: Supplementary file 1 [file ijms-27-00109-s001.zip › kmers_supplementary/dendrograms/k1/AMINOACIDS_5CLASS_MAPPING/ribosomal_/euclidean_dist_average.png]

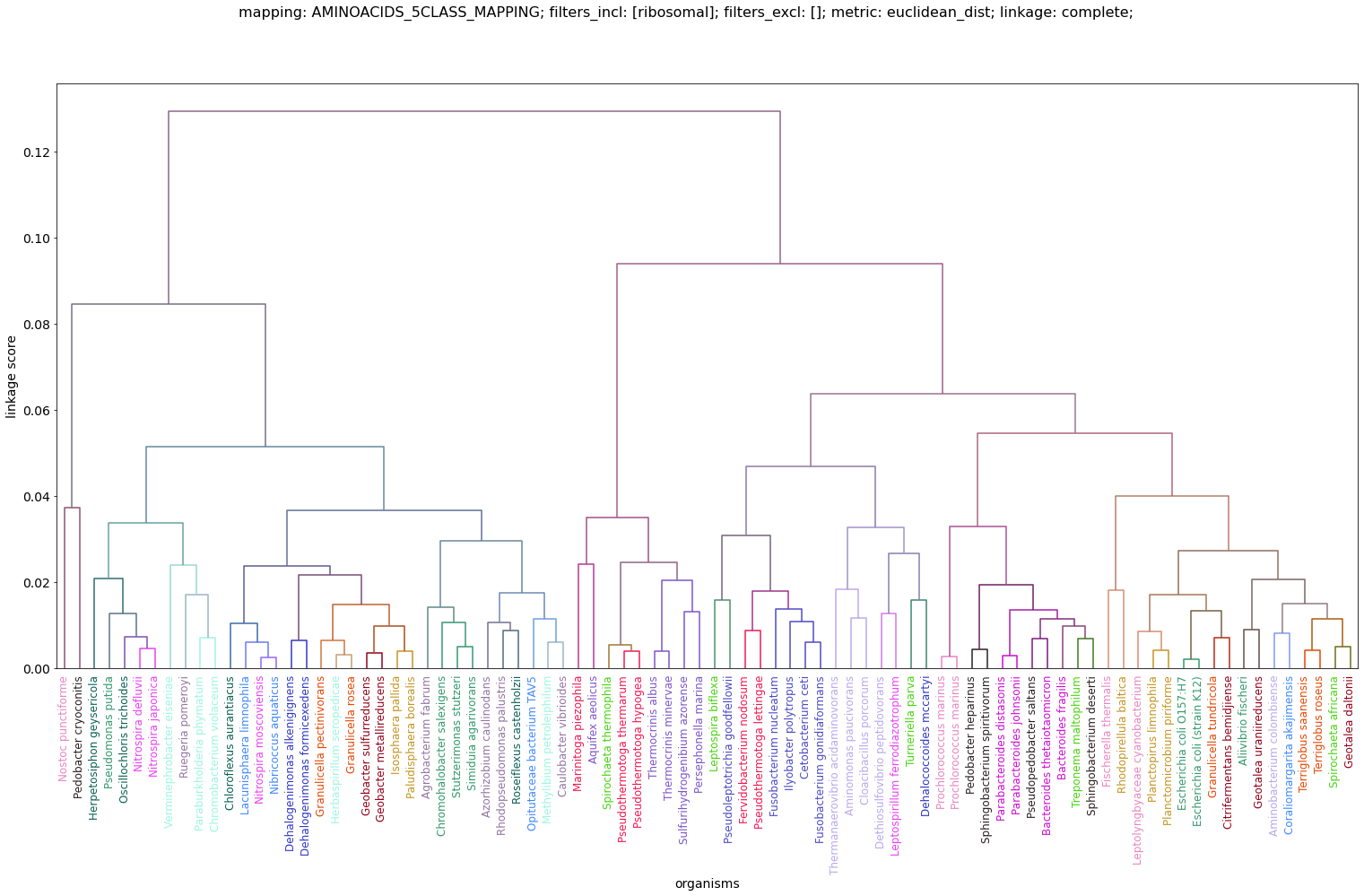

Supplement: Supplementary file 1 [file ijms-27-00109-s001.zip › kmers_supplementary/dendrograms/k1/AMINOACIDS_5CLASS_MAPPING/ribosomal_/euclidean_dist_complete.png]

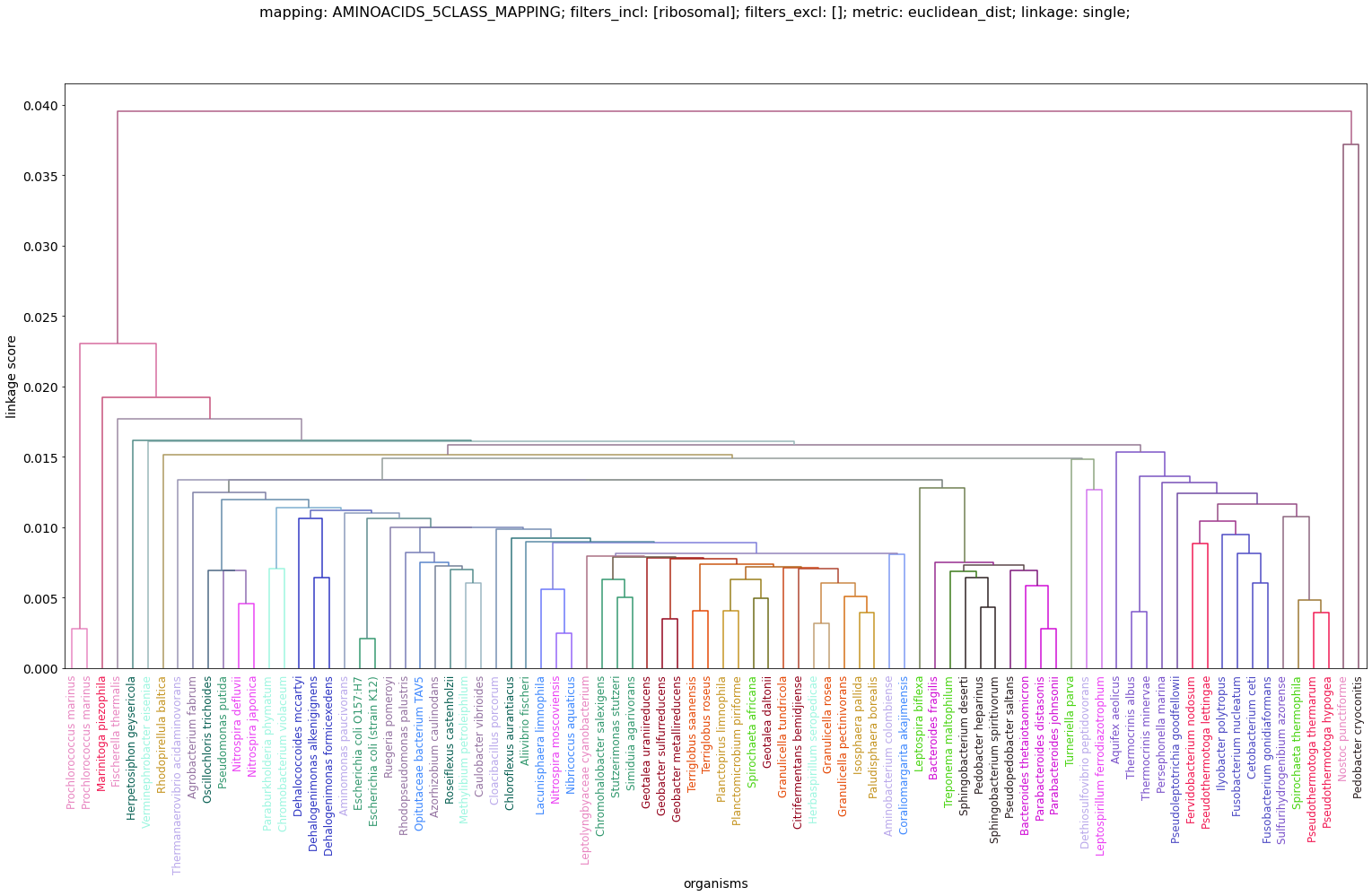

Supplement: Supplementary file 1 [file ijms-27-00109-s001.zip › kmers_supplementary/dendrograms/k1/AMINOACIDS_5CLASS_MAPPING/ribosomal_/euclidean_dist_single.png]

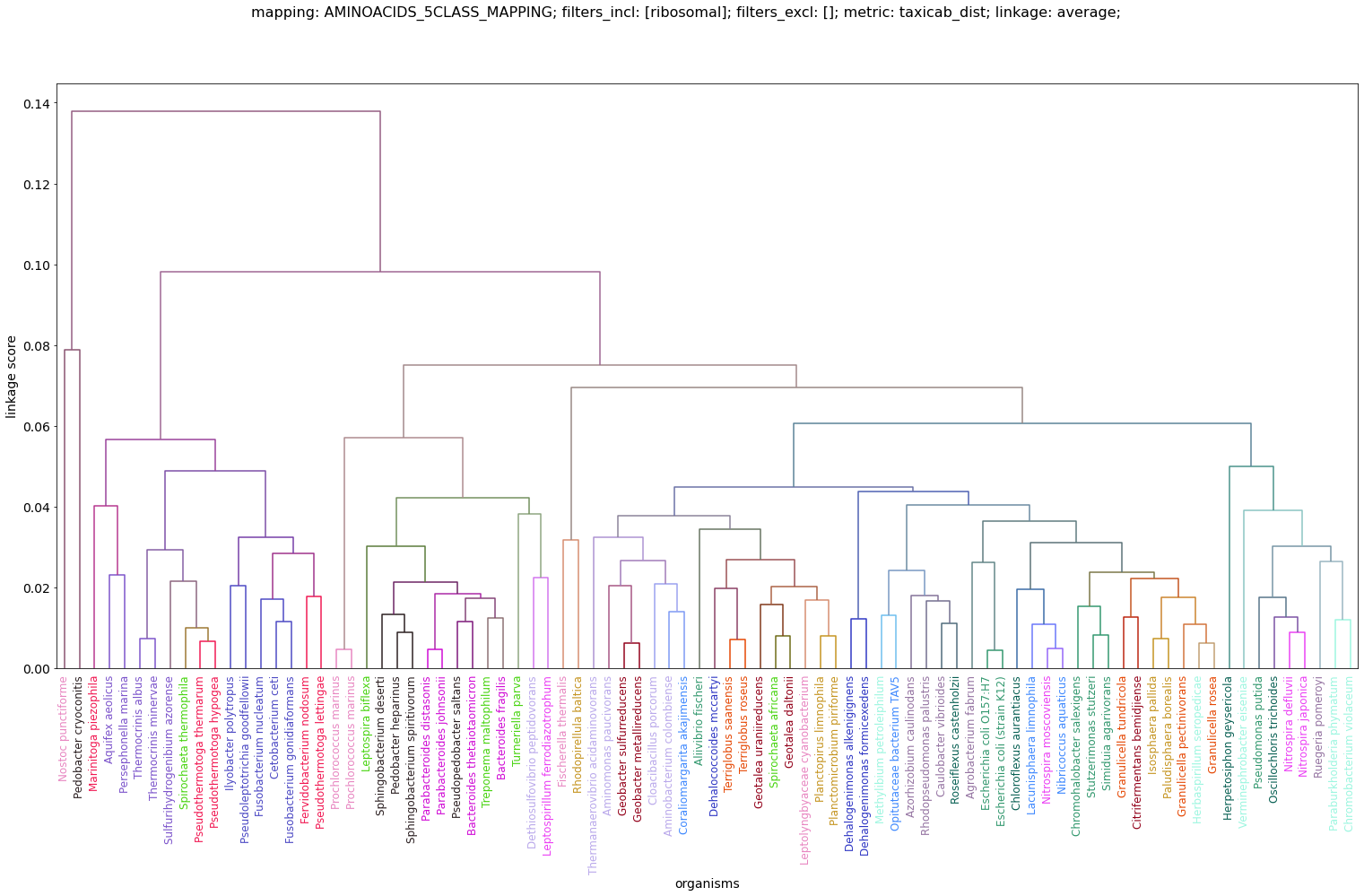

Supplement: Supplementary file 1 [file ijms-27-00109-s001.zip › kmers_supplementary/dendrograms/k1/AMINOACIDS_5CLASS_MAPPING/ribosomal_/taxicab_dist_average.png]

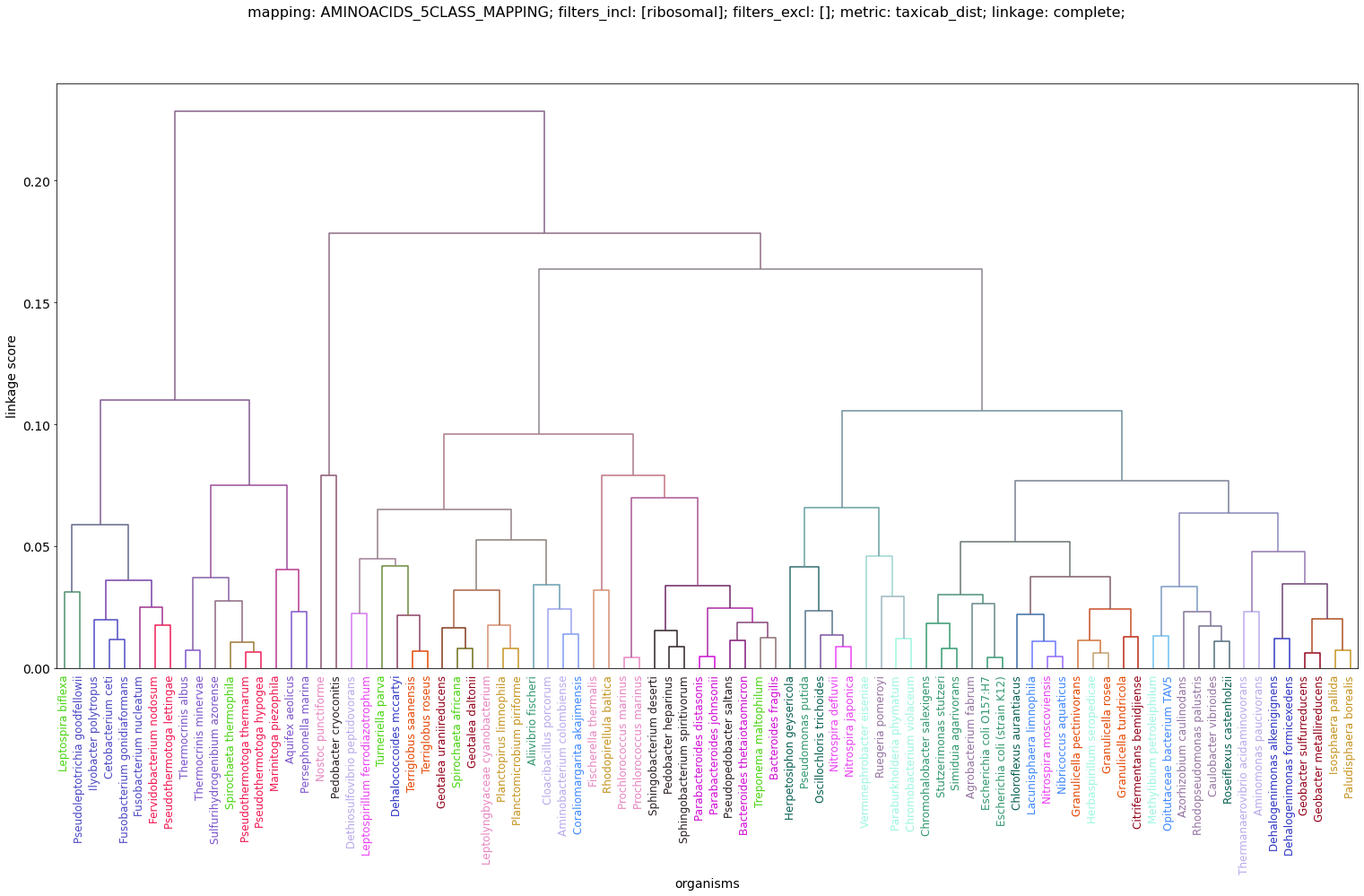

Supplement: Supplementary file 1 [file ijms-27-00109-s001.zip › kmers_supplementary/dendrograms/k1/AMINOACIDS_5CLASS_MAPPING/ribosomal_/taxicab_dist_complete.png]

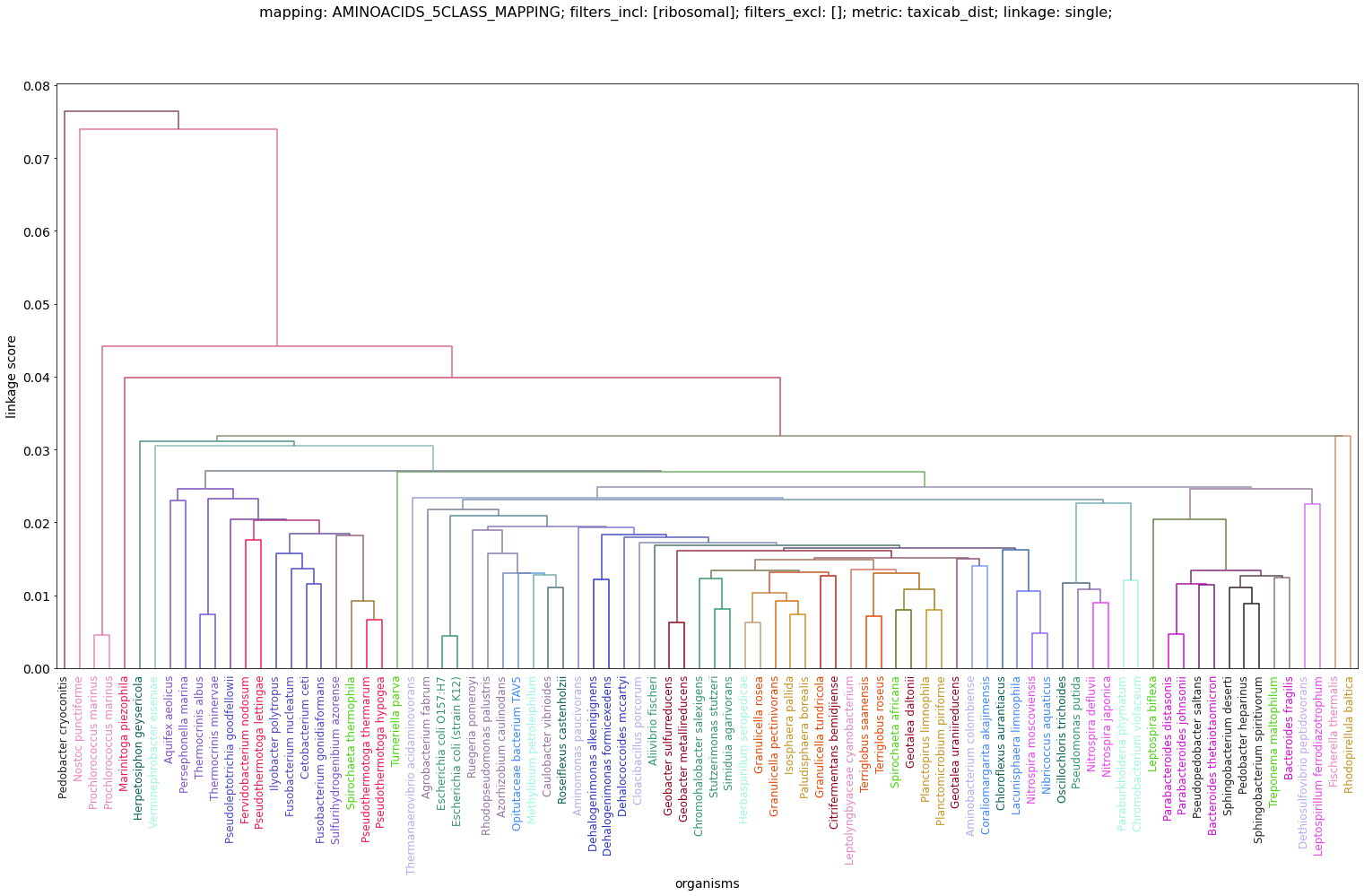

Supplement: Supplementary file 1 [file ijms-27-00109-s001.zip › kmers_supplementary/dendrograms/k1/AMINOACIDS_5CLASS_MAPPING/ribosomal_/taxicab_dist_single.png]

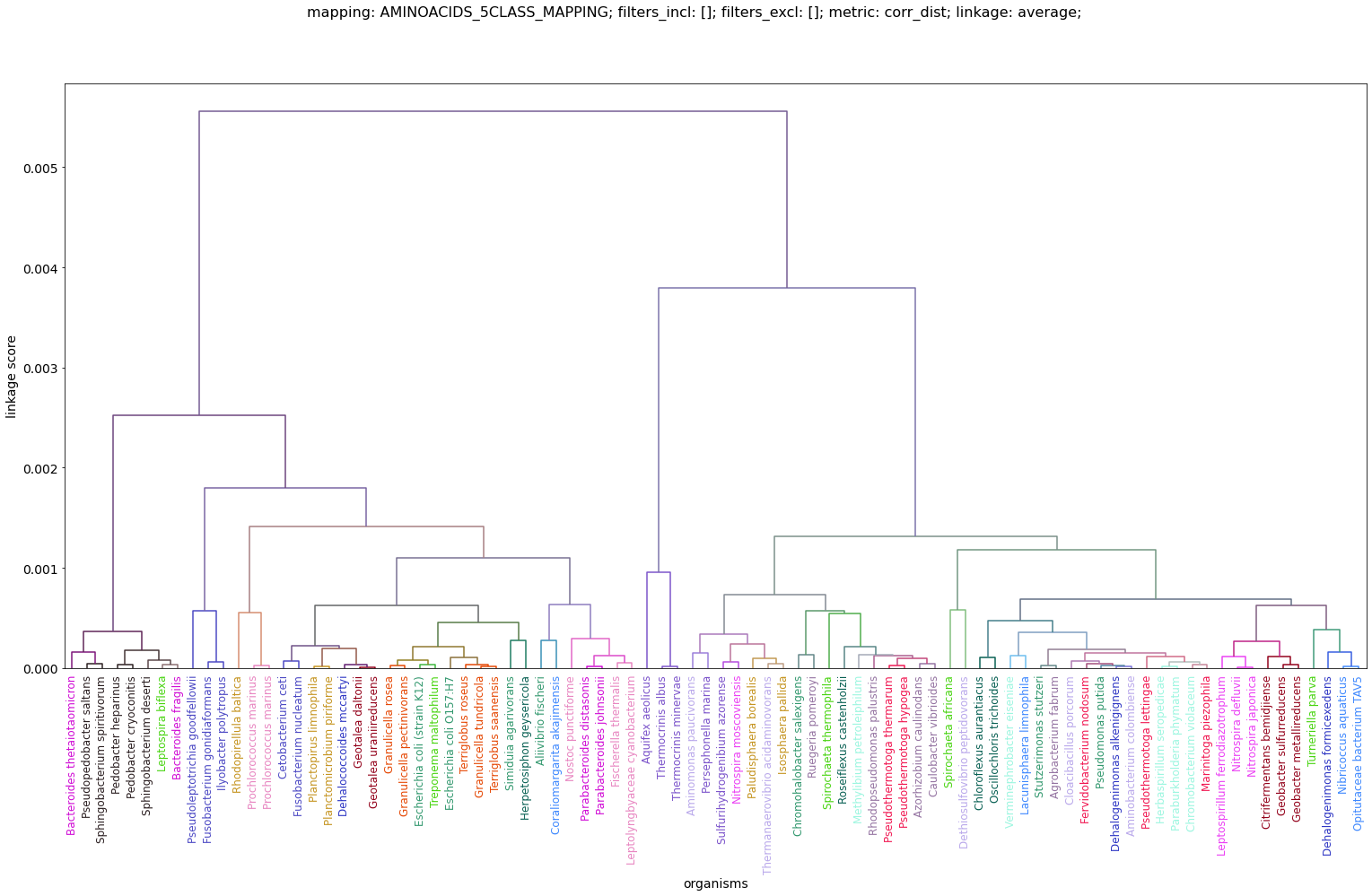

Supplement: Supplementary file 1 [file ijms-27-00109-s001.zip › kmers_supplementary/dendrograms/k1/AMINOACIDS_5CLASS_MAPPING/_/corr_dist_average.png]

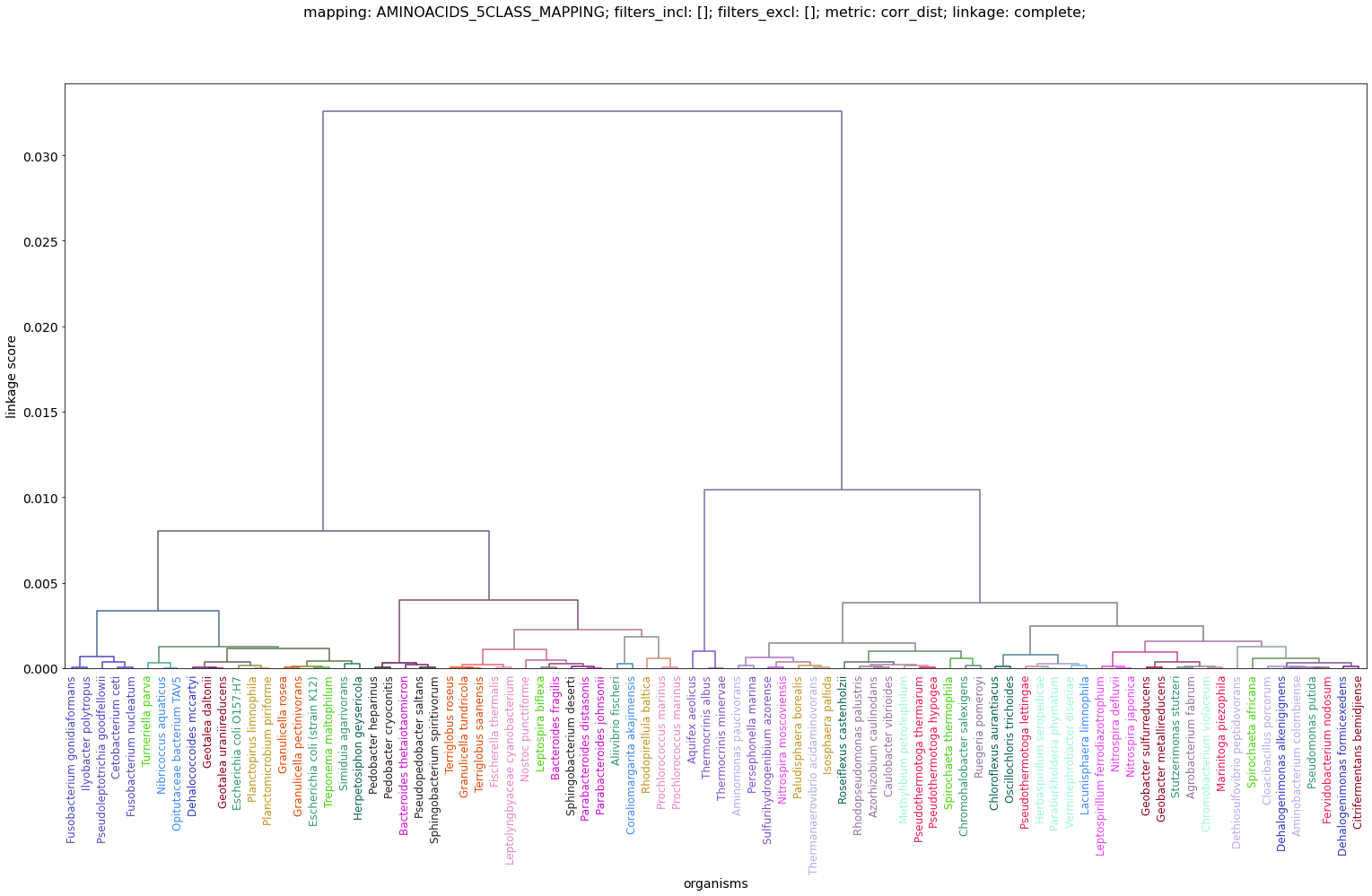

Supplement: Supplementary file 1 [file ijms-27-00109-s001.zip › kmers_supplementary/dendrograms/k1/AMINOACIDS_5CLASS_MAPPING/_/corr_dist_complete.png]

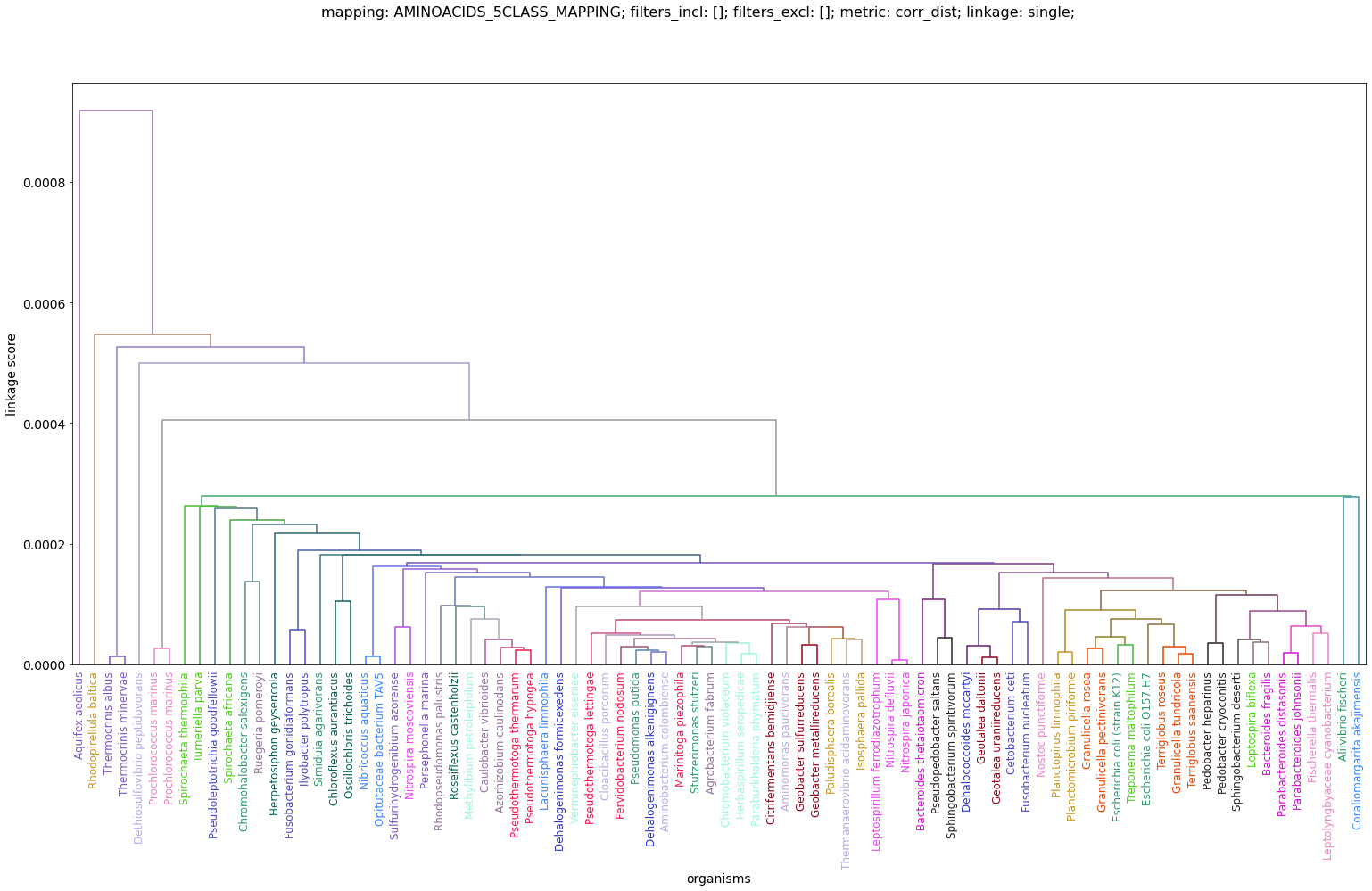

Supplement: Supplementary file 1 [file ijms-27-00109-s001.zip › kmers_supplementary/dendrograms/k1/AMINOACIDS_5CLASS_MAPPING/_/corr_dist_single.png]

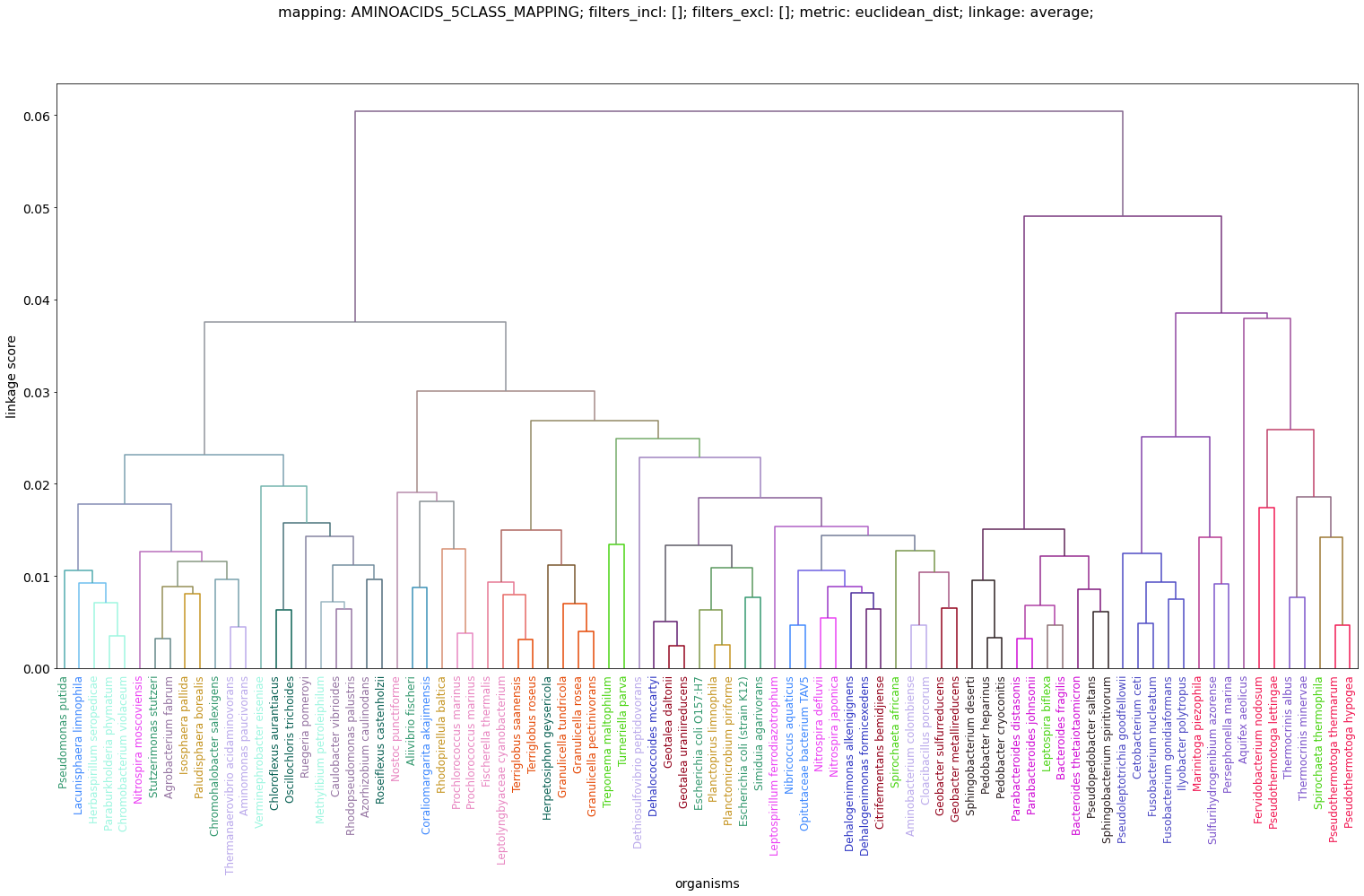

Supplement: Supplementary file 1 [file ijms-27-00109-s001.zip › kmers_supplementary/dendrograms/k1/AMINOACIDS_5CLASS_MAPPING/_/euclidean_dist_average.png]

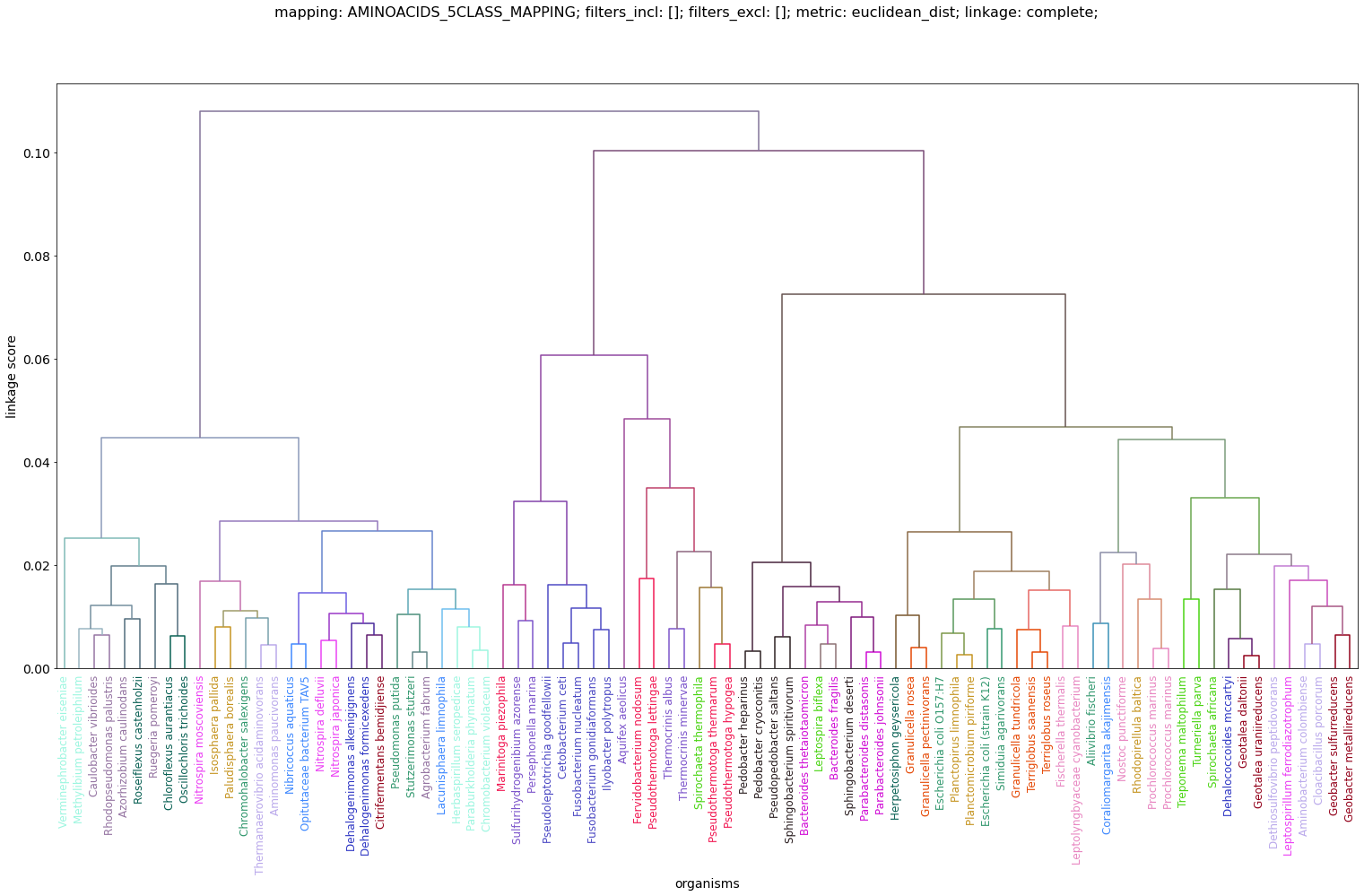

Supplement: Supplementary file 1 [file ijms-27-00109-s001.zip › kmers_supplementary/dendrograms/k1/AMINOACIDS_5CLASS_MAPPING/_/euclidean_dist_complete.png]

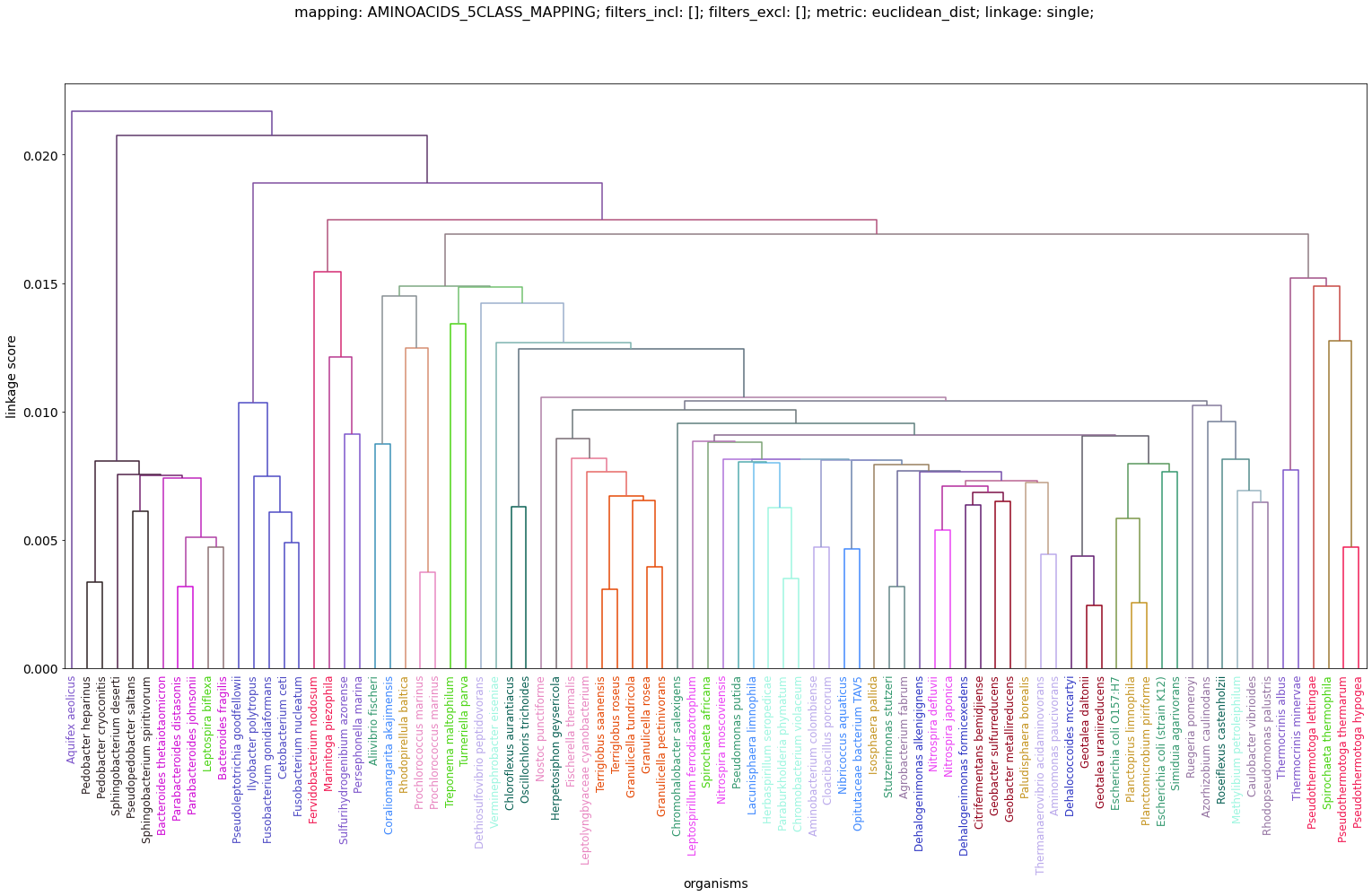

Supplement: Supplementary file 1 [file ijms-27-00109-s001.zip › kmers_supplementary/dendrograms/k1/AMINOACIDS_5CLASS_MAPPING/_/euclidean_dist_single.png]

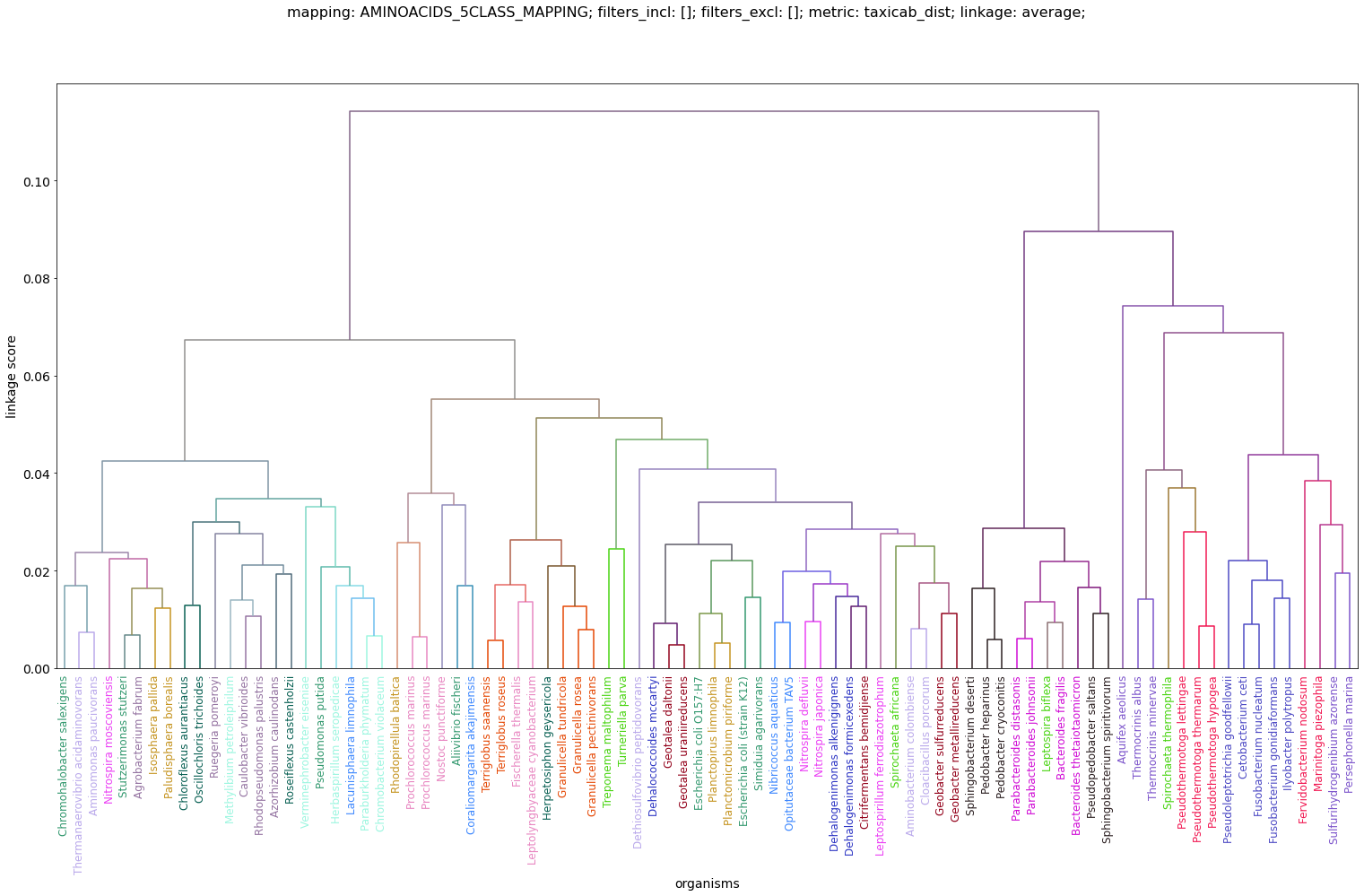

Supplement: Supplementary file 1 [file ijms-27-00109-s001.zip › kmers_supplementary/dendrograms/k1/AMINOACIDS_5CLASS_MAPPING/_/taxicab_dist_average.png]

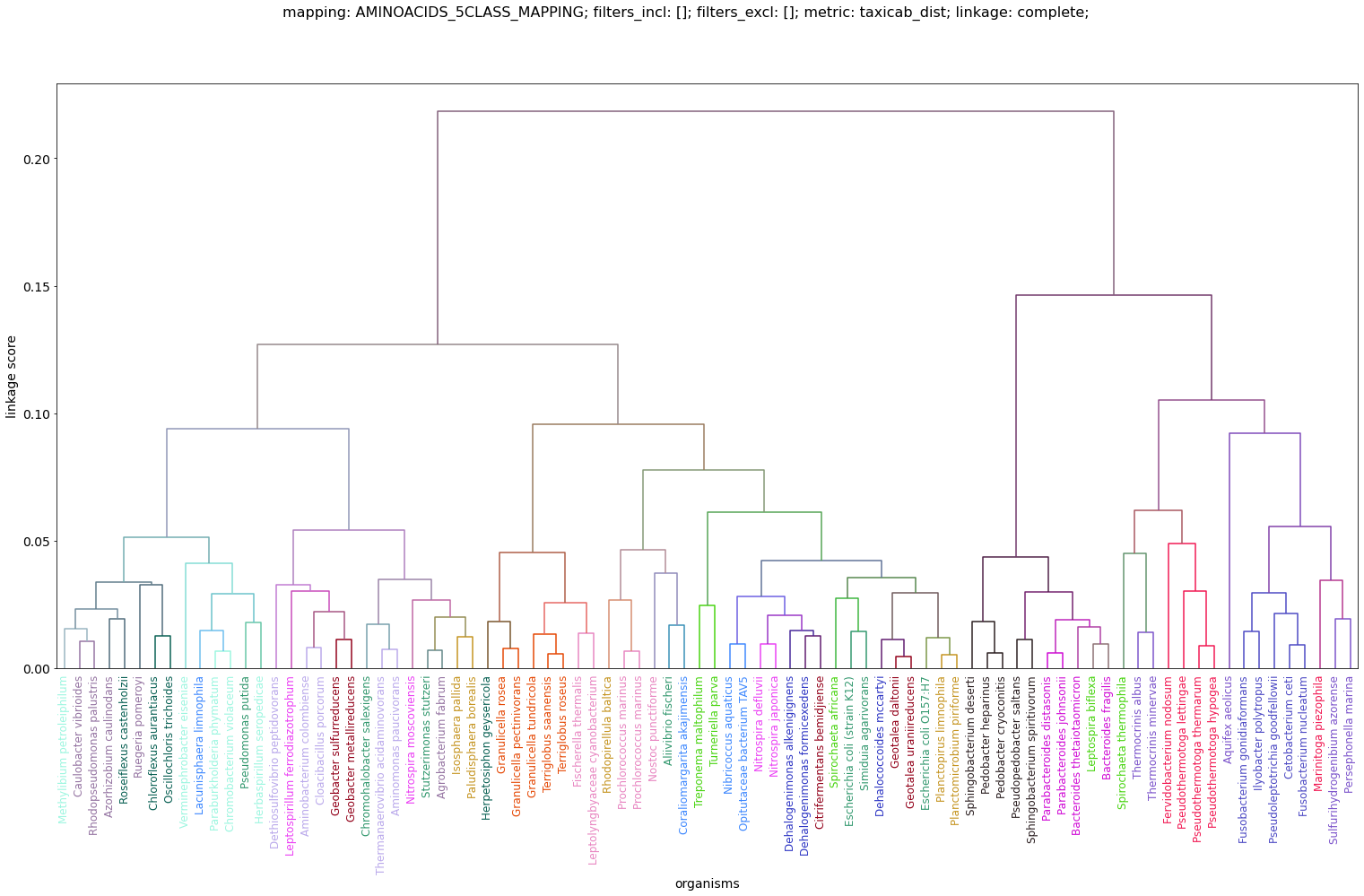

Supplement: Supplementary file 1 [file ijms-27-00109-s001.zip › kmers_supplementary/dendrograms/k1/AMINOACIDS_5CLASS_MAPPING/_/taxicab_dist_complete.png]

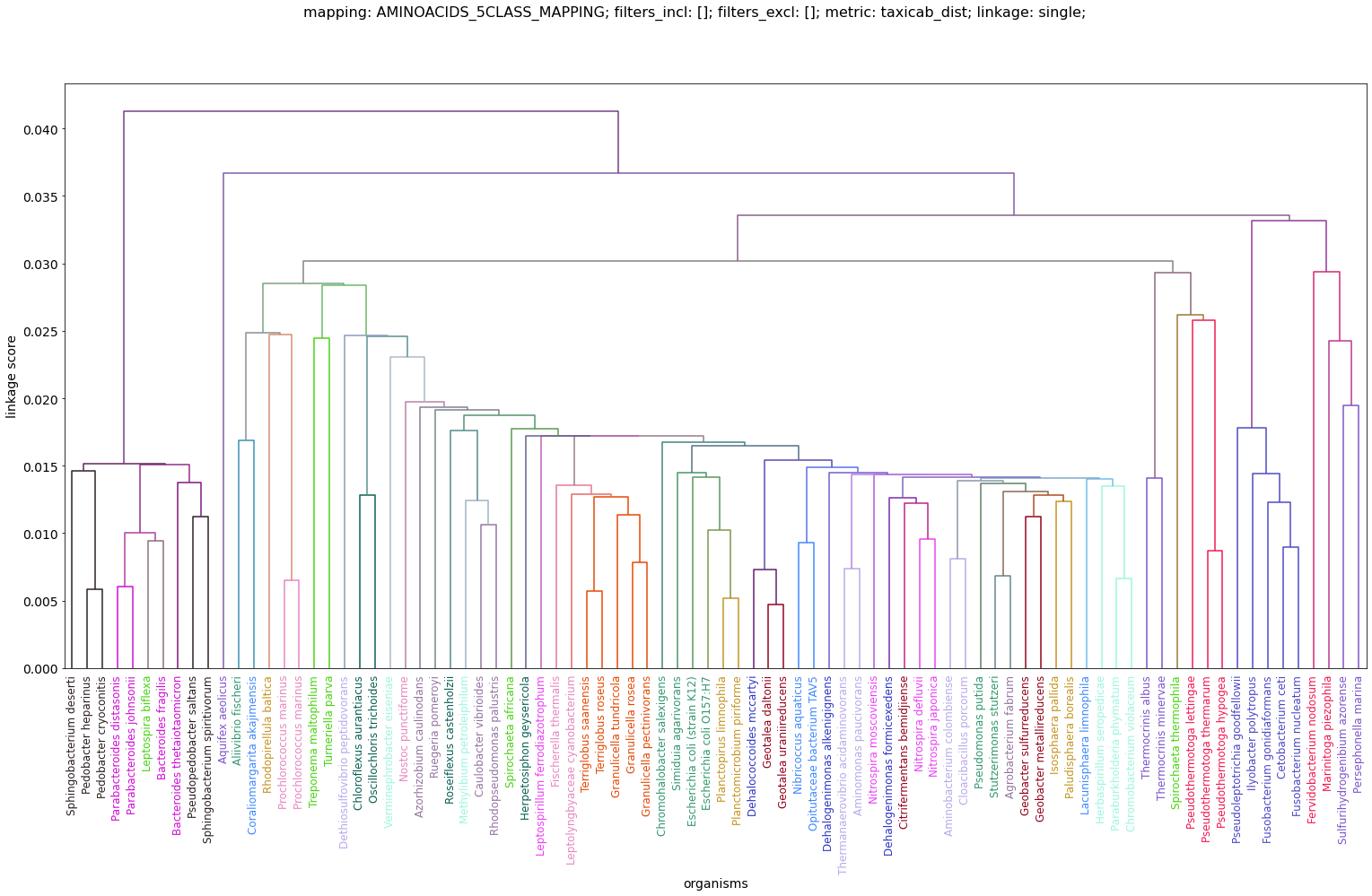

Supplement: Supplementary file 1 [file ijms-27-00109-s001.zip › kmers_supplementary/dendrograms/k1/AMINOACIDS_5CLASS_MAPPING/_/taxicab_dist_single.png]

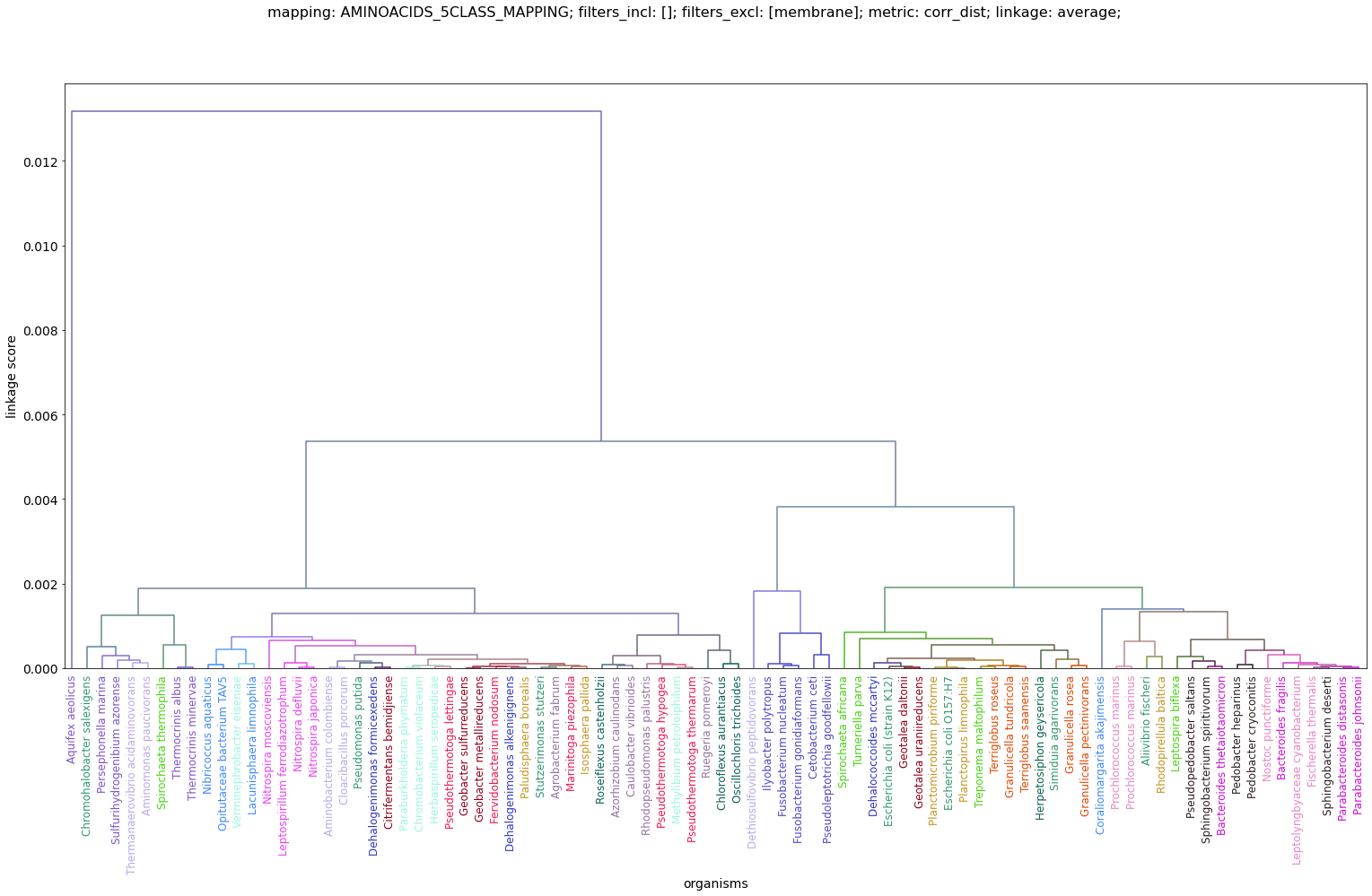

Supplement: Supplementary file 1 [file ijms-27-00109-s001.zip › kmers_supplementary/dendrograms/k1/AMINOACIDS_5CLASS_MAPPING/_membrane/corr_dist_average.png]

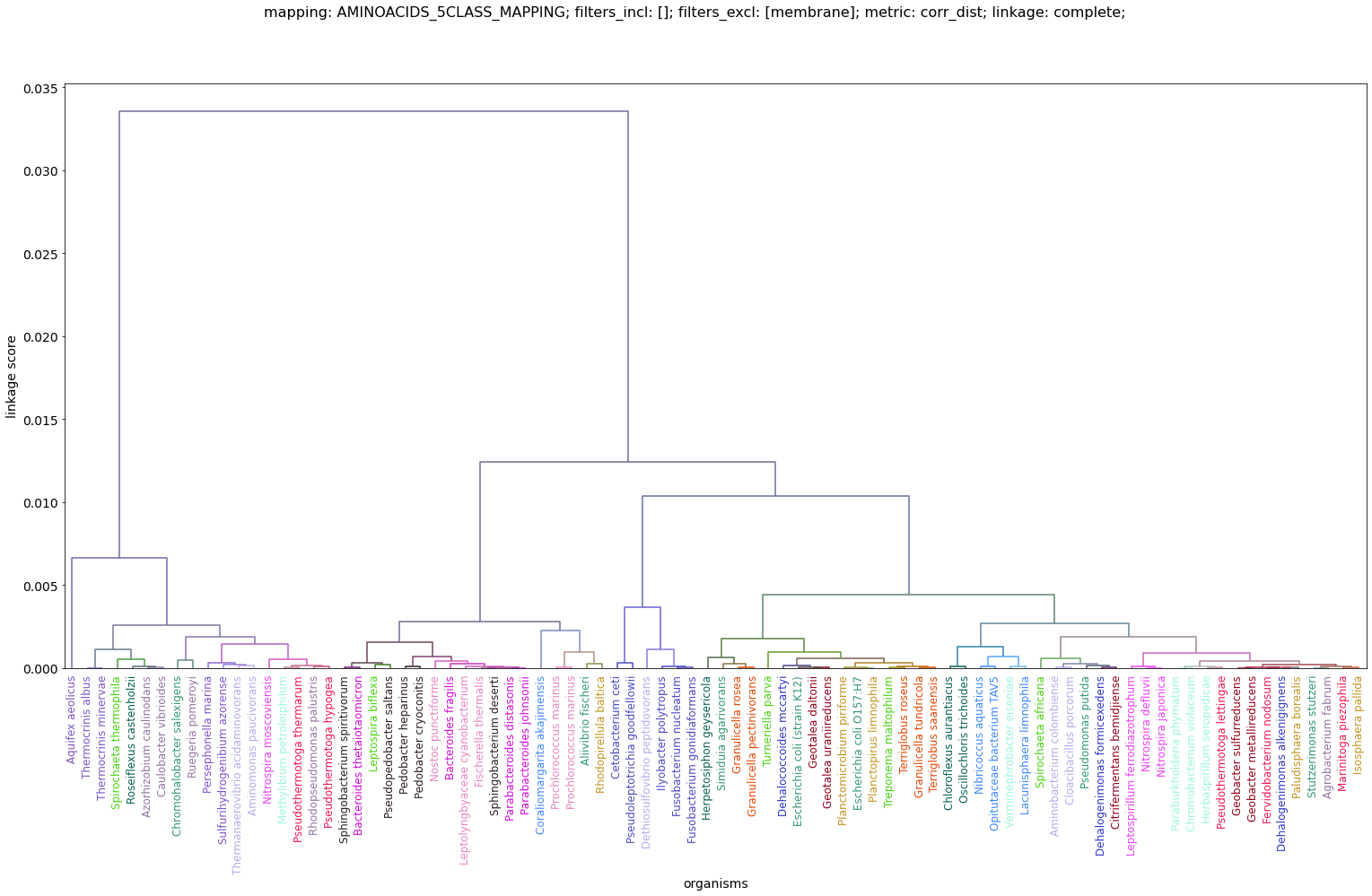

Supplement: Supplementary file 1 [file ijms-27-00109-s001.zip › kmers_supplementary/dendrograms/k1/AMINOACIDS_5CLASS_MAPPING/_membrane/corr_dist_complete.png]

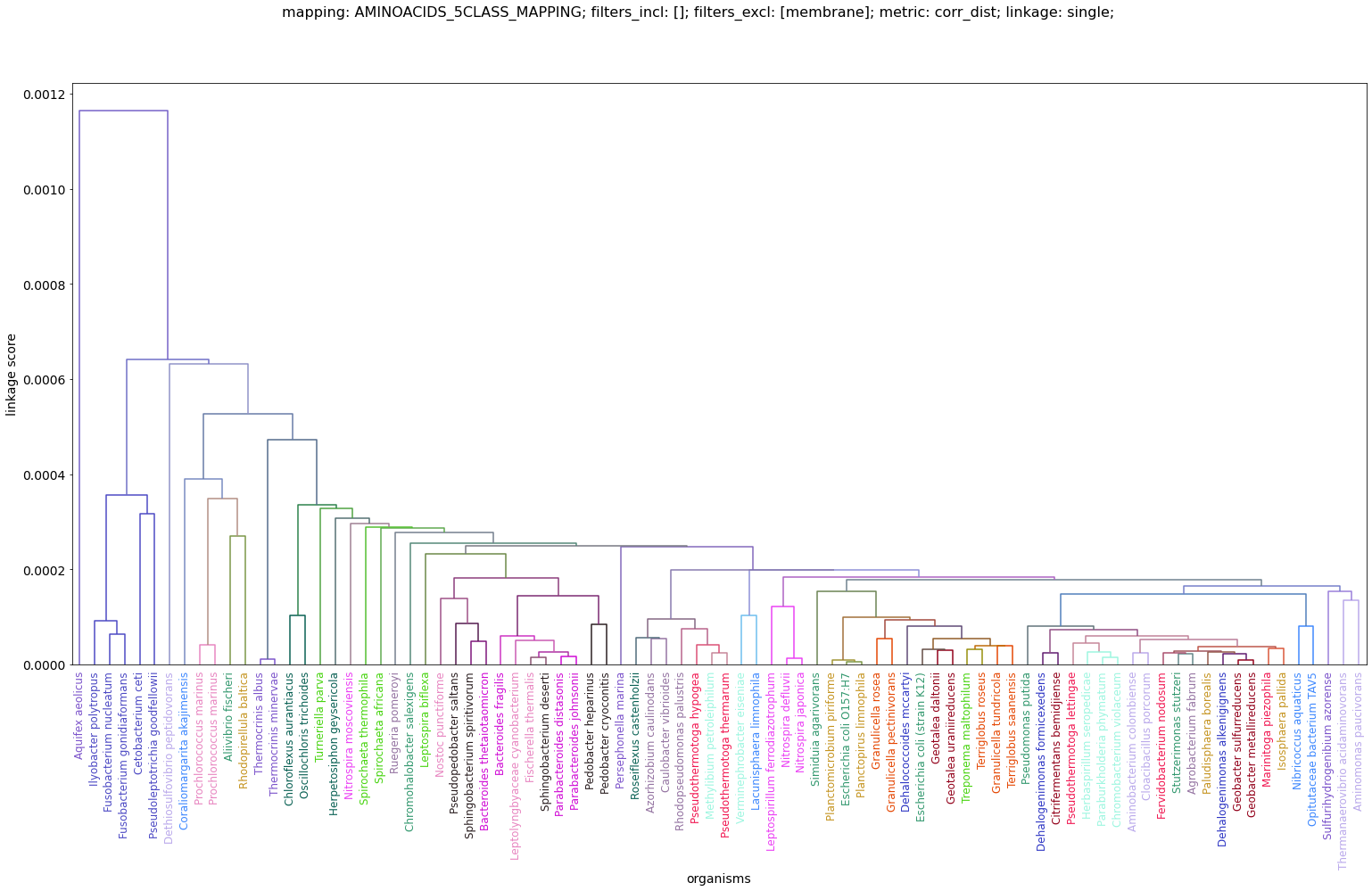

Supplement: Supplementary file 1 [file ijms-27-00109-s001.zip › kmers_supplementary/dendrograms/k1/AMINOACIDS_5CLASS_MAPPING/_membrane/corr_dist_single.png]

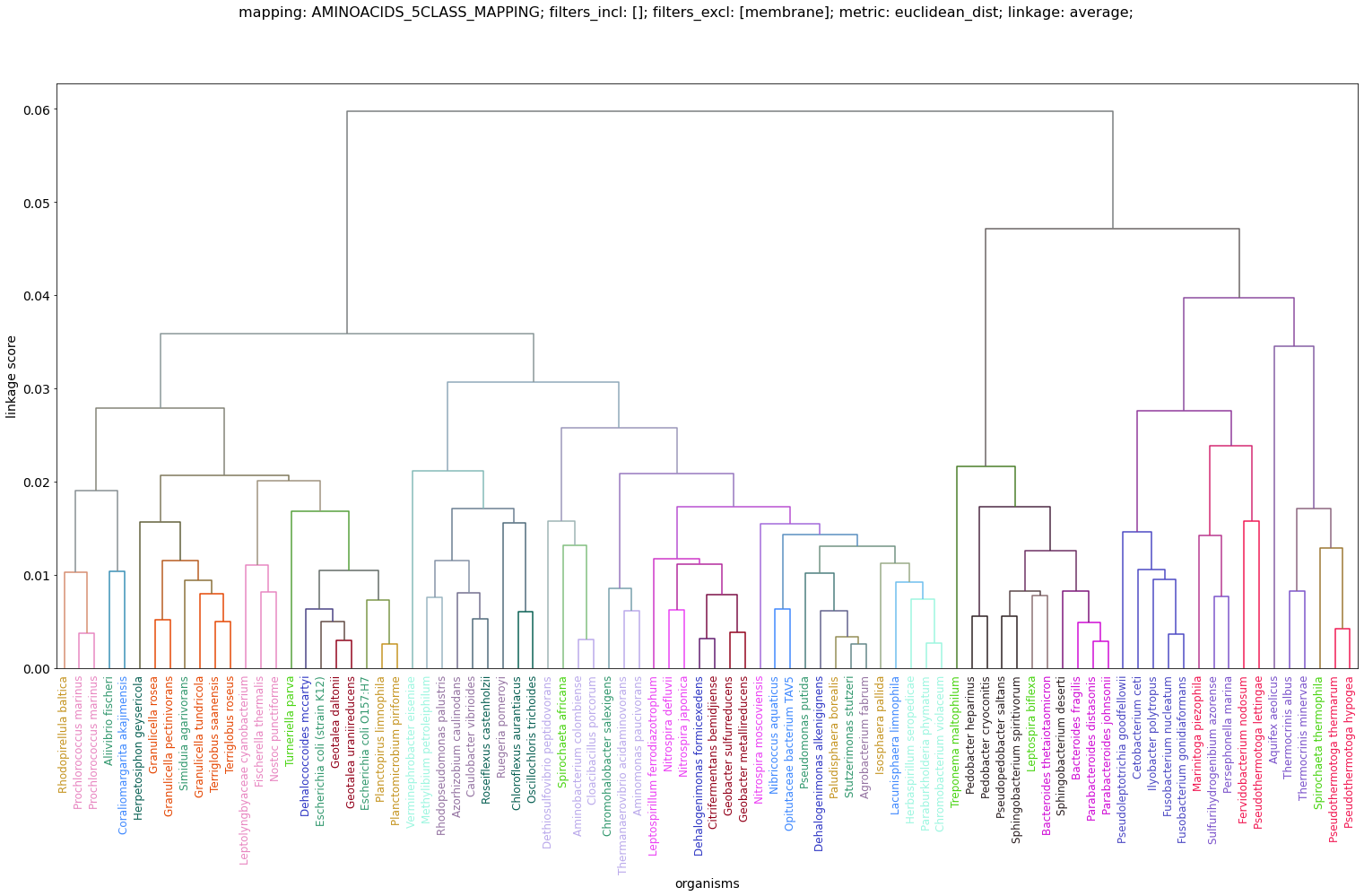

Supplement: Supplementary file 1 [file ijms-27-00109-s001.zip › kmers_supplementary/dendrograms/k1/AMINOACIDS_5CLASS_MAPPING/_membrane/euclidean_dist_average.png]

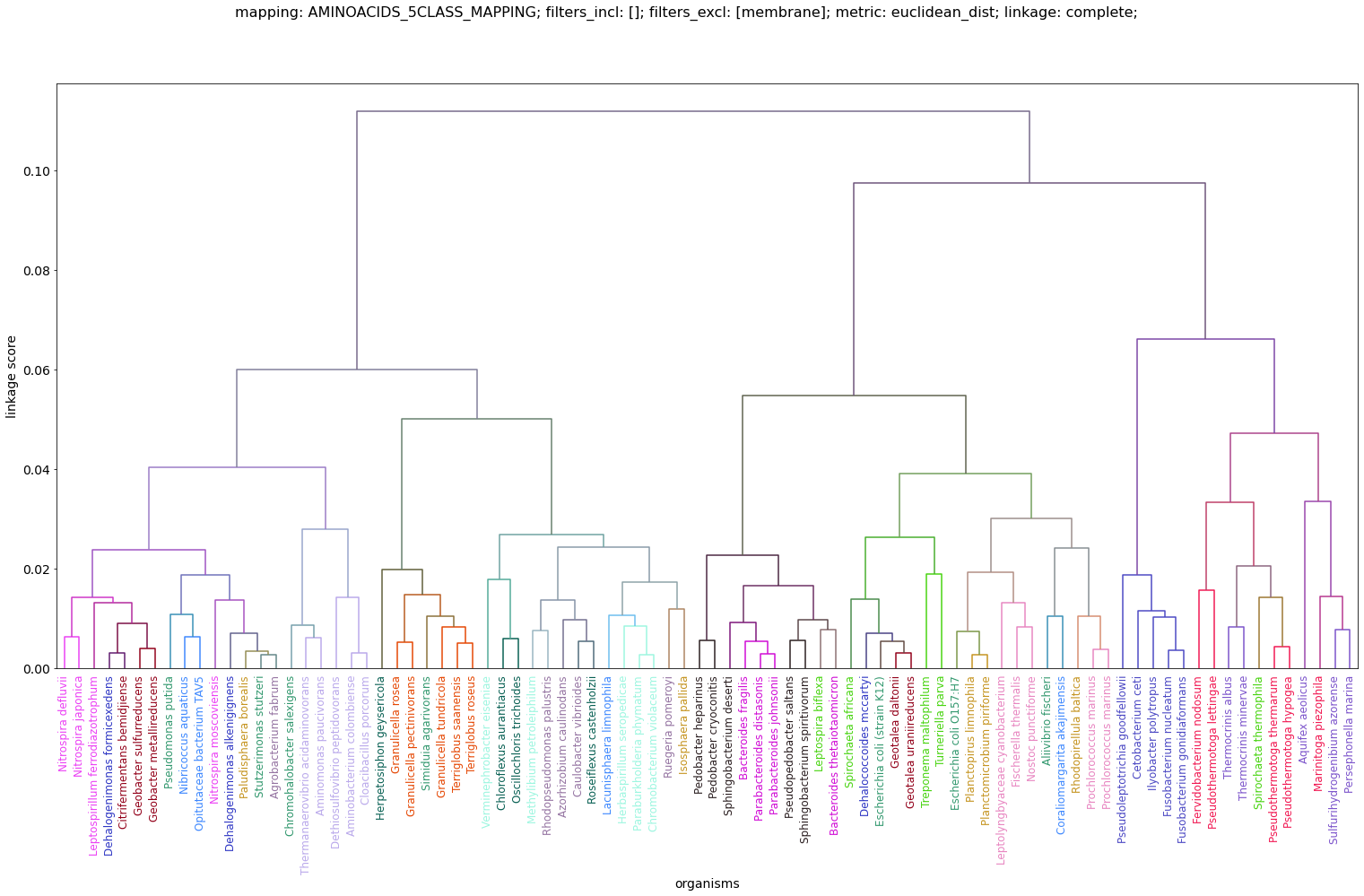

Supplement: Supplementary file 1 [file ijms-27-00109-s001.zip › kmers_supplementary/dendrograms/k1/AMINOACIDS_5CLASS_MAPPING/_membrane/euclidean_dist_complete.png]

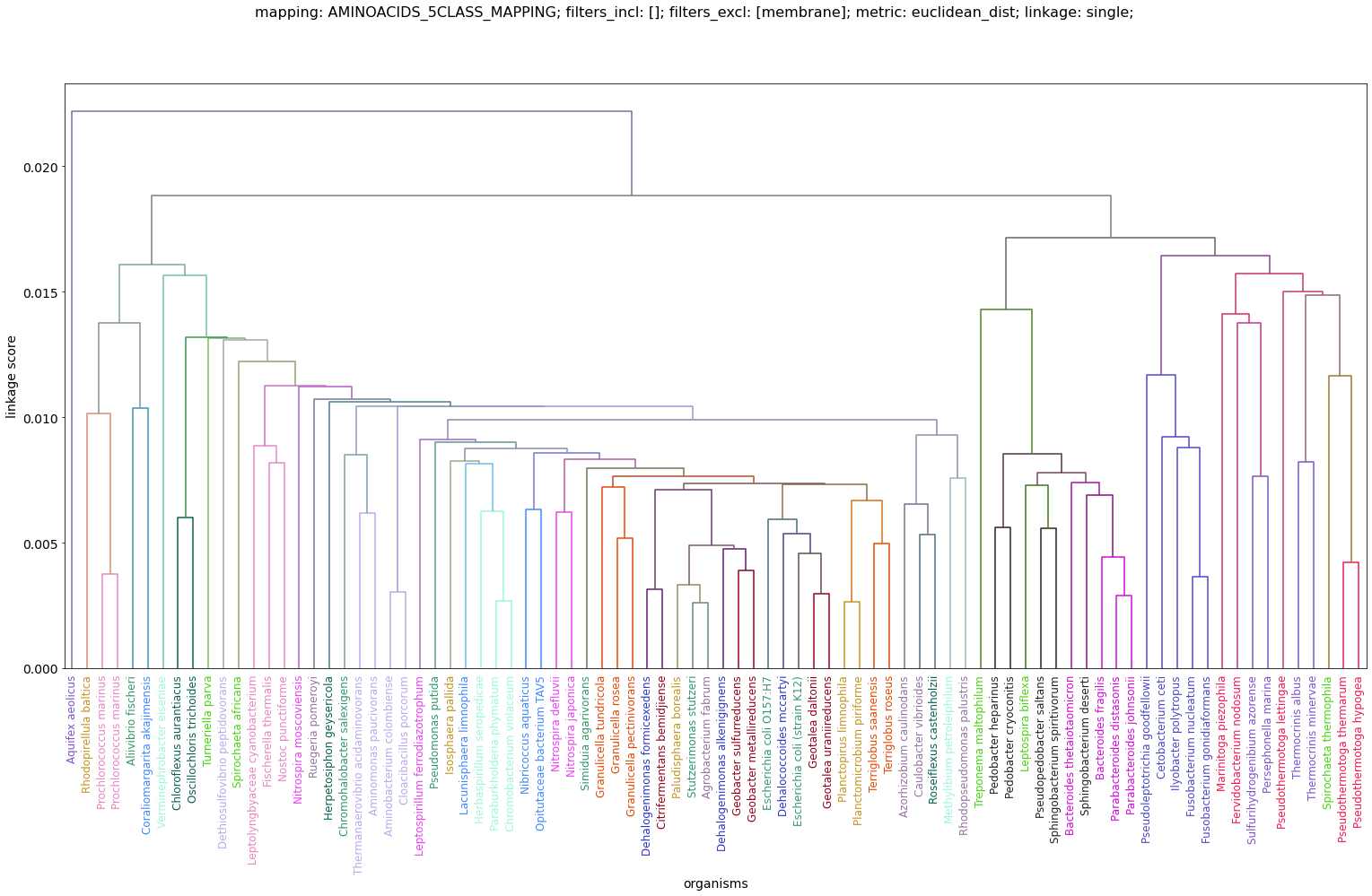

Supplement: Supplementary file 1 [file ijms-27-00109-s001.zip › kmers_supplementary/dendrograms/k1/AMINOACIDS_5CLASS_MAPPING/_membrane/euclidean_dist_single.png]

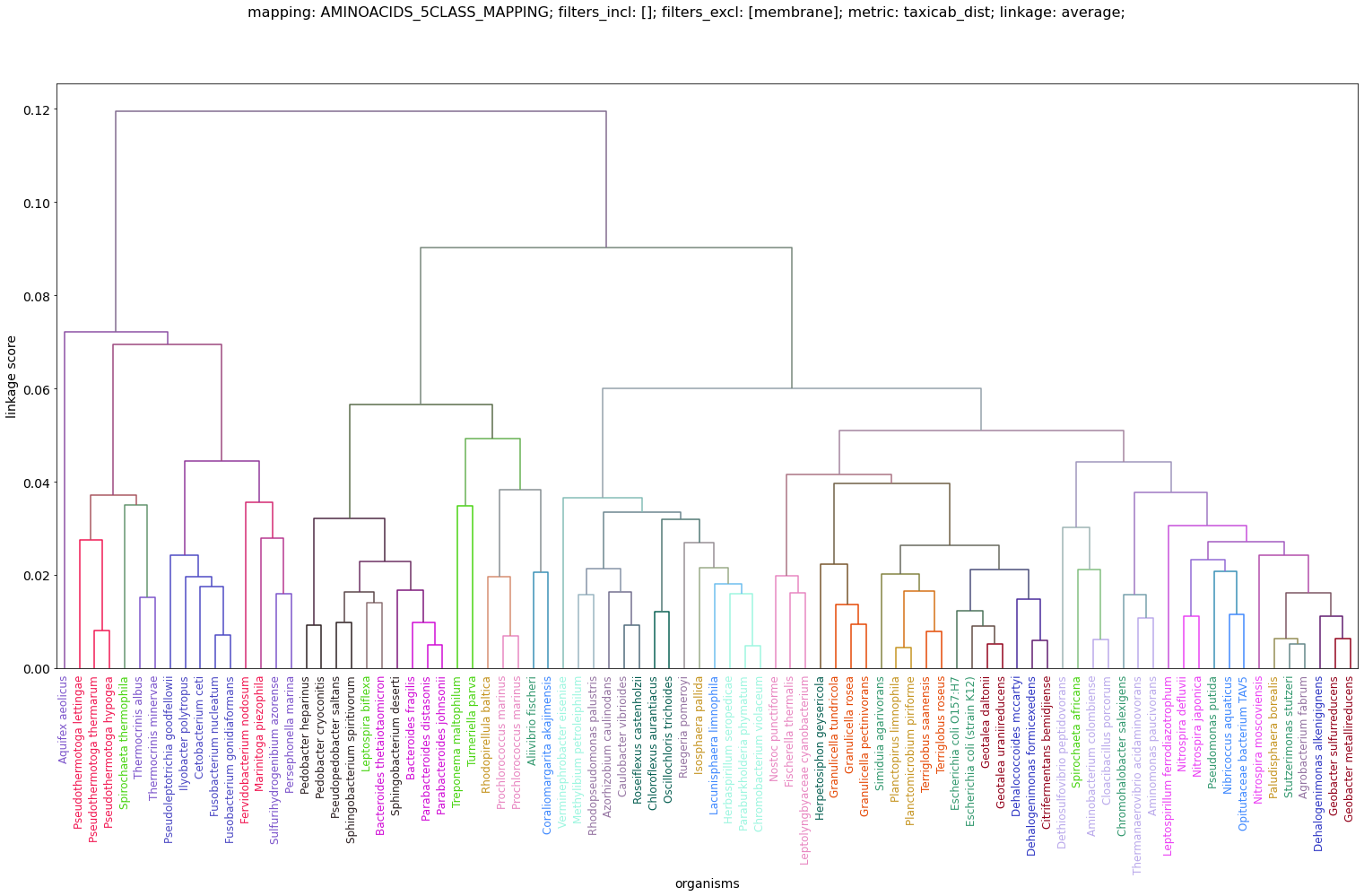

Supplement: Supplementary file 1 [file ijms-27-00109-s001.zip › kmers_supplementary/dendrograms/k1/AMINOACIDS_5CLASS_MAPPING/_membrane/taxicab_dist_average.png]

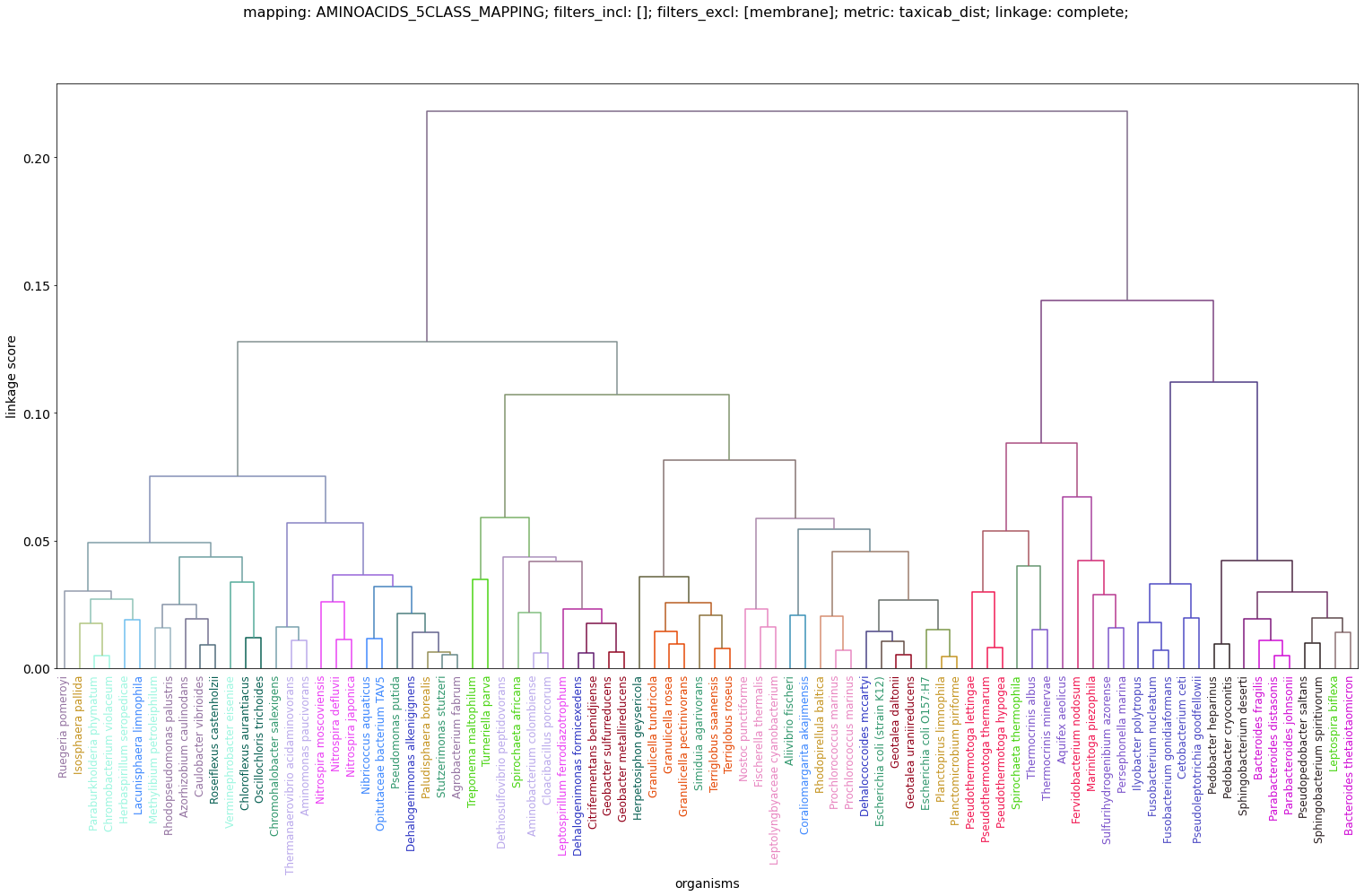

Supplement: Supplementary file 1 [file ijms-27-00109-s001.zip › kmers_supplementary/dendrograms/k1/AMINOACIDS_5CLASS_MAPPING/_membrane/taxicab_dist_complete.png]

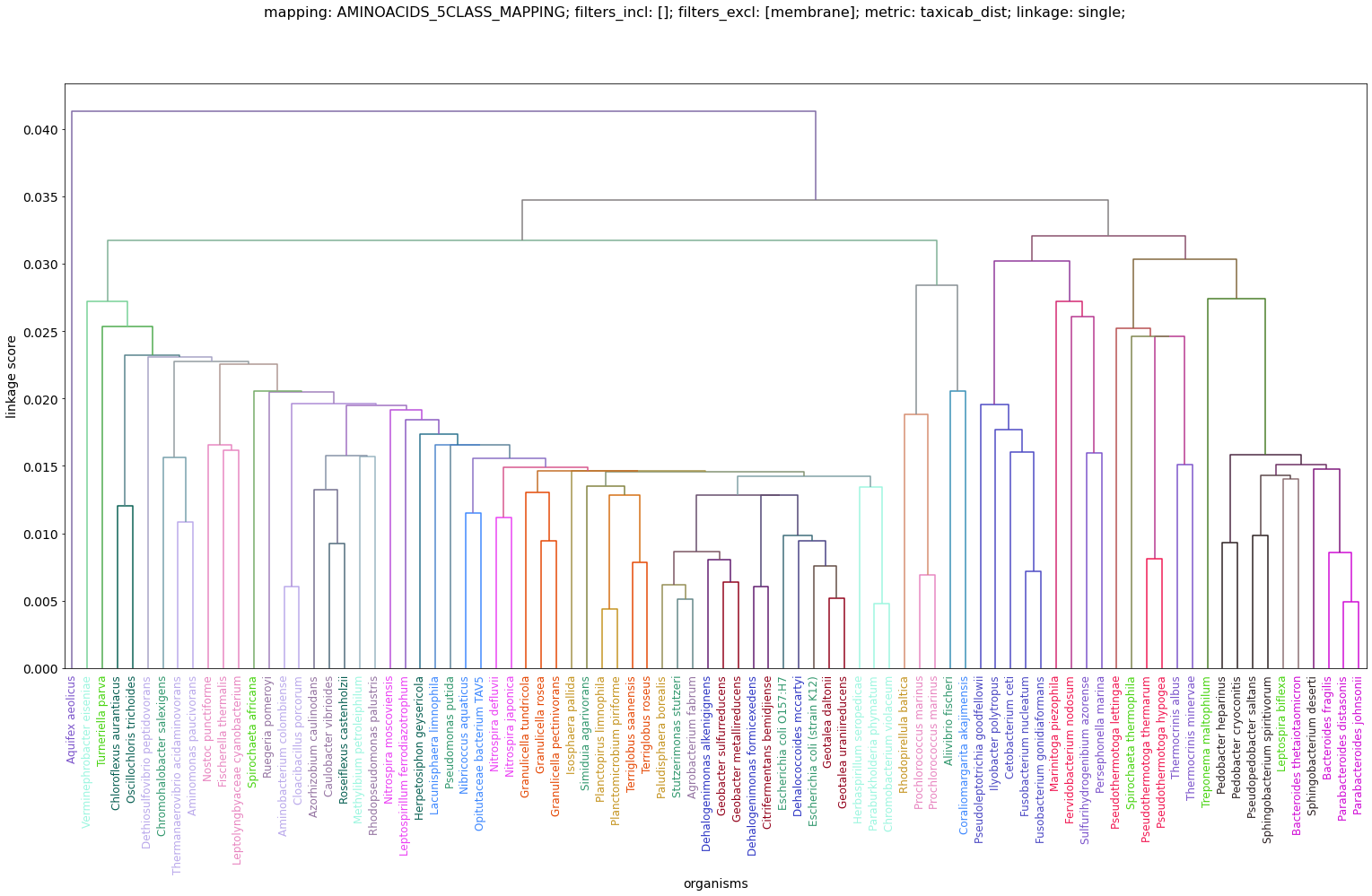

Supplement: Supplementary file 1 [file ijms-27-00109-s001.zip › kmers_supplementary/dendrograms/k1/AMINOACIDS_5CLASS_MAPPING/_membrane/taxicab_dist_single.png]

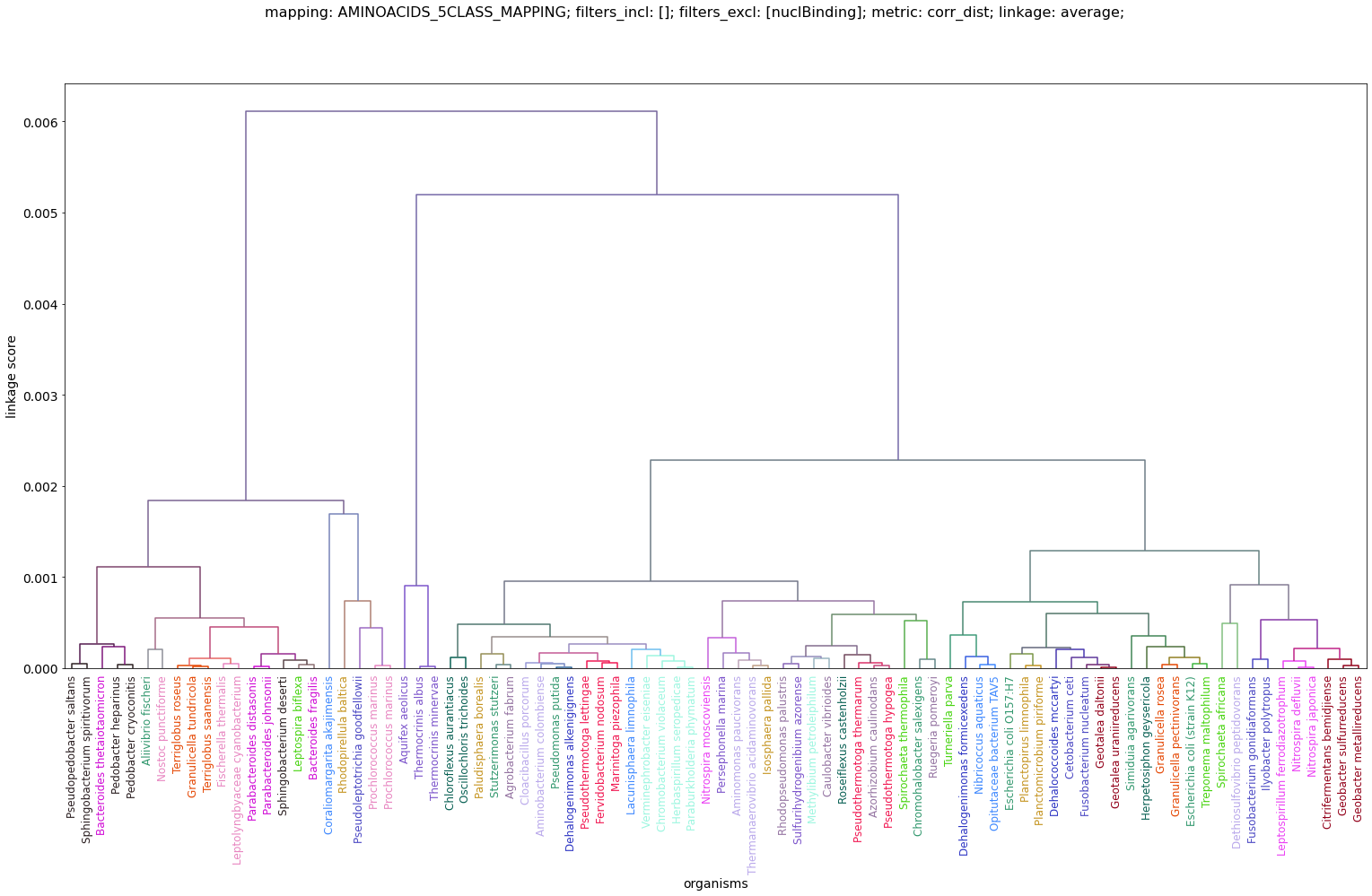

Supplement: Supplementary file 1 [file ijms-27-00109-s001.zip › kmers_supplementary/dendrograms/k1/AMINOACIDS_5CLASS_MAPPING/_nuclBinding/corr_dist_average.png]

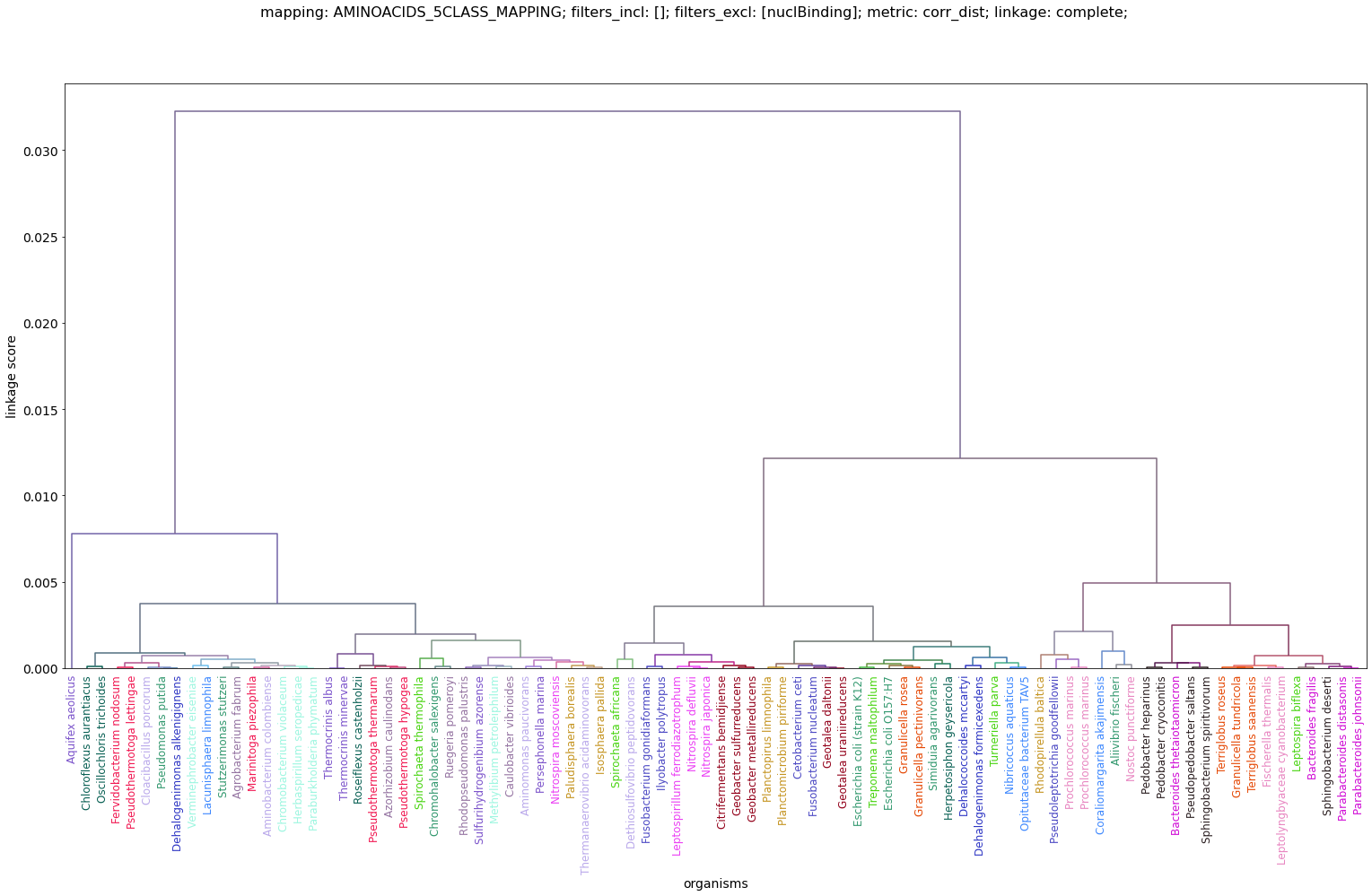

Supplement: Supplementary file 1 [file ijms-27-00109-s001.zip › kmers_supplementary/dendrograms/k1/AMINOACIDS_5CLASS_MAPPING/_nuclBinding/corr_dist_complete.png]

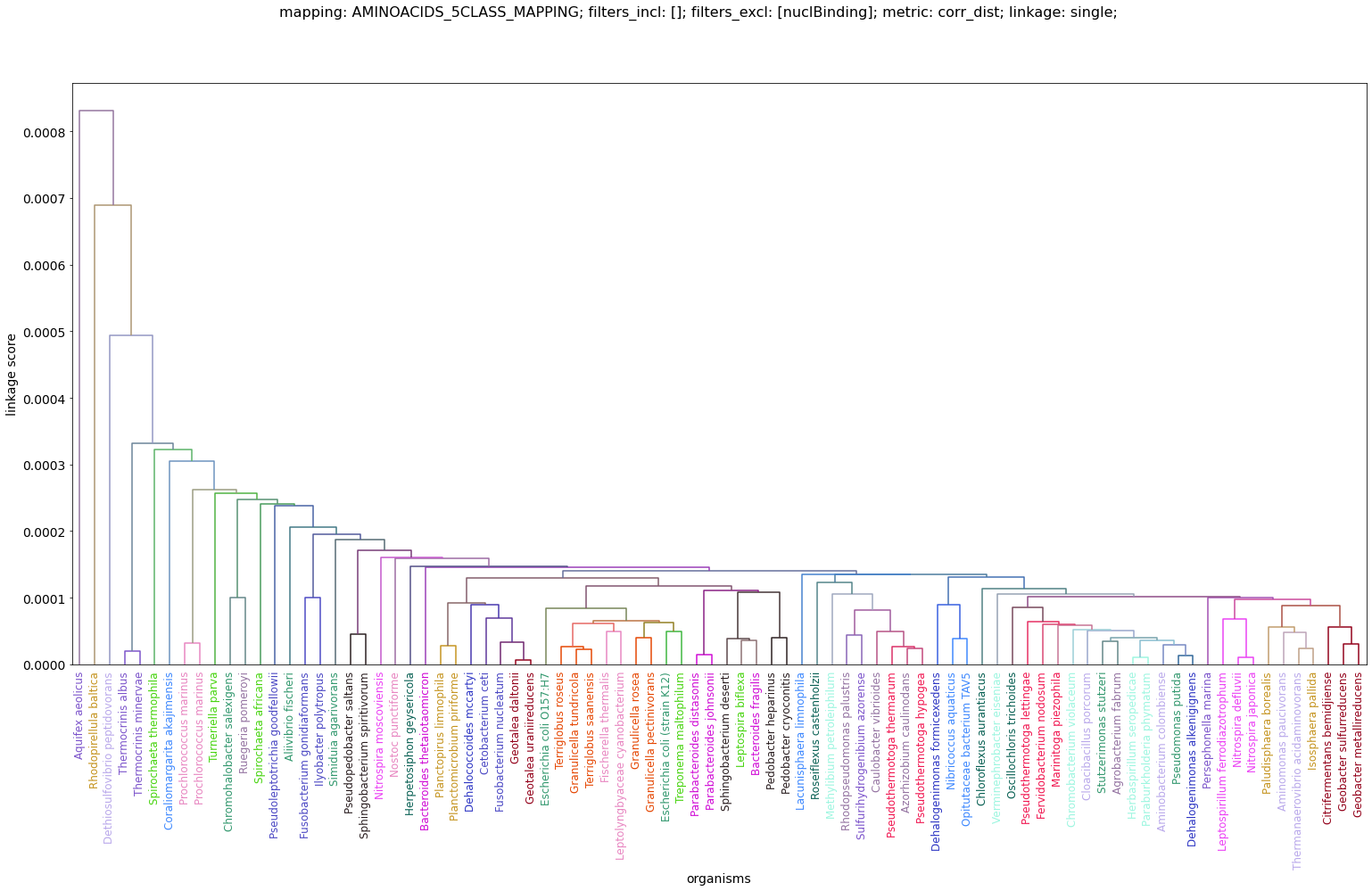

Supplement: Supplementary file 1 [file ijms-27-00109-s001.zip › kmers_supplementary/dendrograms/k1/AMINOACIDS_5CLASS_MAPPING/_nuclBinding/corr_dist_single.png]

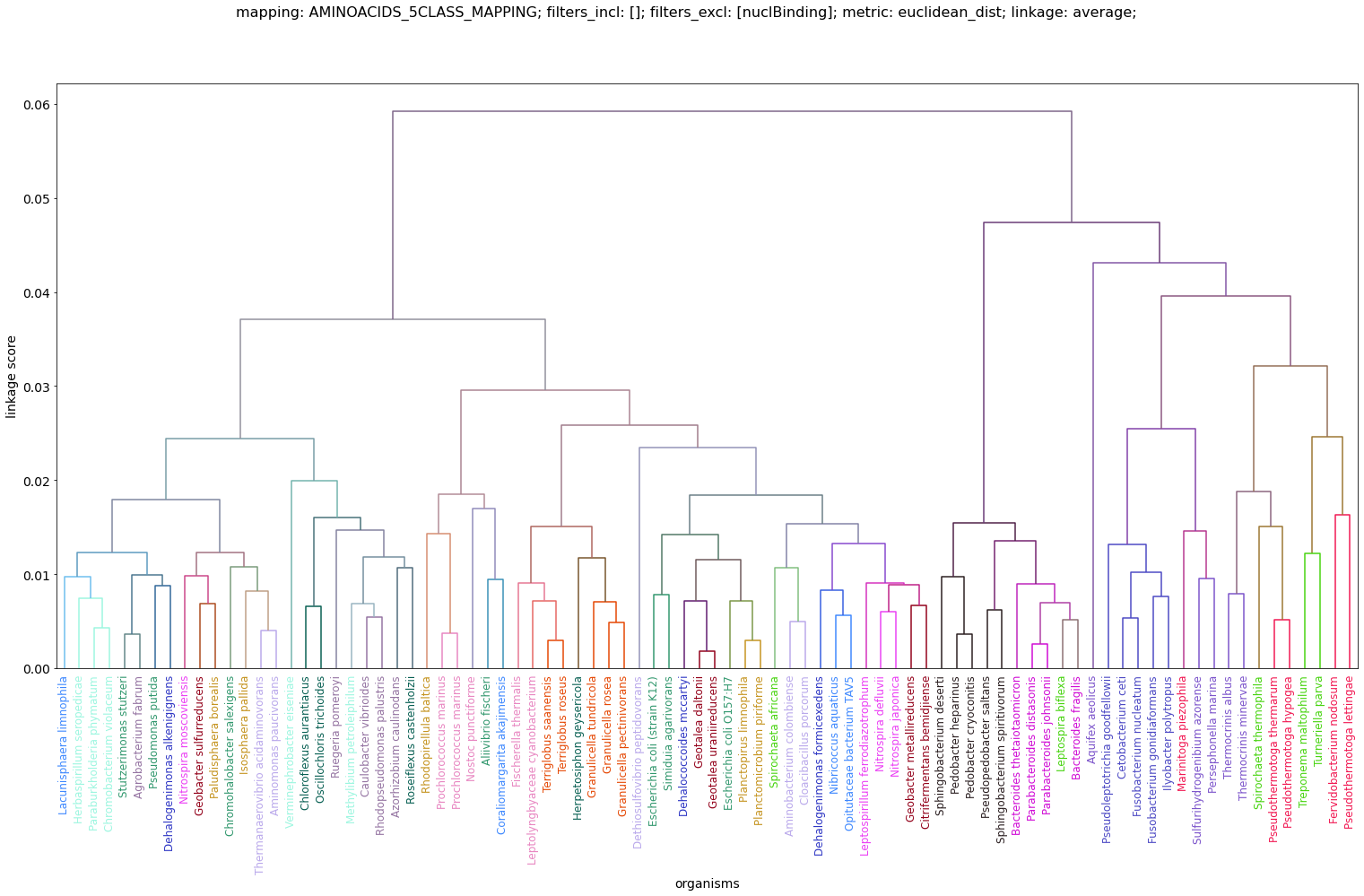

Supplement: Supplementary file 1 [file ijms-27-00109-s001.zip › kmers_supplementary/dendrograms/k1/AMINOACIDS_5CLASS_MAPPING/_nuclBinding/euclidean_dist_average.png]

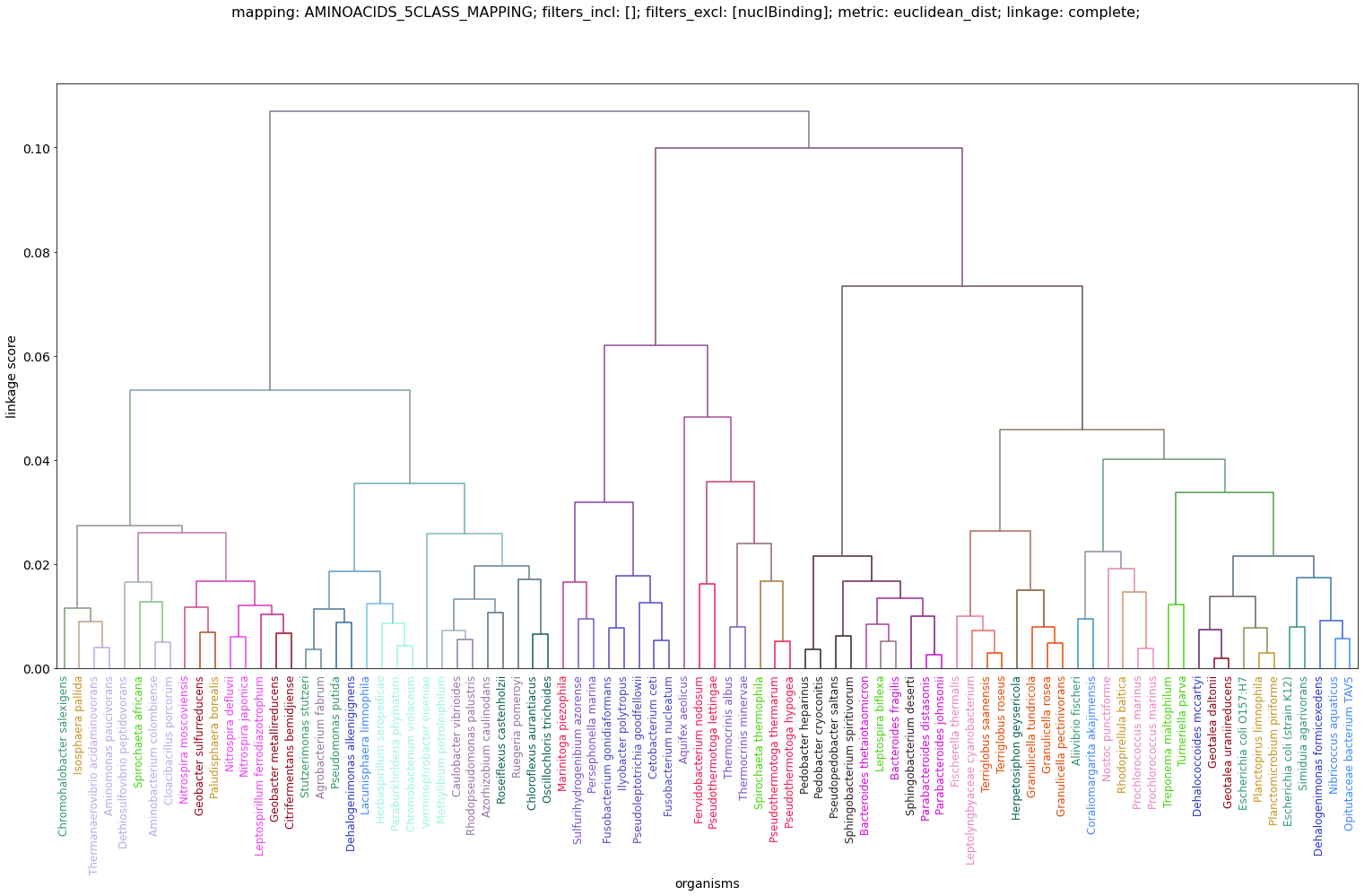

Supplement: Supplementary file 1 [file ijms-27-00109-s001.zip › kmers_supplementary/dendrograms/k1/AMINOACIDS_5CLASS_MAPPING/_nuclBinding/euclidean_dist_complete.png]

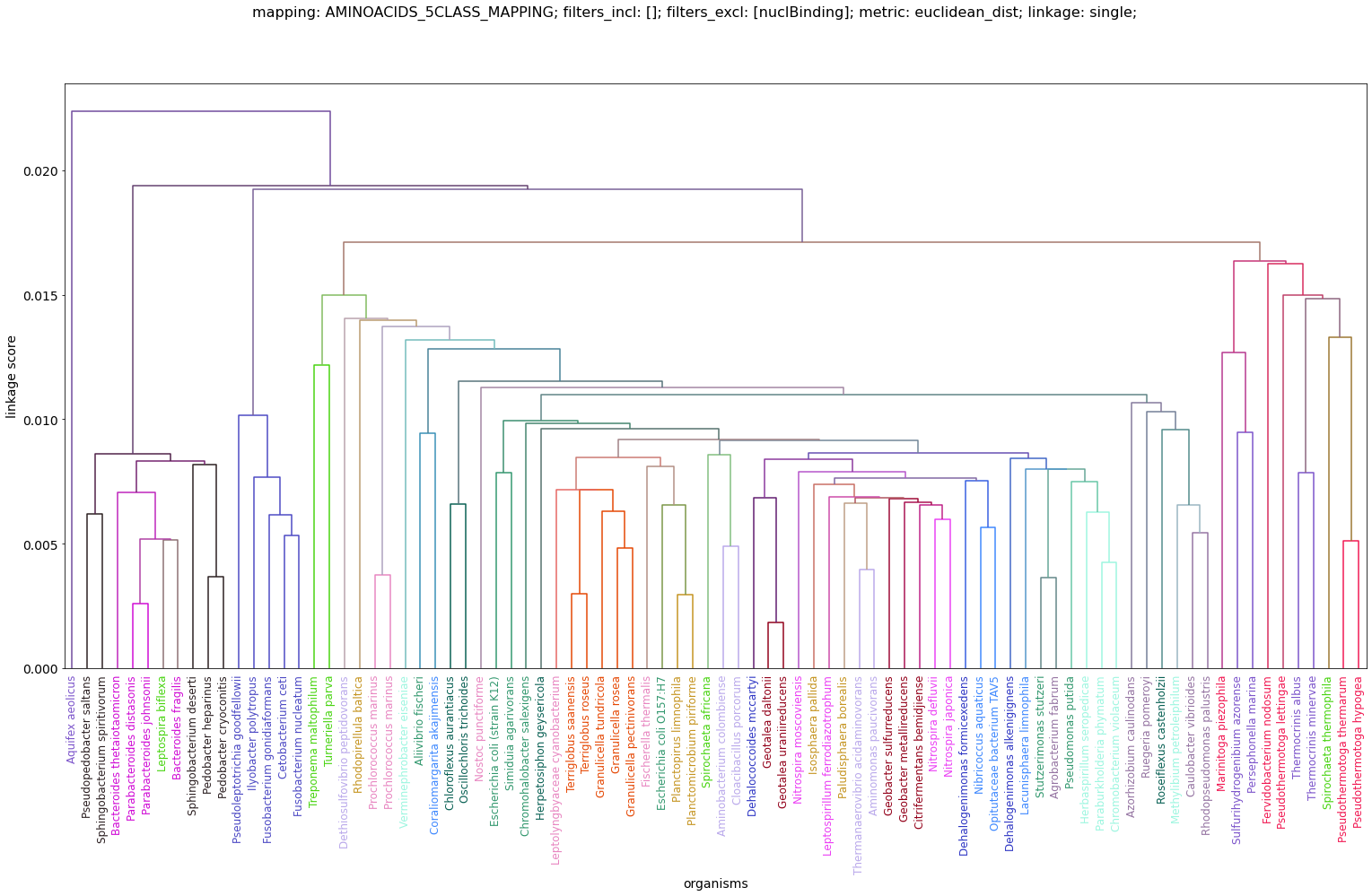

Supplement: Supplementary file 1 [file ijms-27-00109-s001.zip › kmers_supplementary/dendrograms/k1/AMINOACIDS_5CLASS_MAPPING/_nuclBinding/euclidean_dist_single.png]

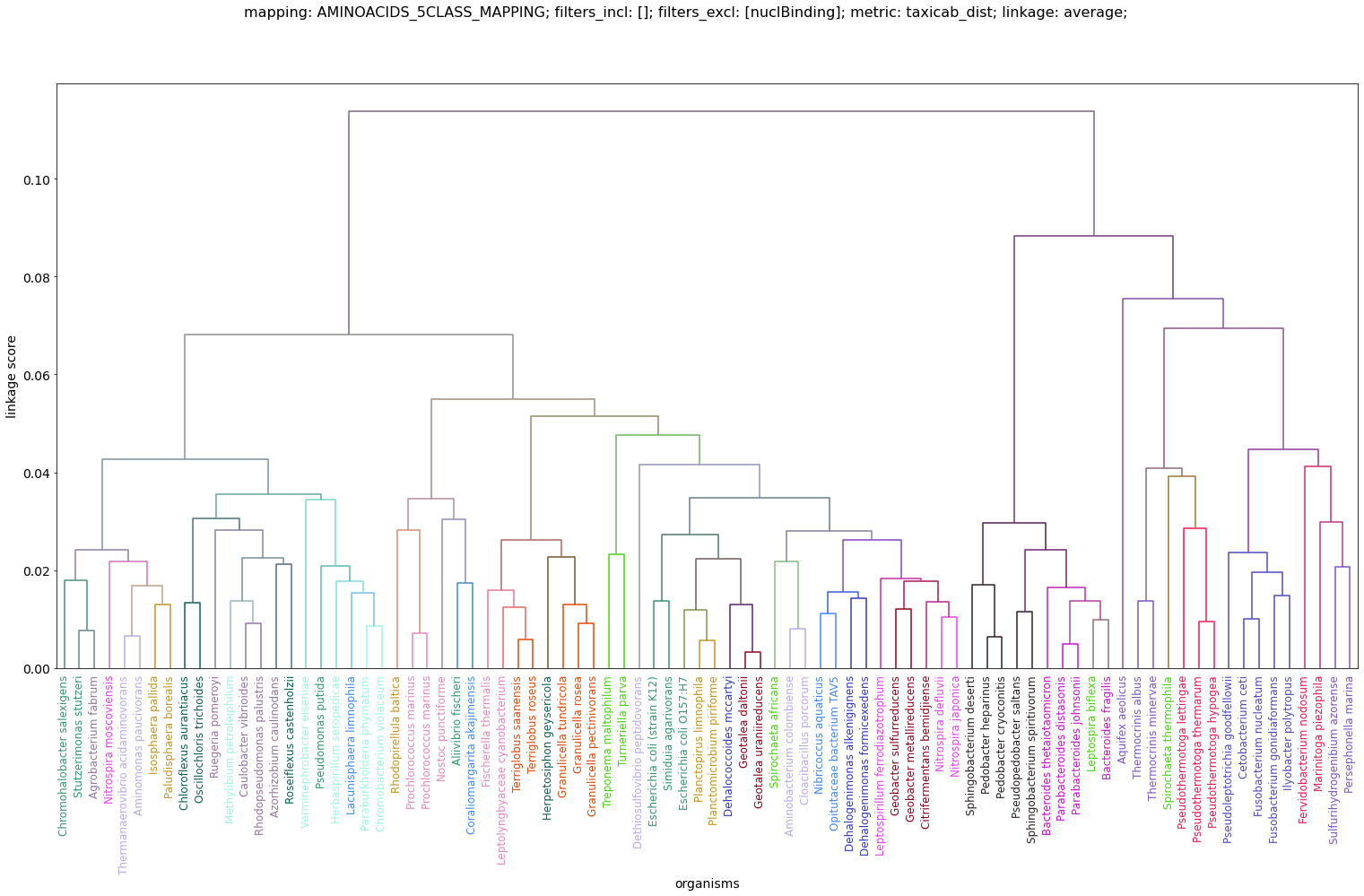

Supplement: Supplementary file 1 [file ijms-27-00109-s001.zip › kmers_supplementary/dendrograms/k1/AMINOACIDS_5CLASS_MAPPING/_nuclBinding/taxicab_dist_average.png]

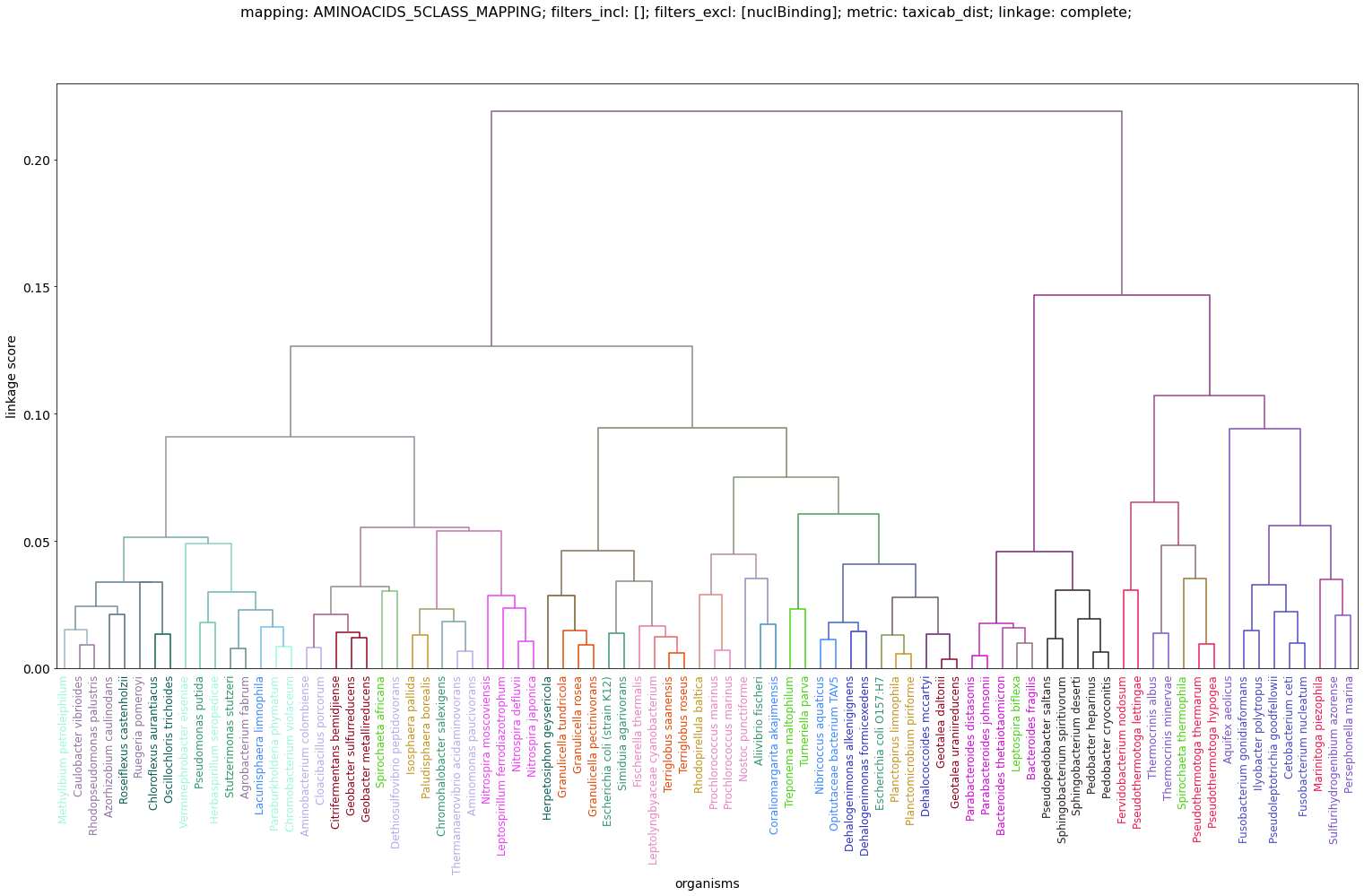

Supplement: Supplementary file 1 [file ijms-27-00109-s001.zip › kmers_supplementary/dendrograms/k1/AMINOACIDS_5CLASS_MAPPING/_nuclBinding/taxicab_dist_complete.png]

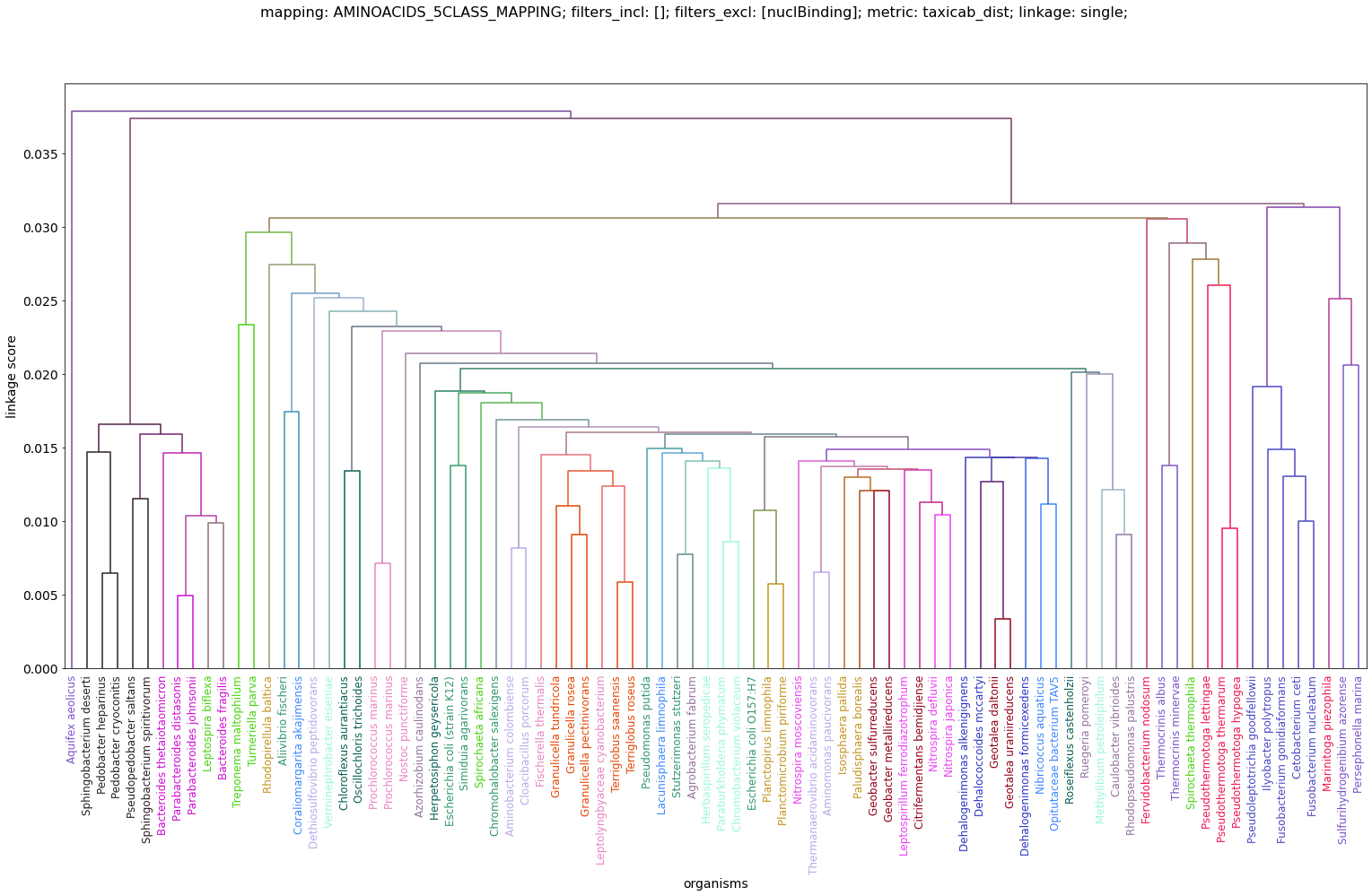

Supplement: Supplementary file 1 [file ijms-27-00109-s001.zip › kmers_supplementary/dendrograms/k1/AMINOACIDS_5CLASS_MAPPING/_nuclBinding/taxicab_dist_single.png]

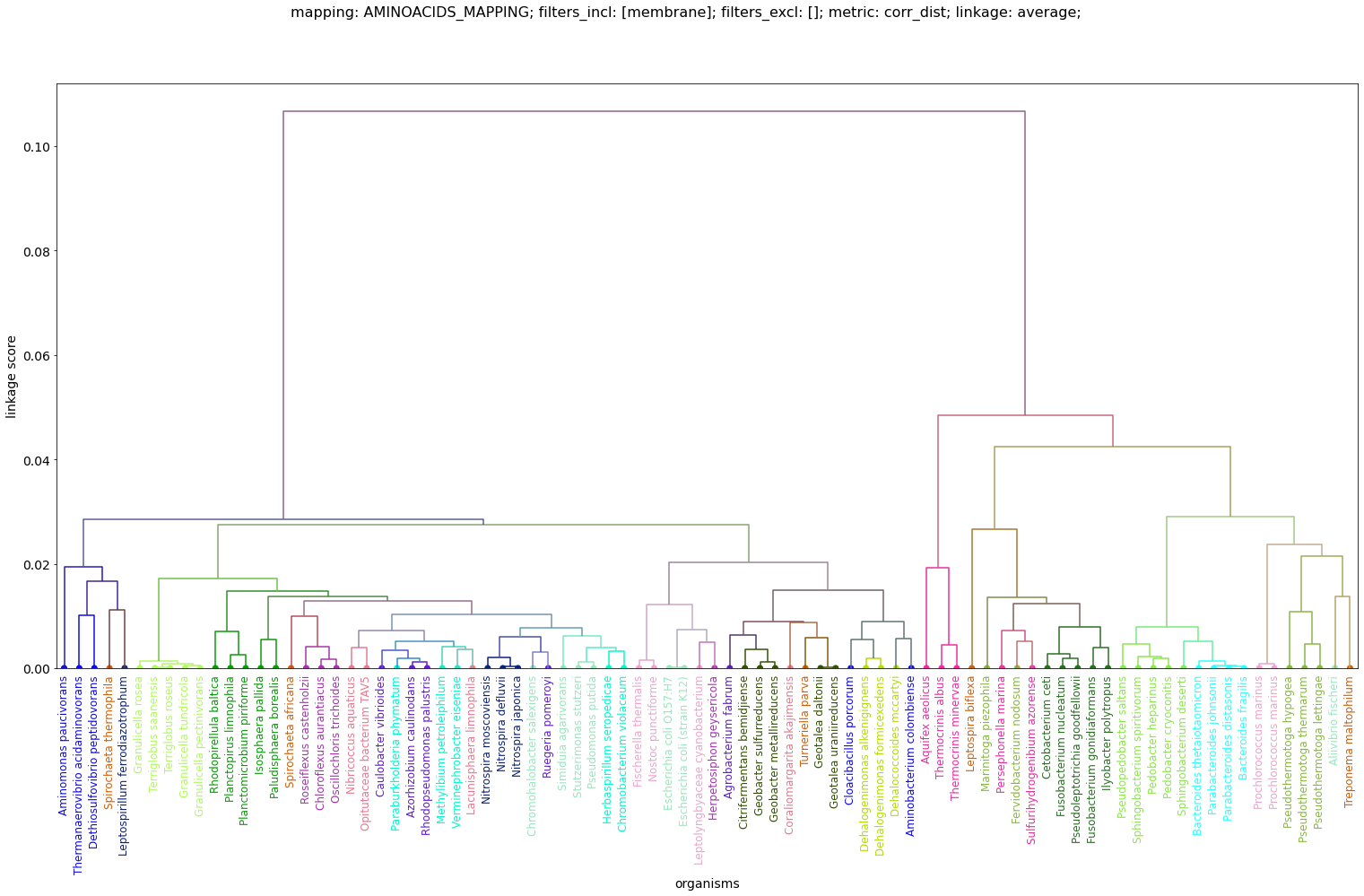

Supplement: Supplementary file 1 [file ijms-27-00109-s001.zip › kmers_supplementary/dendrograms/k1/AMINOACIDS_MAPPING/membrane_/corr_dist_average.png]

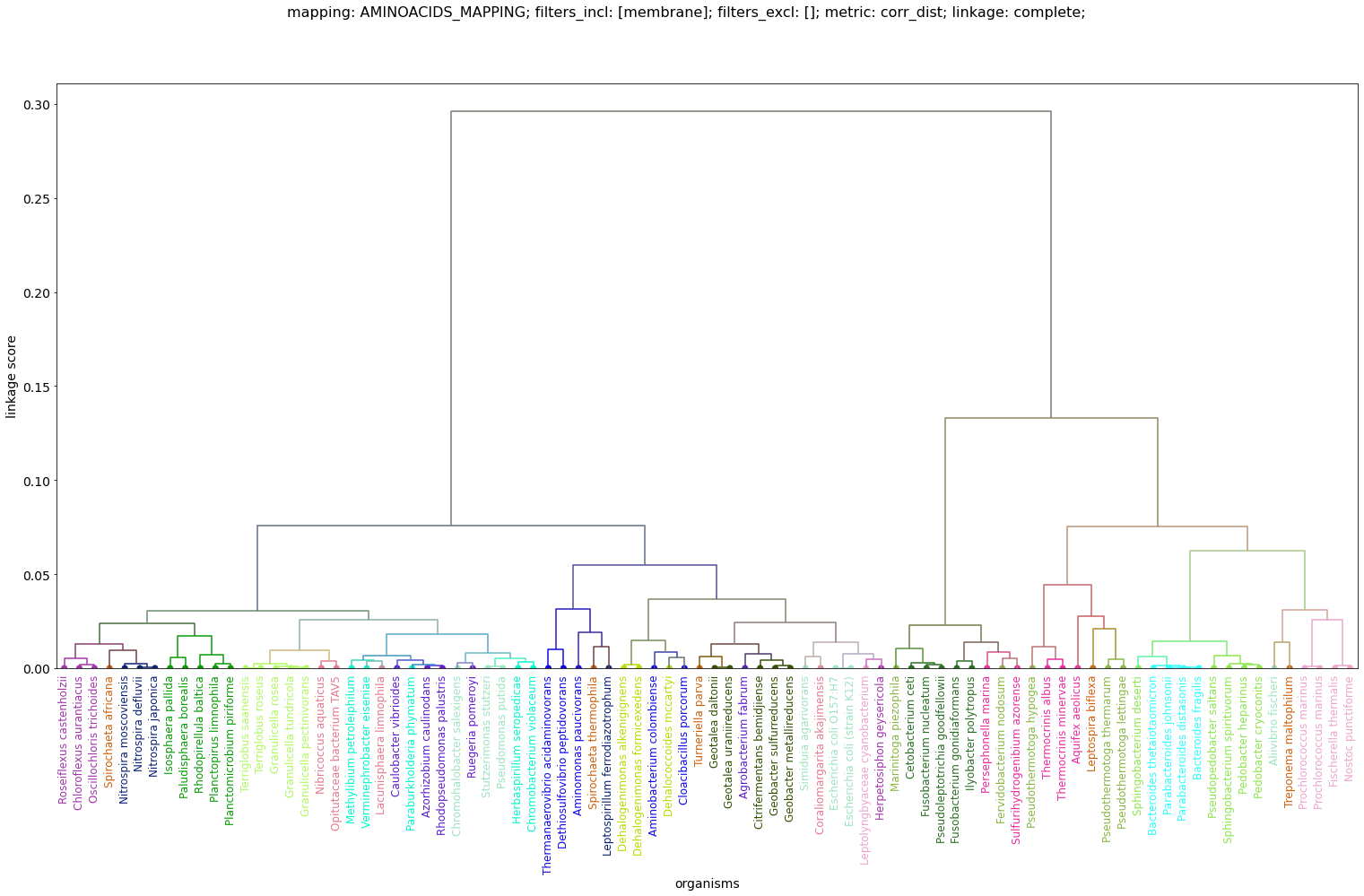

Supplement: Supplementary file 1 [file ijms-27-00109-s001.zip › kmers_supplementary/dendrograms/k1/AMINOACIDS_MAPPING/membrane_/corr_dist_complete.png]

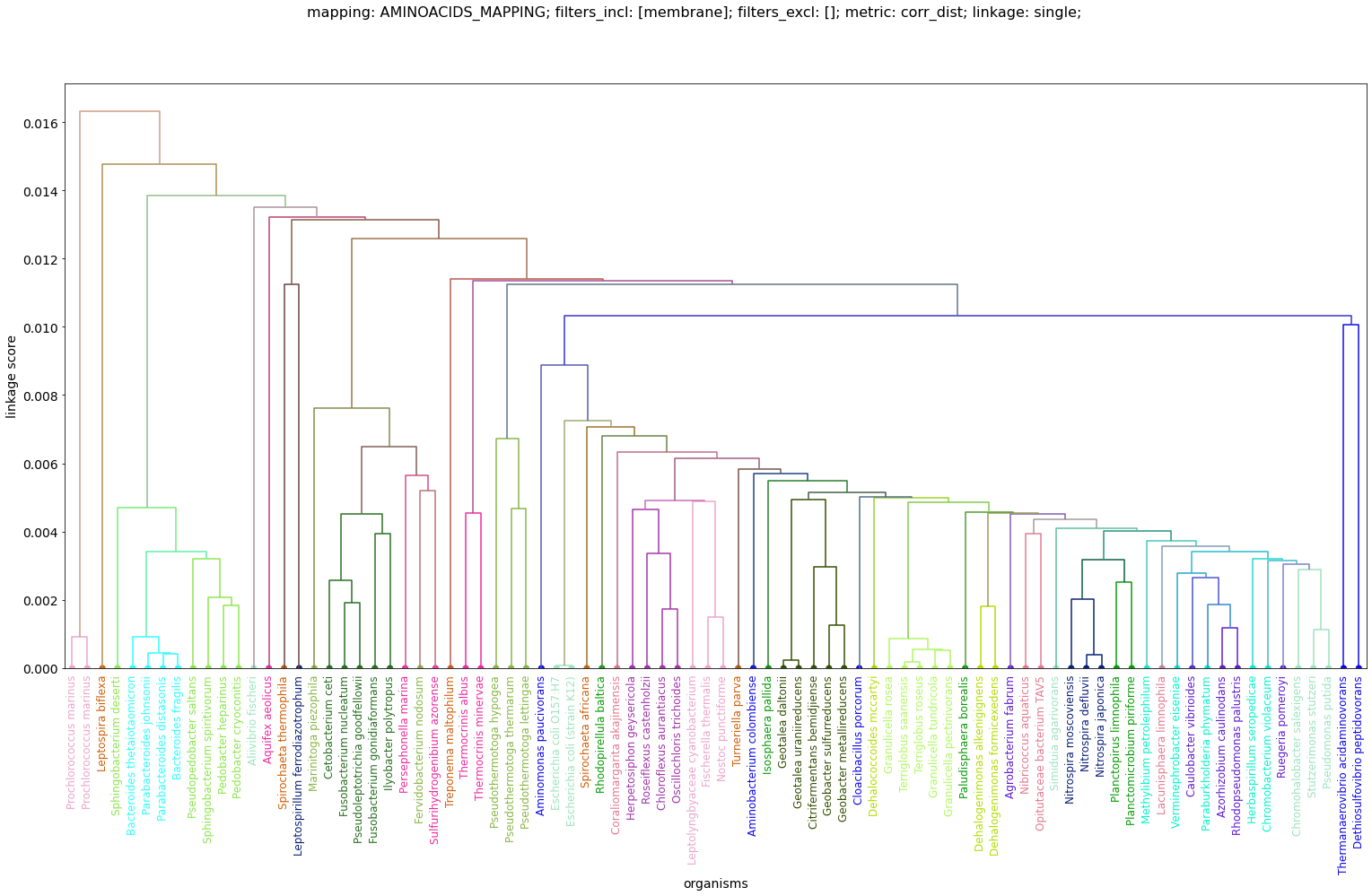

Supplement: Supplementary file 1 [file ijms-27-00109-s001.zip › kmers_supplementary/dendrograms/k1/AMINOACIDS_MAPPING/membrane_/corr_dist_single.png]

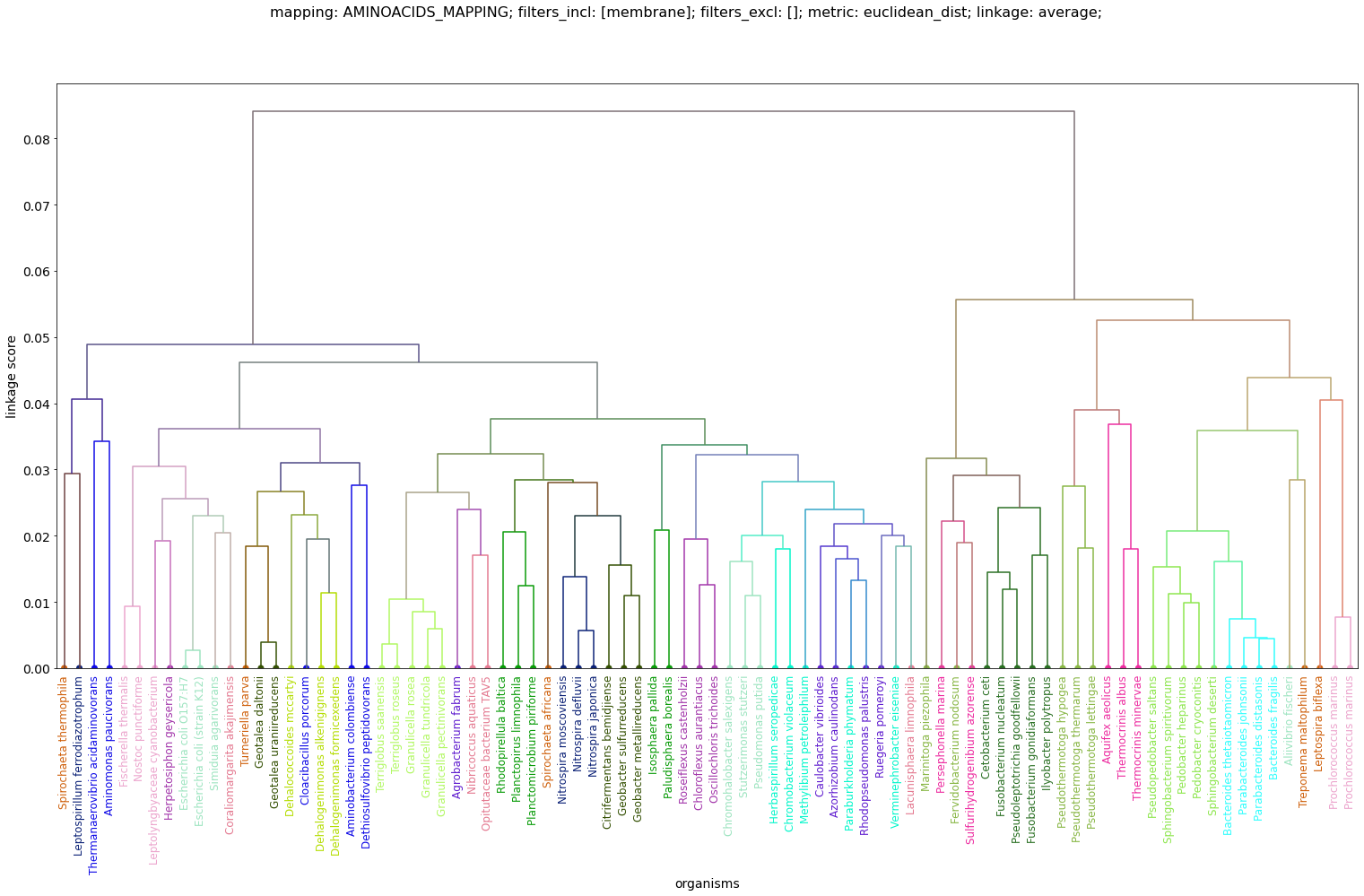

Supplement: Supplementary file 1 [file ijms-27-00109-s001.zip › kmers_supplementary/dendrograms/k1/AMINOACIDS_MAPPING/membrane_/euclidean_dist_average.png]

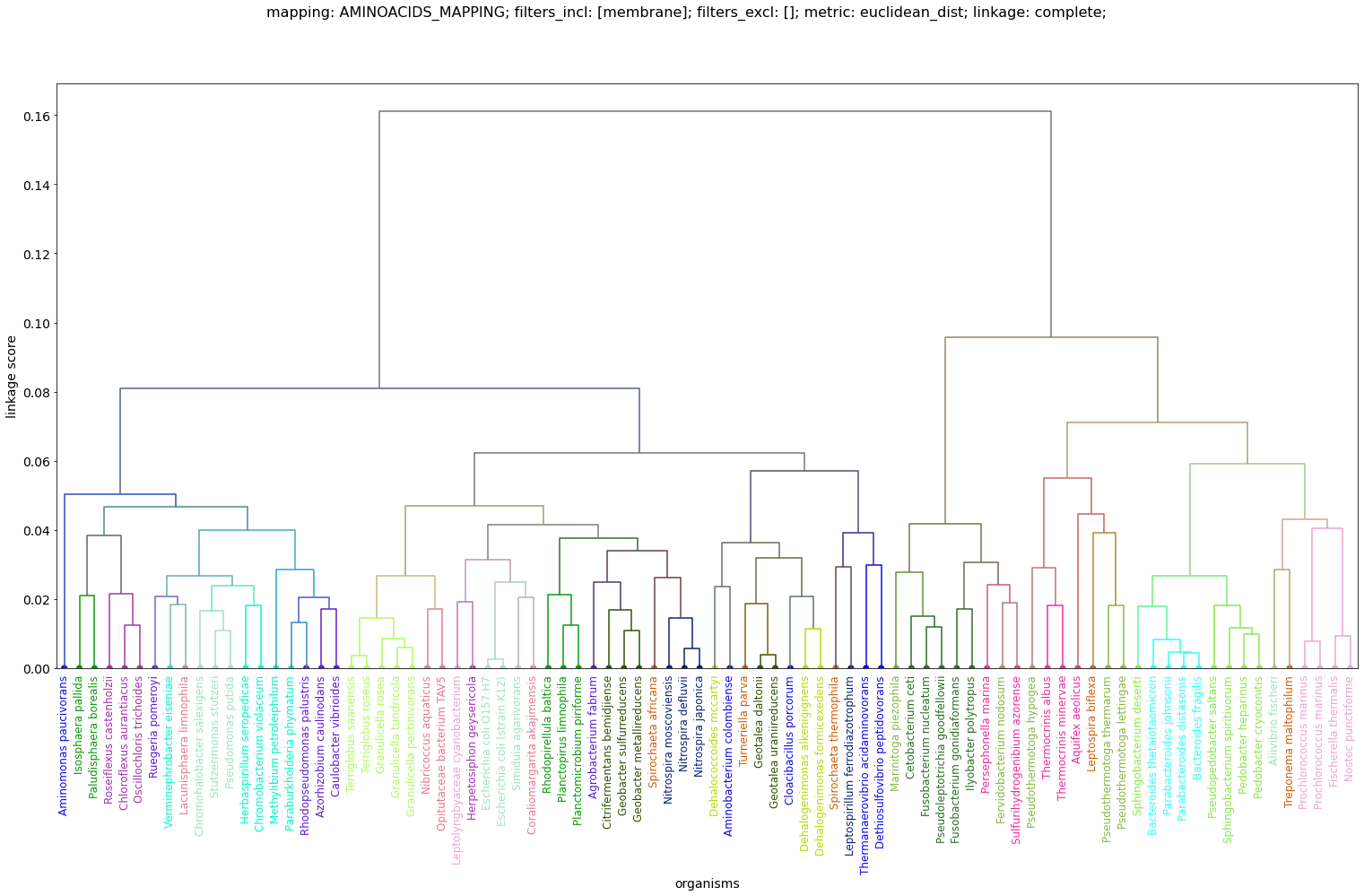

Supplement: Supplementary file 1 [file ijms-27-00109-s001.zip › kmers_supplementary/dendrograms/k1/AMINOACIDS_MAPPING/membrane_/euclidean_dist_complete.png]

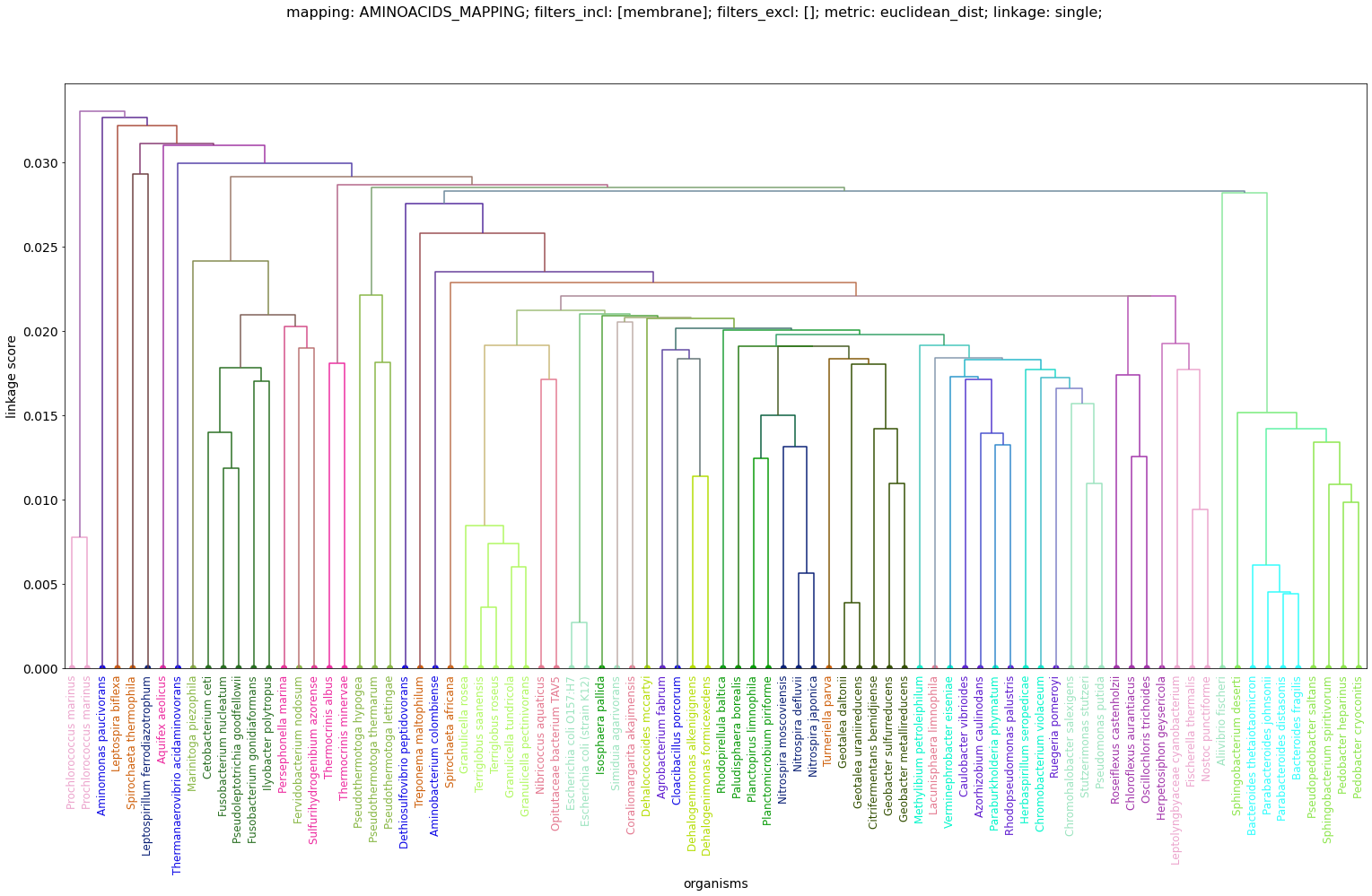

Supplement: Supplementary file 1 [file ijms-27-00109-s001.zip › kmers_supplementary/dendrograms/k1/AMINOACIDS_MAPPING/membrane_/euclidean_dist_single.png]

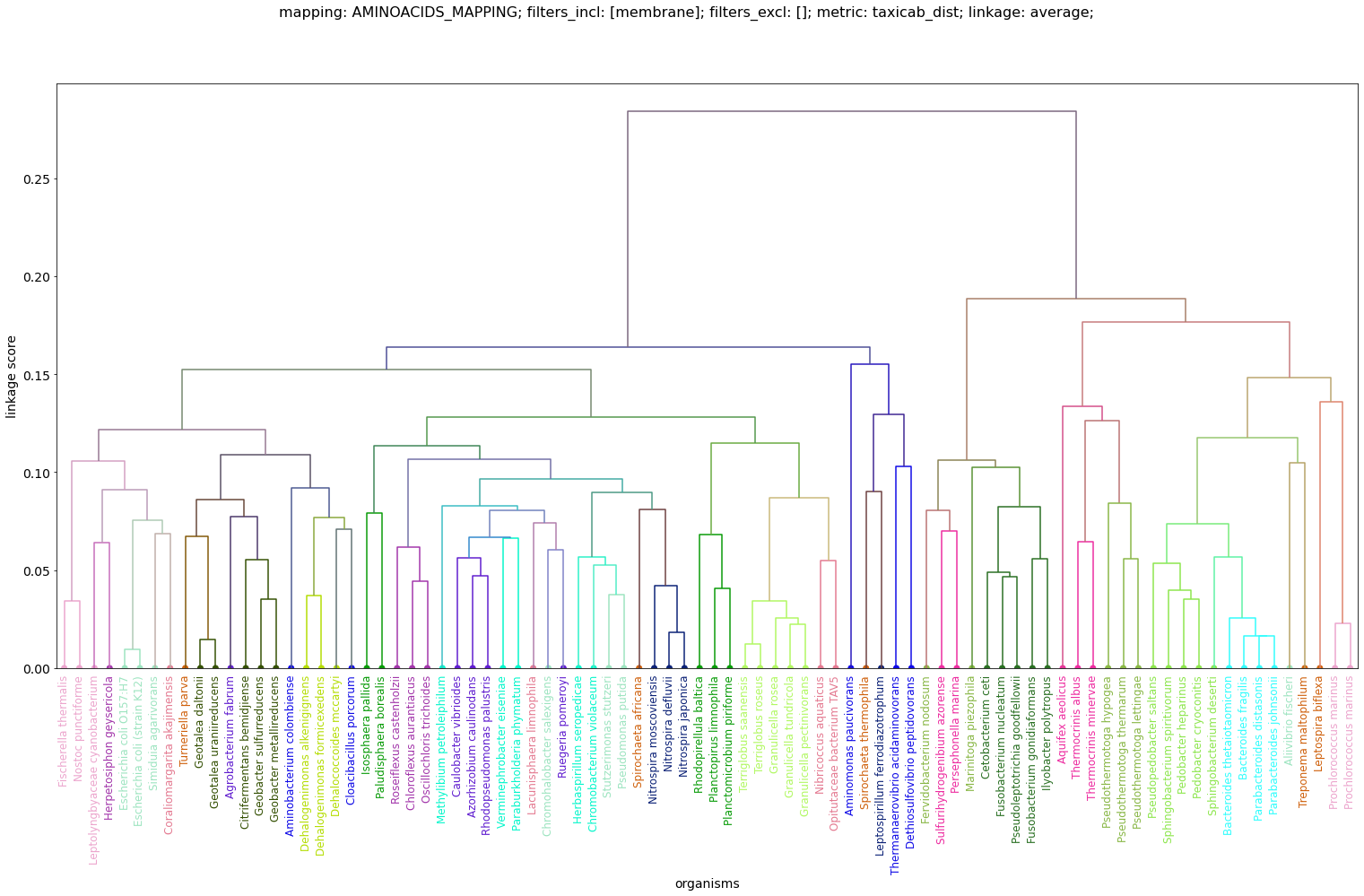

Supplement: Supplementary file 1 [file ijms-27-00109-s001.zip › kmers_supplementary/dendrograms/k1/AMINOACIDS_MAPPING/membrane_/taxicab_dist_average.png]

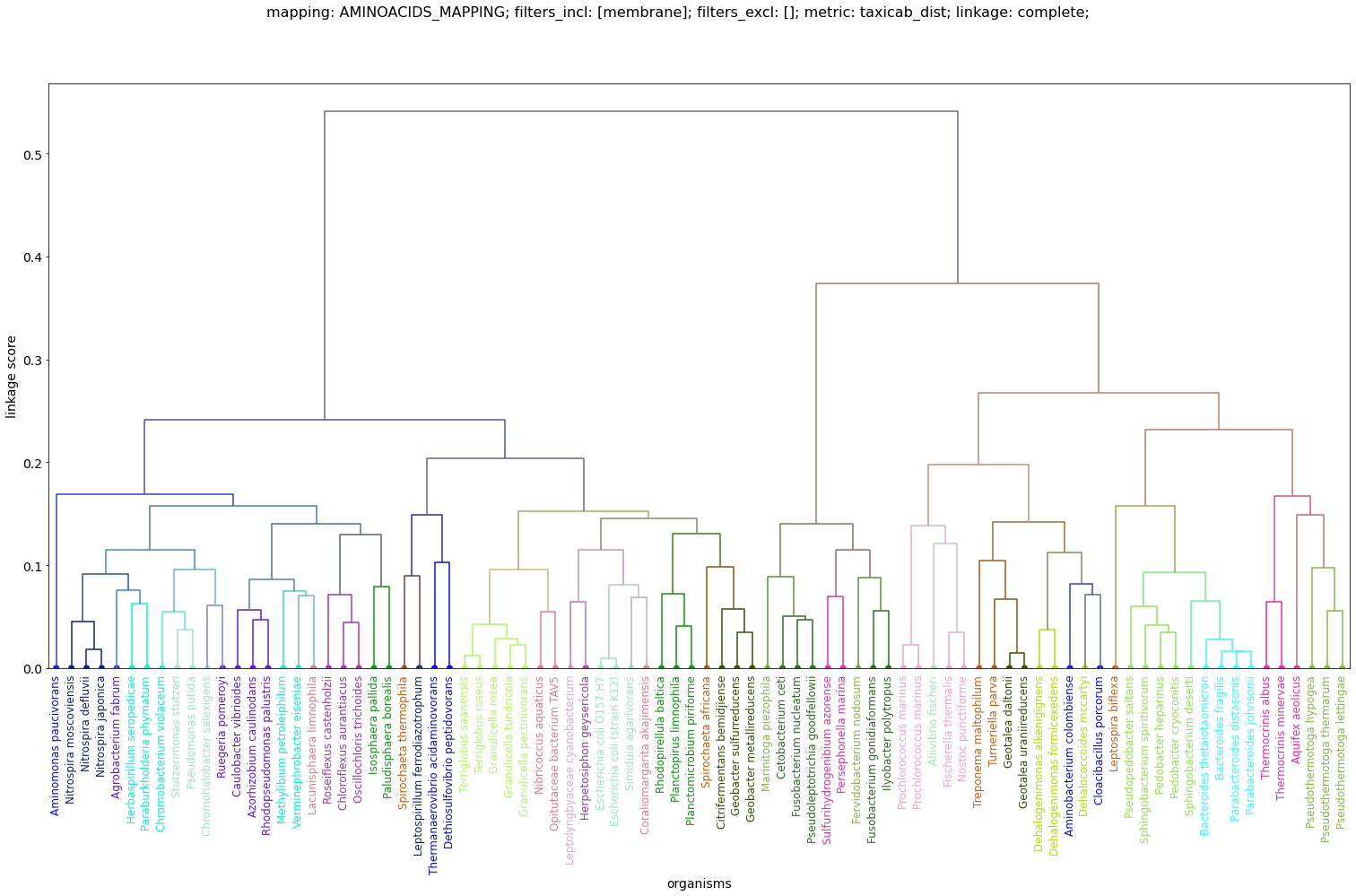

Supplement: Supplementary file 1 [file ijms-27-00109-s001.zip › kmers_supplementary/dendrograms/k1/AMINOACIDS_MAPPING/membrane_/taxicab_dist_complete.png]

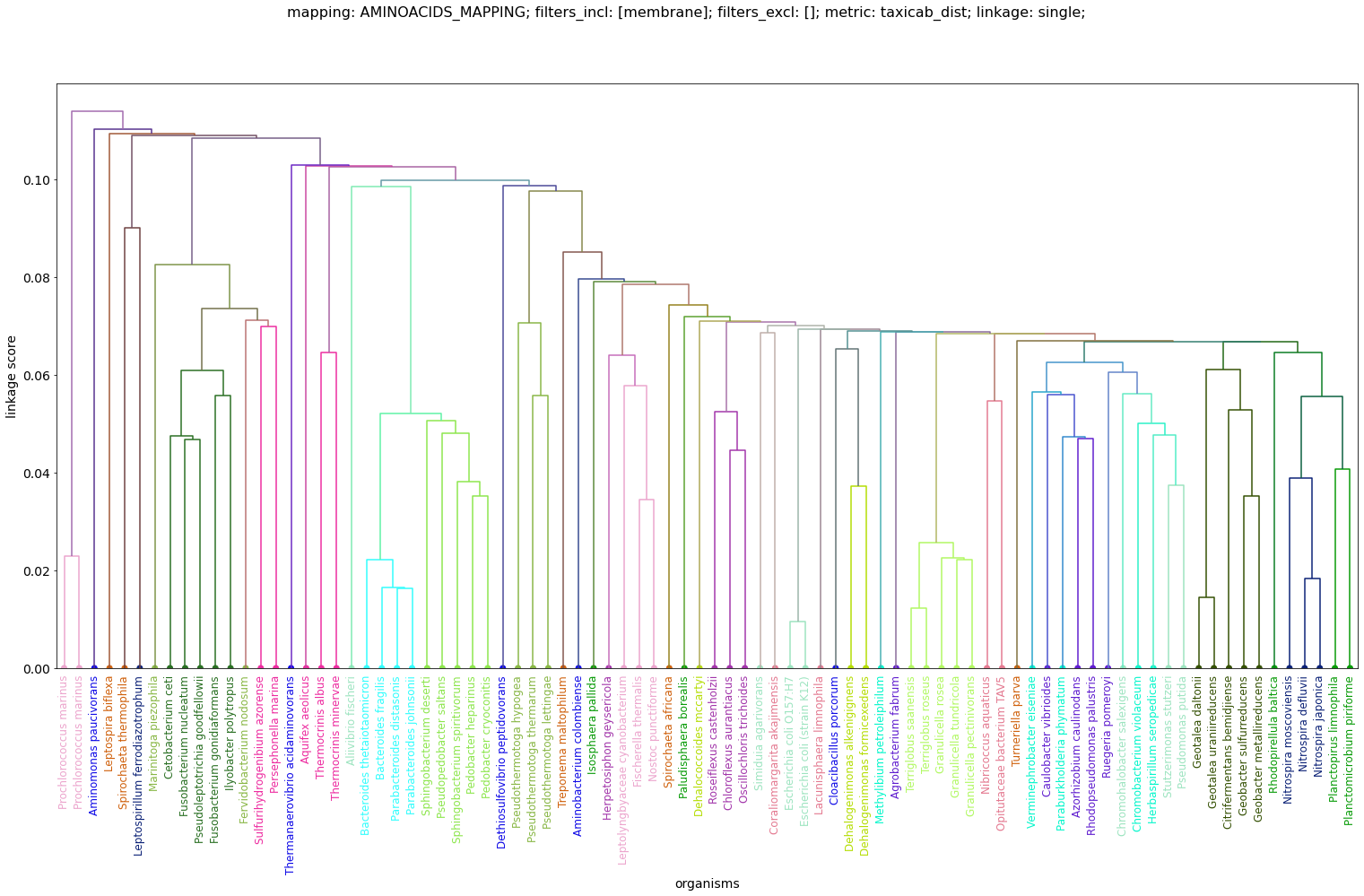

Supplement: Supplementary file 1 [file ijms-27-00109-s001.zip › kmers_supplementary/dendrograms/k1/AMINOACIDS_MAPPING/membrane_/taxicab_dist_single.png]

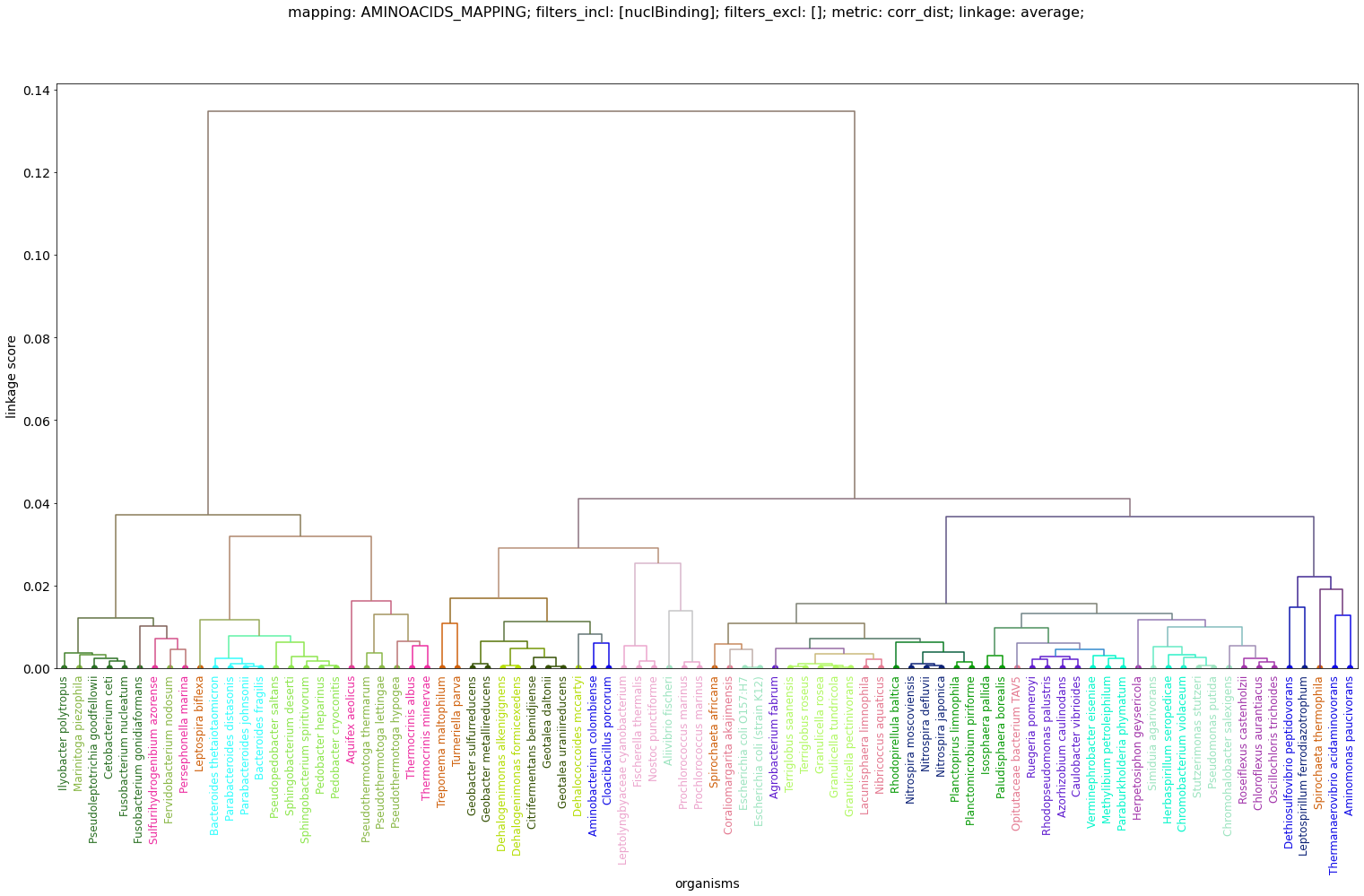

Supplement: Supplementary file 1 [file ijms-27-00109-s001.zip › kmers_supplementary/dendrograms/k1/AMINOACIDS_MAPPING/nuclBinding_/corr_dist_average.png]

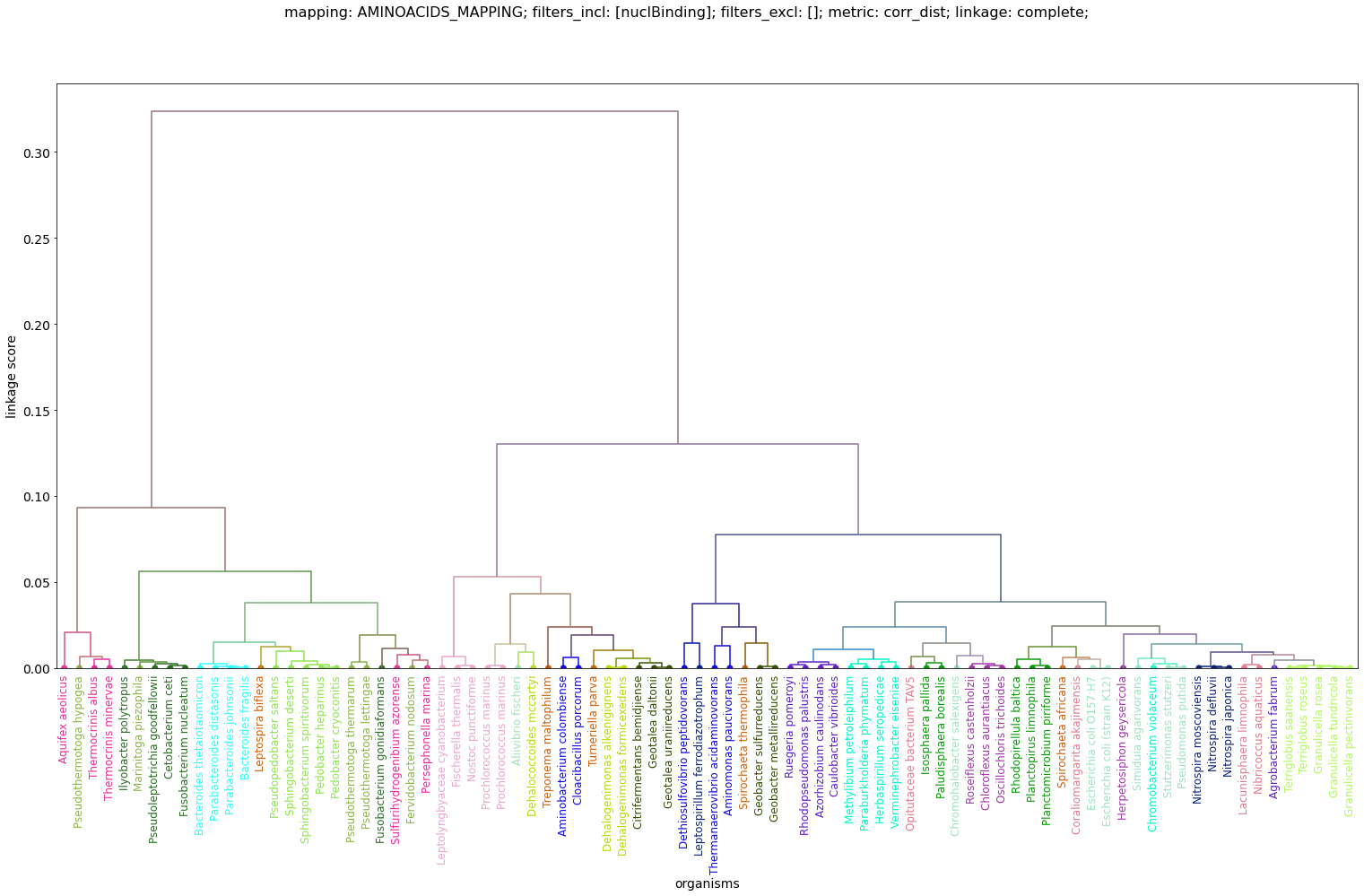

Supplement: Supplementary file 1 [file ijms-27-00109-s001.zip › kmers_supplementary/dendrograms/k1/AMINOACIDS_MAPPING/nuclBinding_/corr_dist_complete.png]

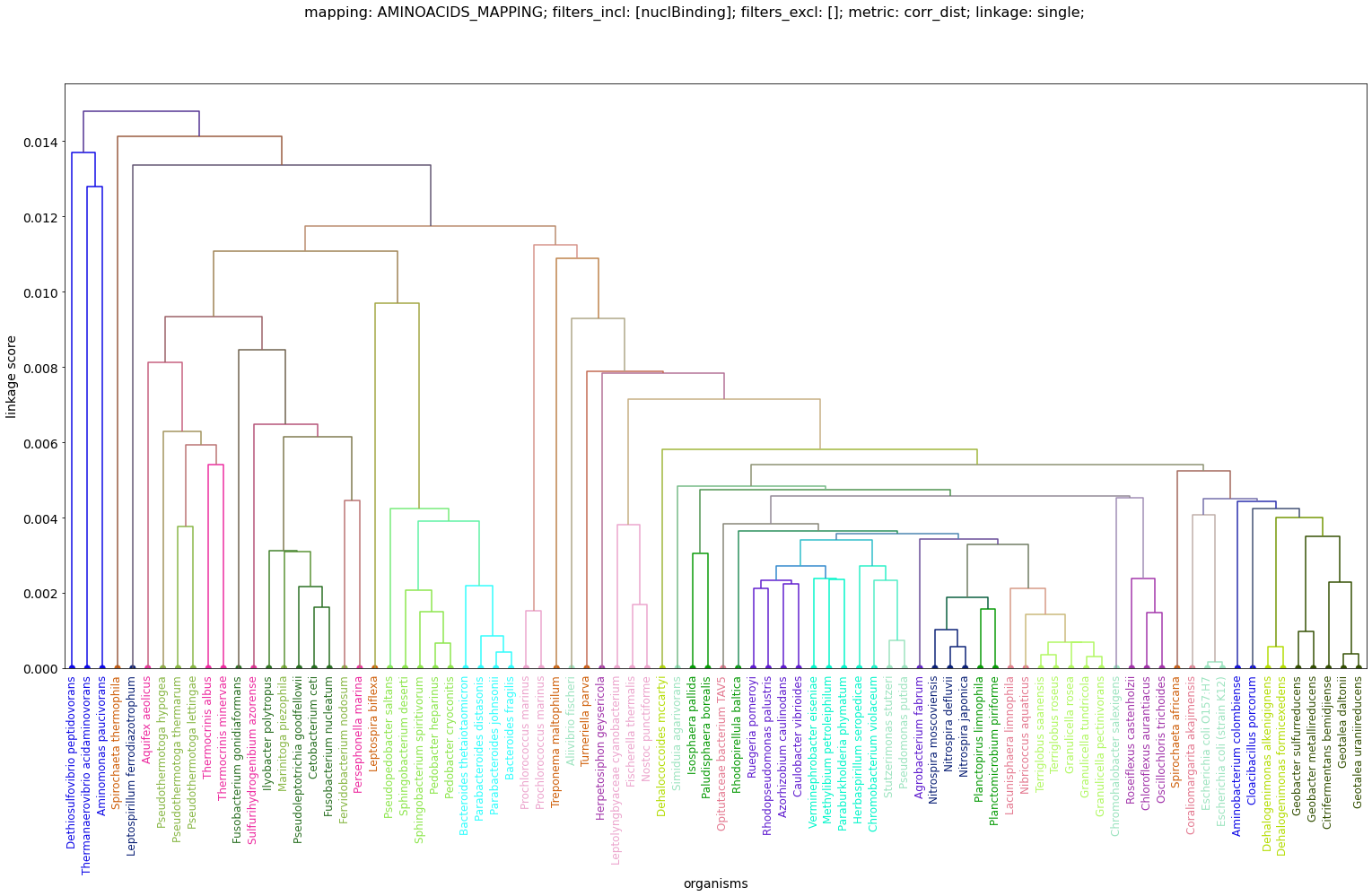

Supplement: Supplementary file 1 [file ijms-27-00109-s001.zip › kmers_supplementary/dendrograms/k1/AMINOACIDS_MAPPING/nuclBinding_/corr_dist_single.png]

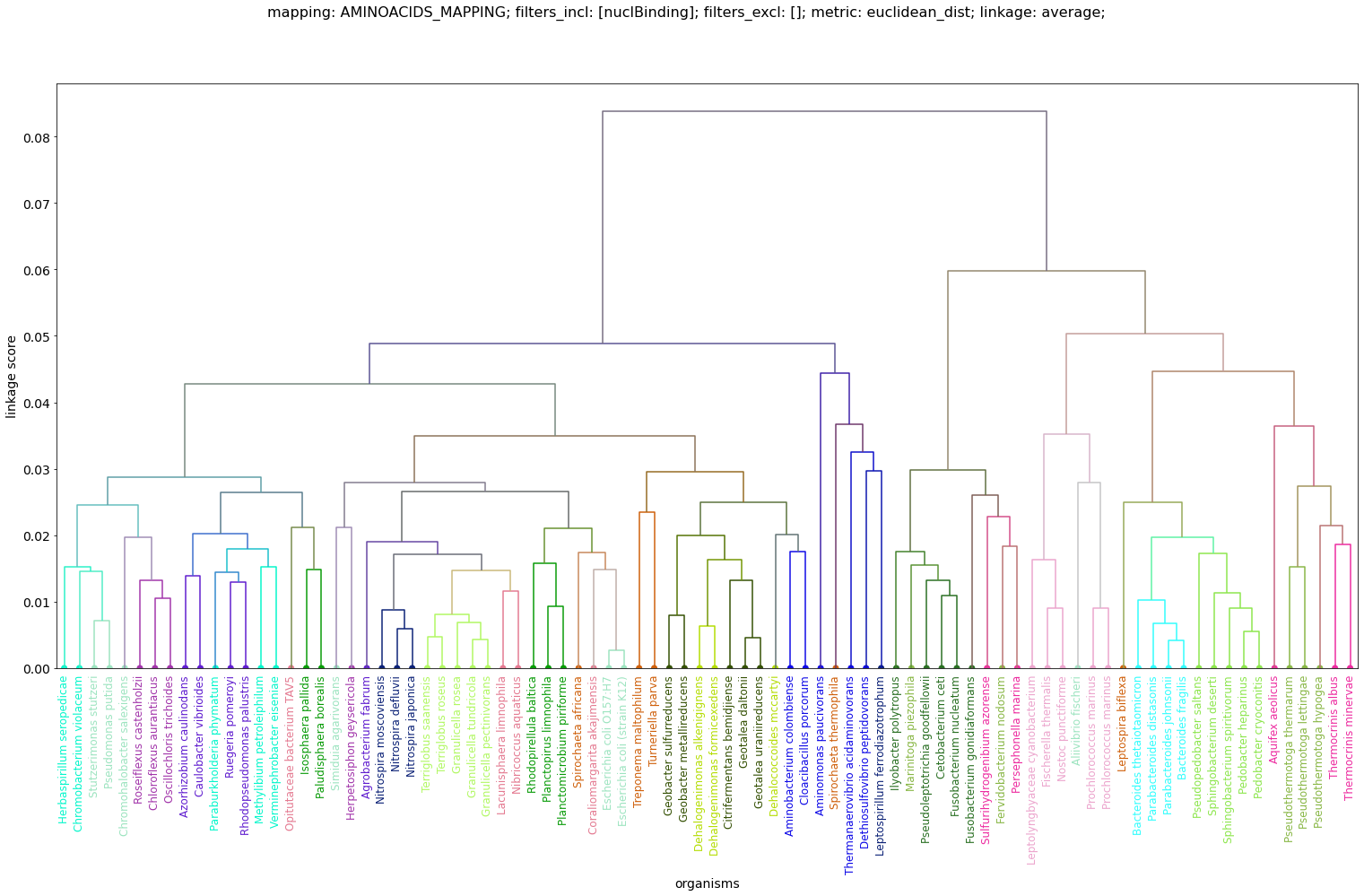

Supplement: Supplementary file 1 [file ijms-27-00109-s001.zip › kmers_supplementary/dendrograms/k1/AMINOACIDS_MAPPING/nuclBinding_/euclidean_dist_average.png]

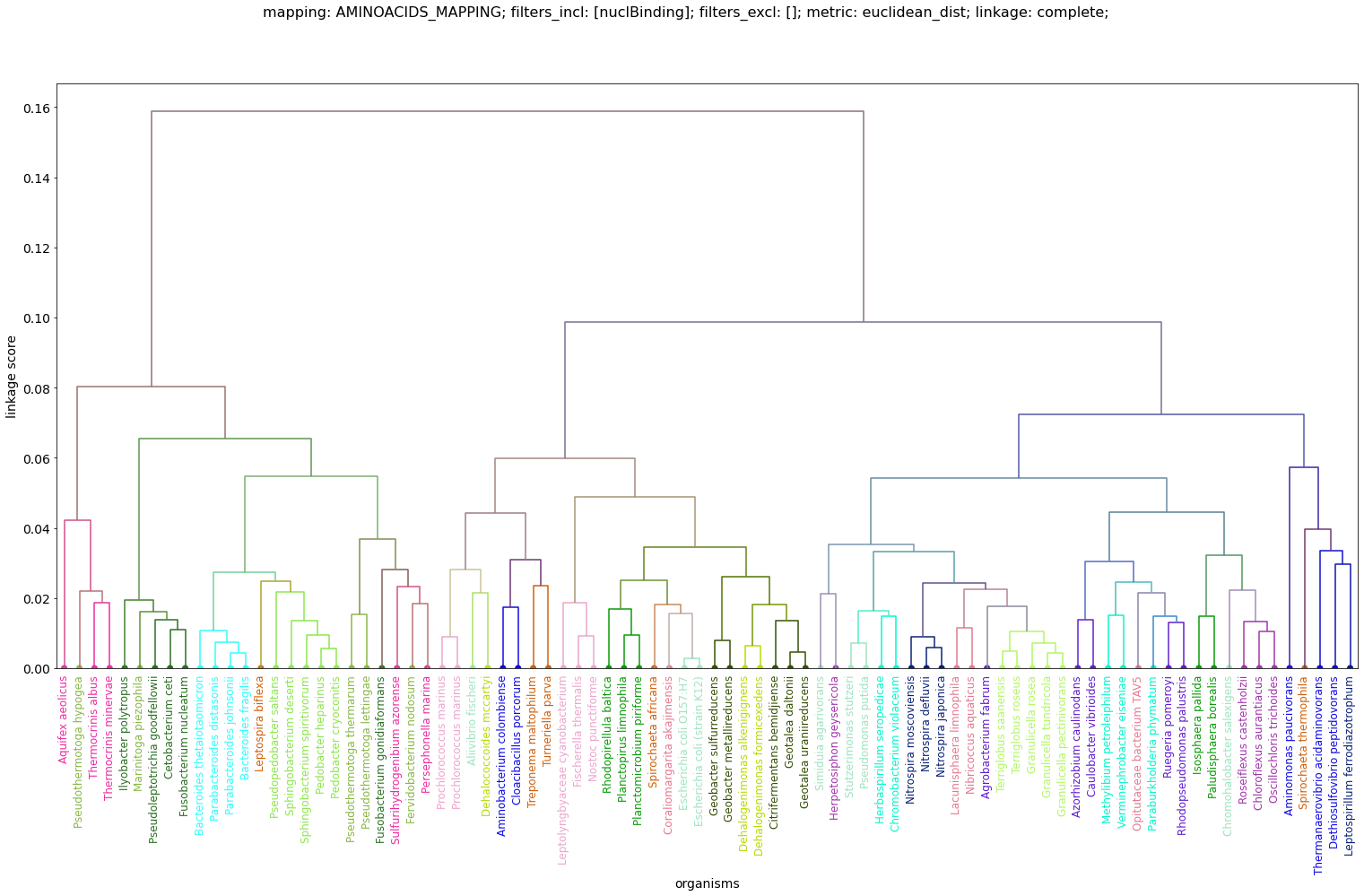

Supplement: Supplementary file 1 [file ijms-27-00109-s001.zip › kmers_supplementary/dendrograms/k1/AMINOACIDS_MAPPING/nuclBinding_/euclidean_dist_complete.png]

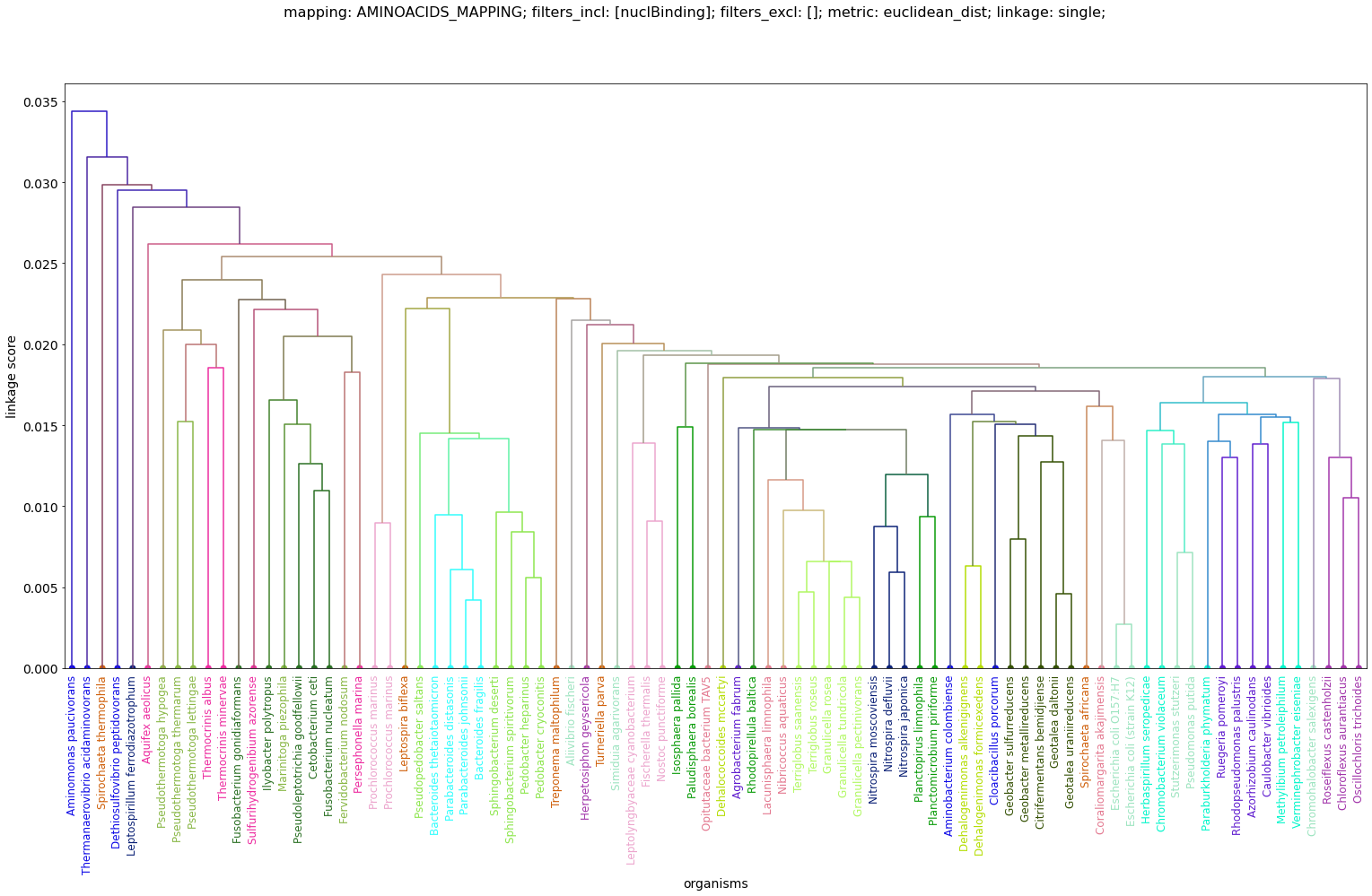

Supplement: Supplementary file 1 [file ijms-27-00109-s001.zip › kmers_supplementary/dendrograms/k1/AMINOACIDS_MAPPING/nuclBinding_/euclidean_dist_single.png]

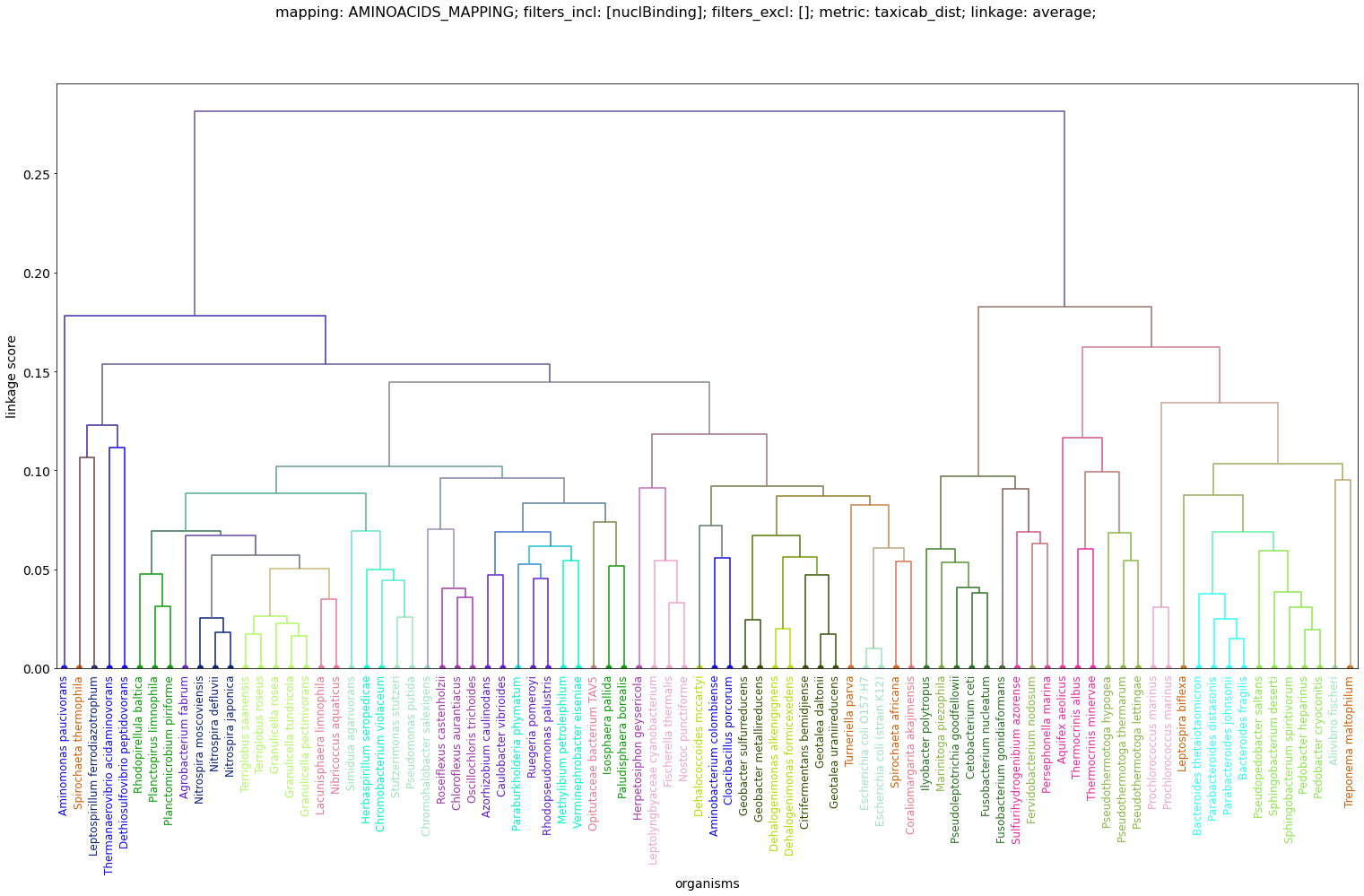

Supplement: Supplementary file 1 [file ijms-27-00109-s001.zip › kmers_supplementary/dendrograms/k1/AMINOACIDS_MAPPING/nuclBinding_/taxicab_dist_average.png]

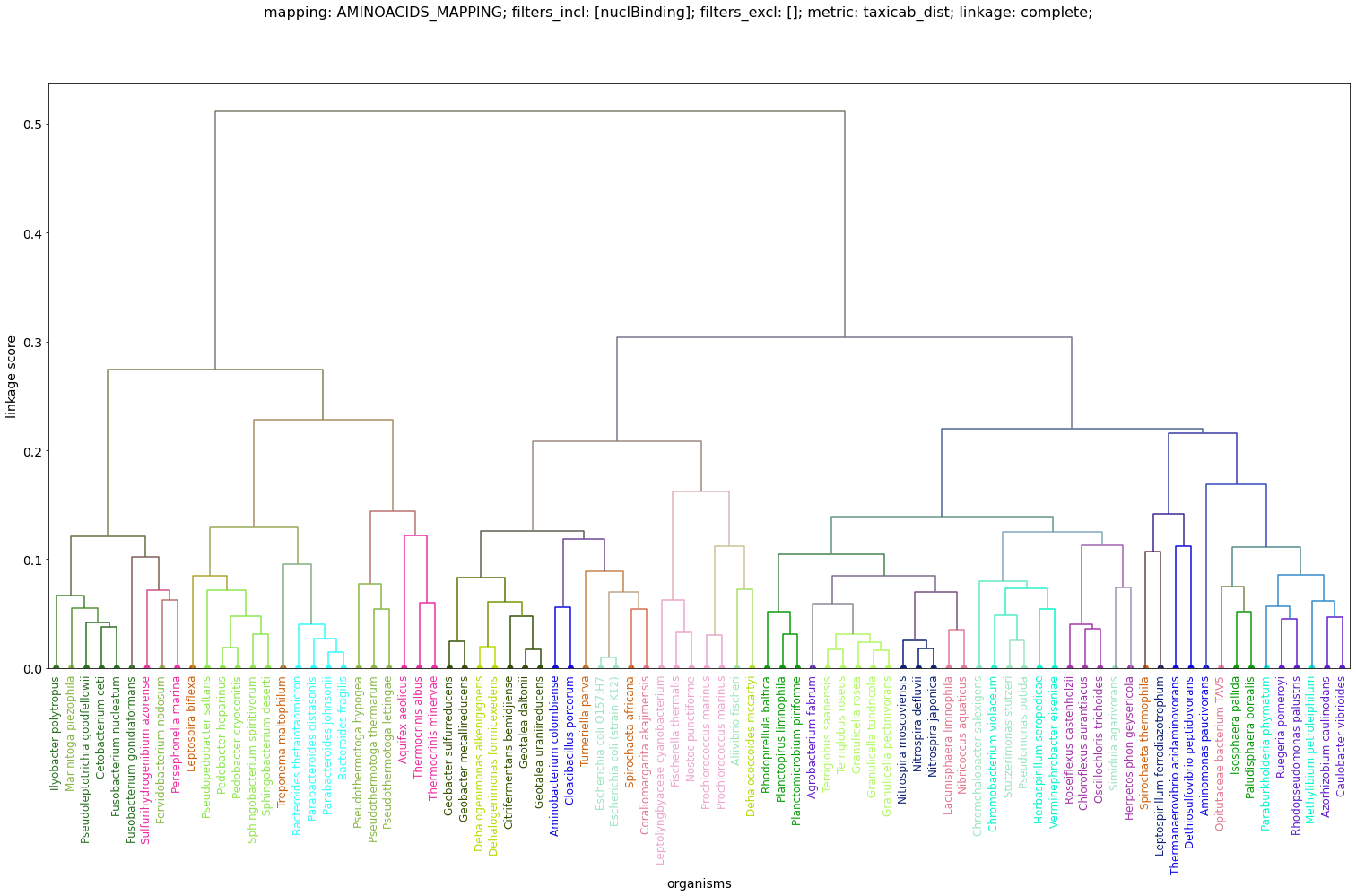

Supplement: Supplementary file 1 [file ijms-27-00109-s001.zip › kmers_supplementary/dendrograms/k1/AMINOACIDS_MAPPING/nuclBinding_/taxicab_dist_complete.png]

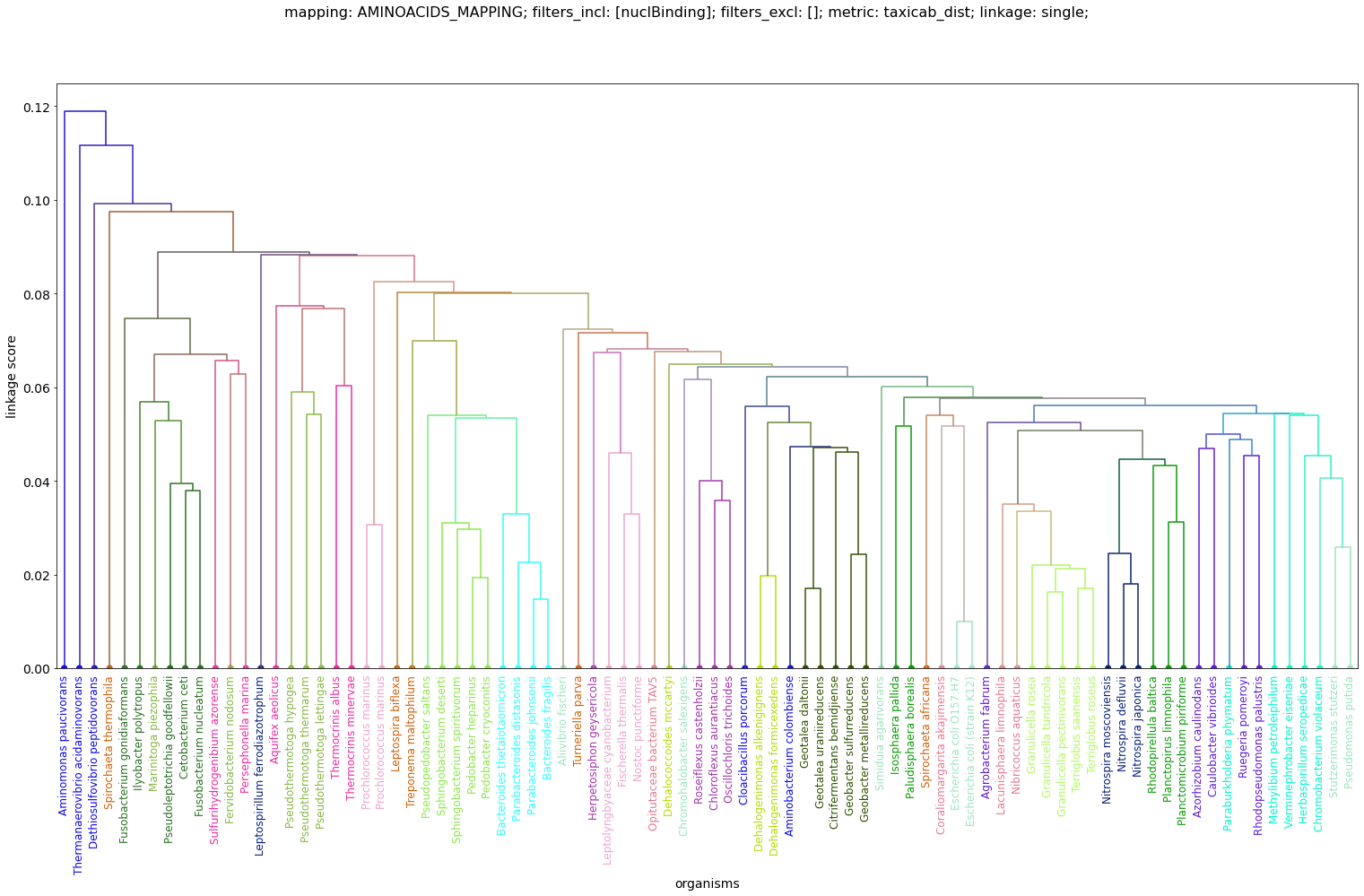

Supplement: Supplementary file 1 [file ijms-27-00109-s001.zip › kmers_supplementary/dendrograms/k1/AMINOACIDS_MAPPING/nuclBinding_/taxicab_dist_single.png]

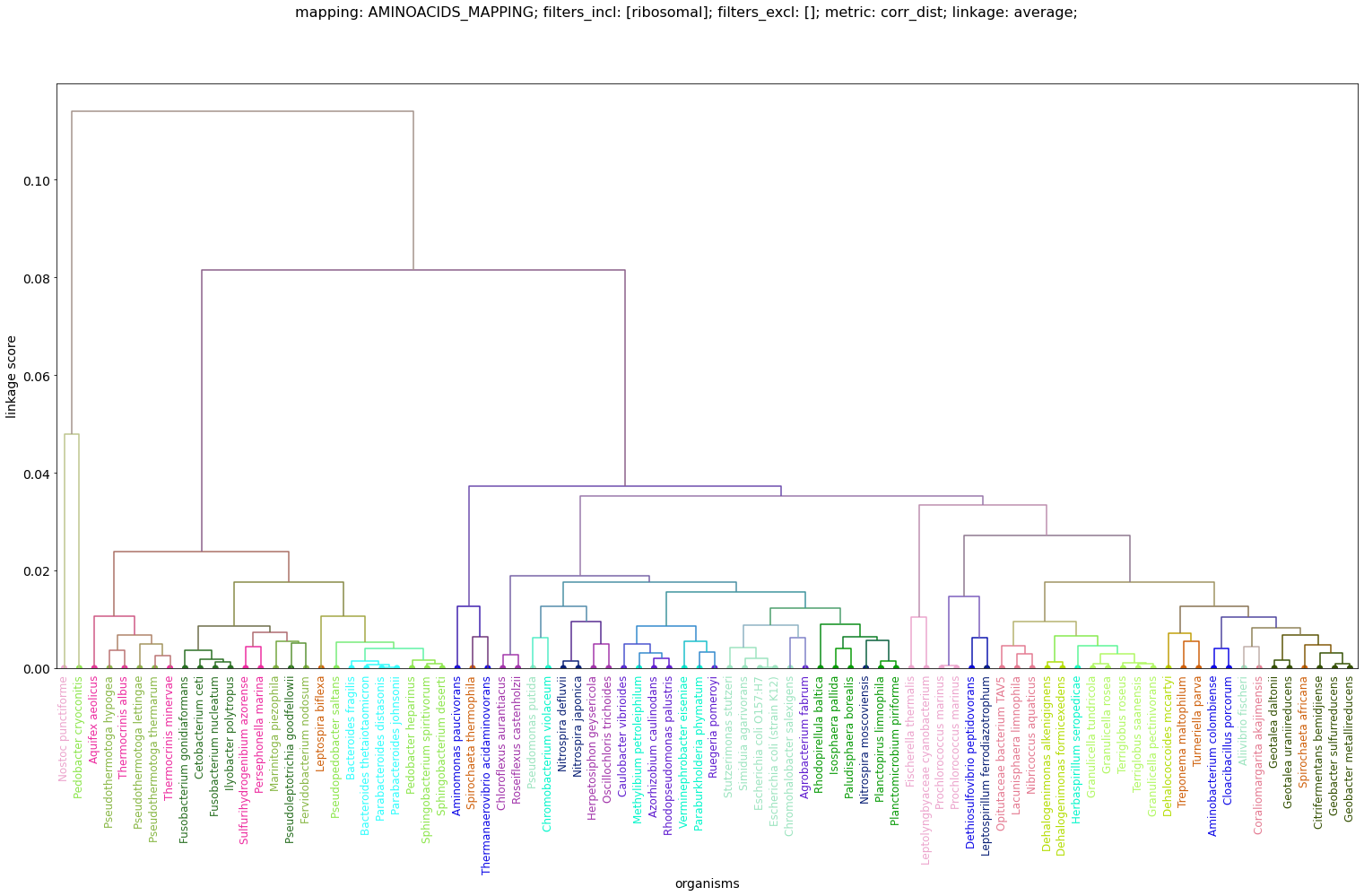

Supplement: Supplementary file 1 [file ijms-27-00109-s001.zip › kmers_supplementary/dendrograms/k1/AMINOACIDS_MAPPING/ribosomal_/corr_dist_average.png]

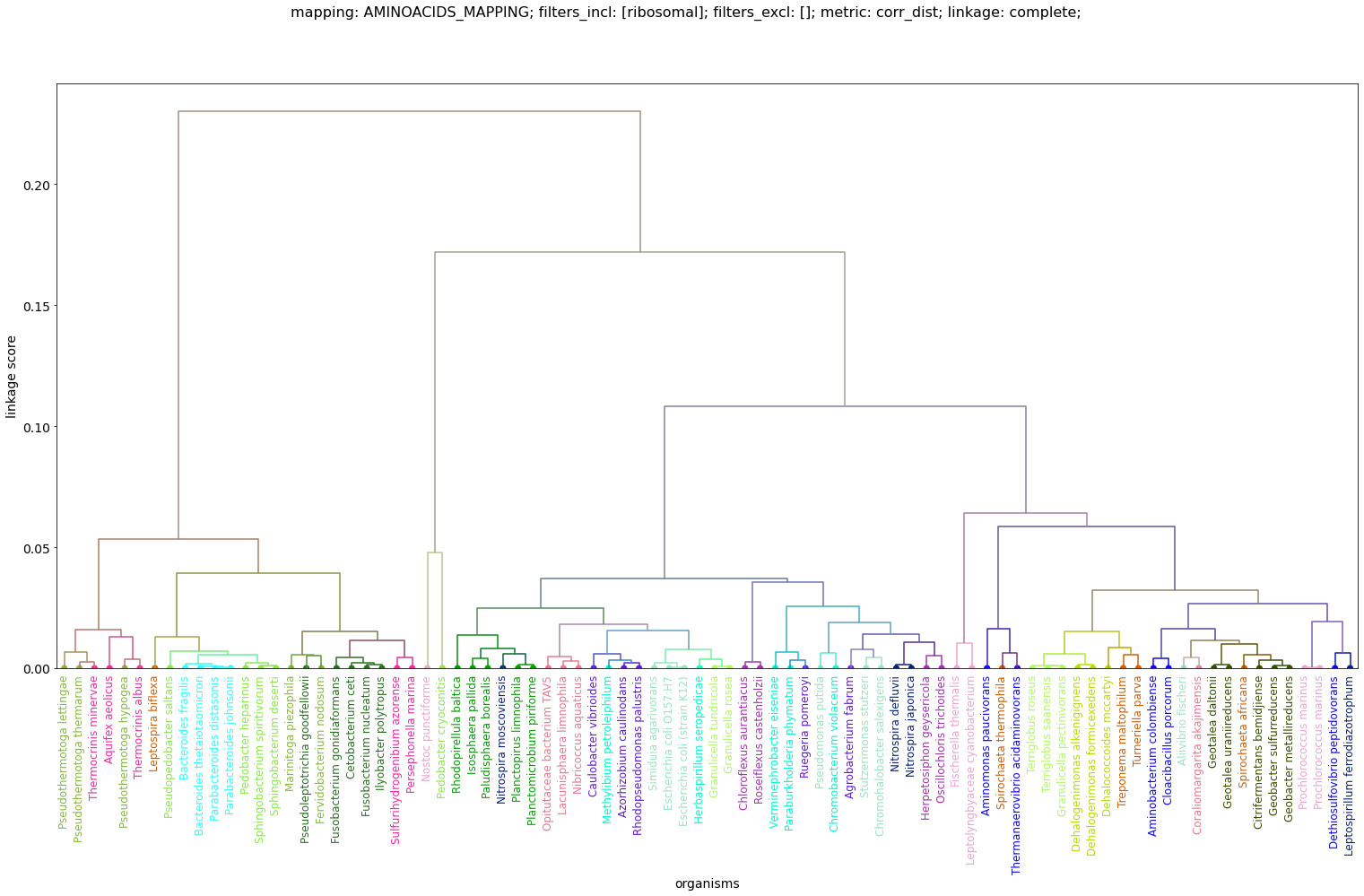

Supplement: Supplementary file 1 [file ijms-27-00109-s001.zip › kmers_supplementary/dendrograms/k1/AMINOACIDS_MAPPING/ribosomal_/corr_dist_complete.png]

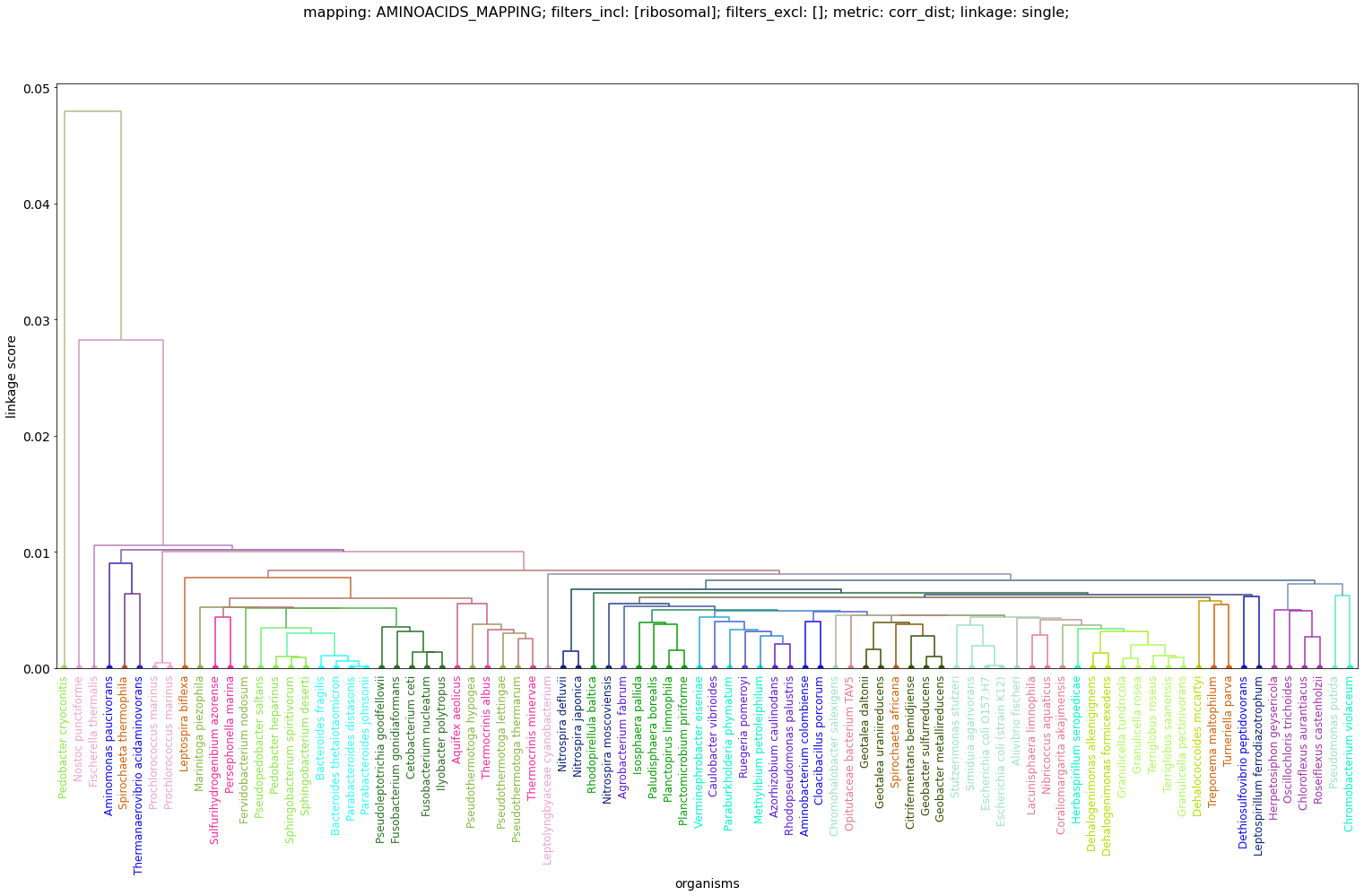

Supplement: Supplementary file 1 [file ijms-27-00109-s001.zip › kmers_supplementary/dendrograms/k1/AMINOACIDS_MAPPING/ribosomal_/corr_dist_single.png]

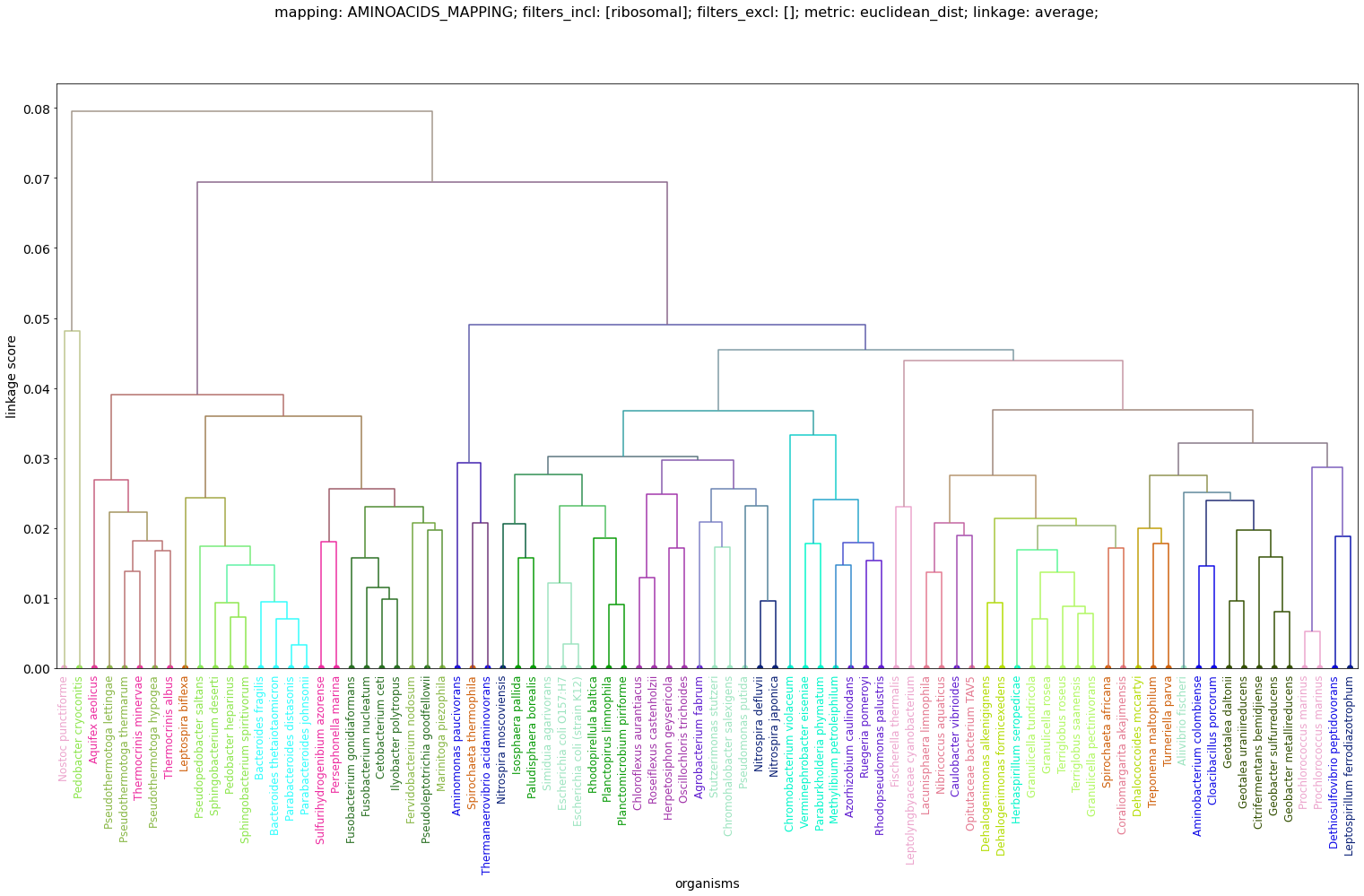

Supplement: Supplementary file 1 [file ijms-27-00109-s001.zip › kmers_supplementary/dendrograms/k1/AMINOACIDS_MAPPING/ribosomal_/euclidean_dist_average.png]

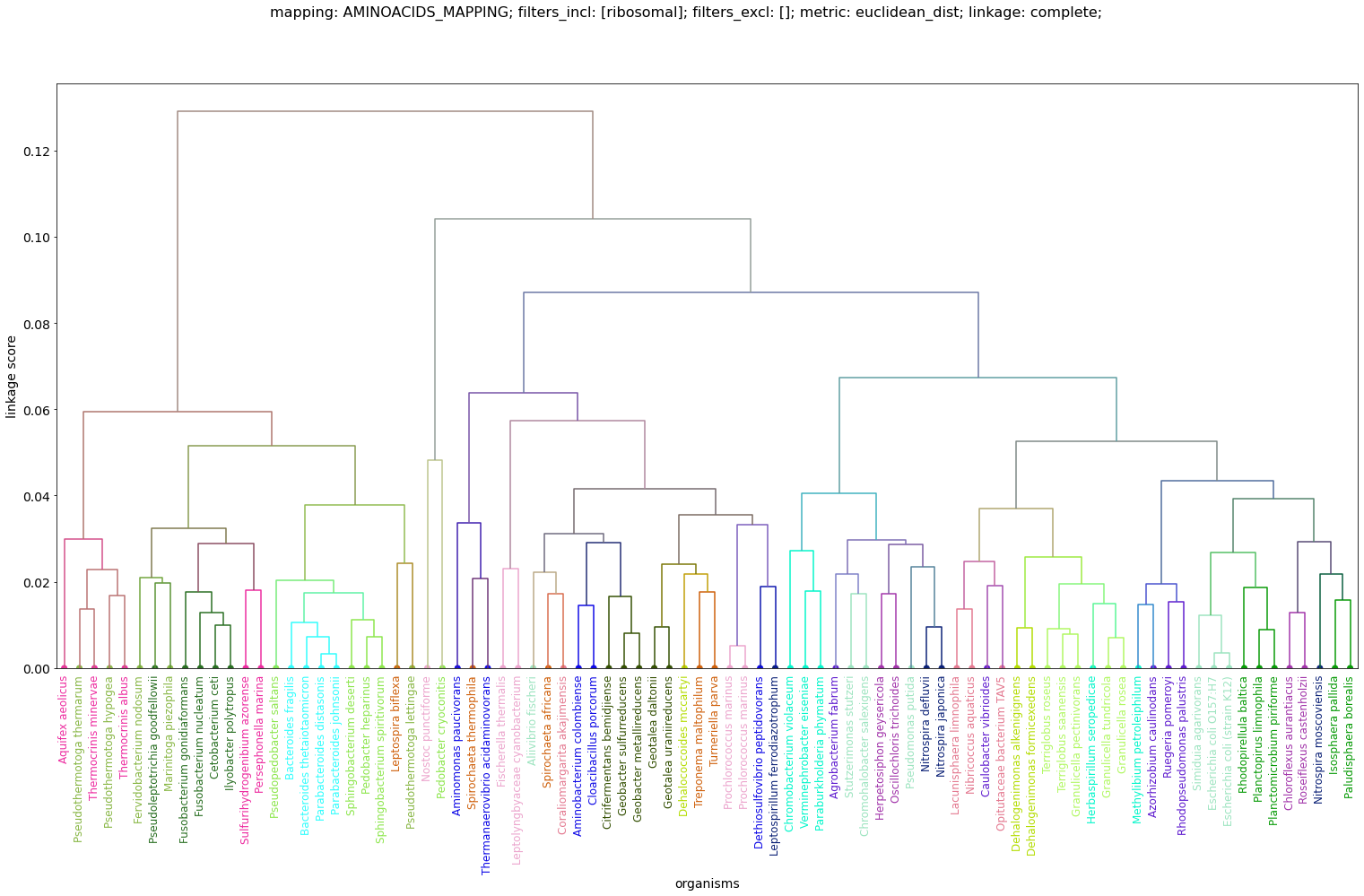

Supplement: Supplementary file 1 [file ijms-27-00109-s001.zip › kmers_supplementary/dendrograms/k1/AMINOACIDS_MAPPING/ribosomal_/euclidean_dist_complete.png]

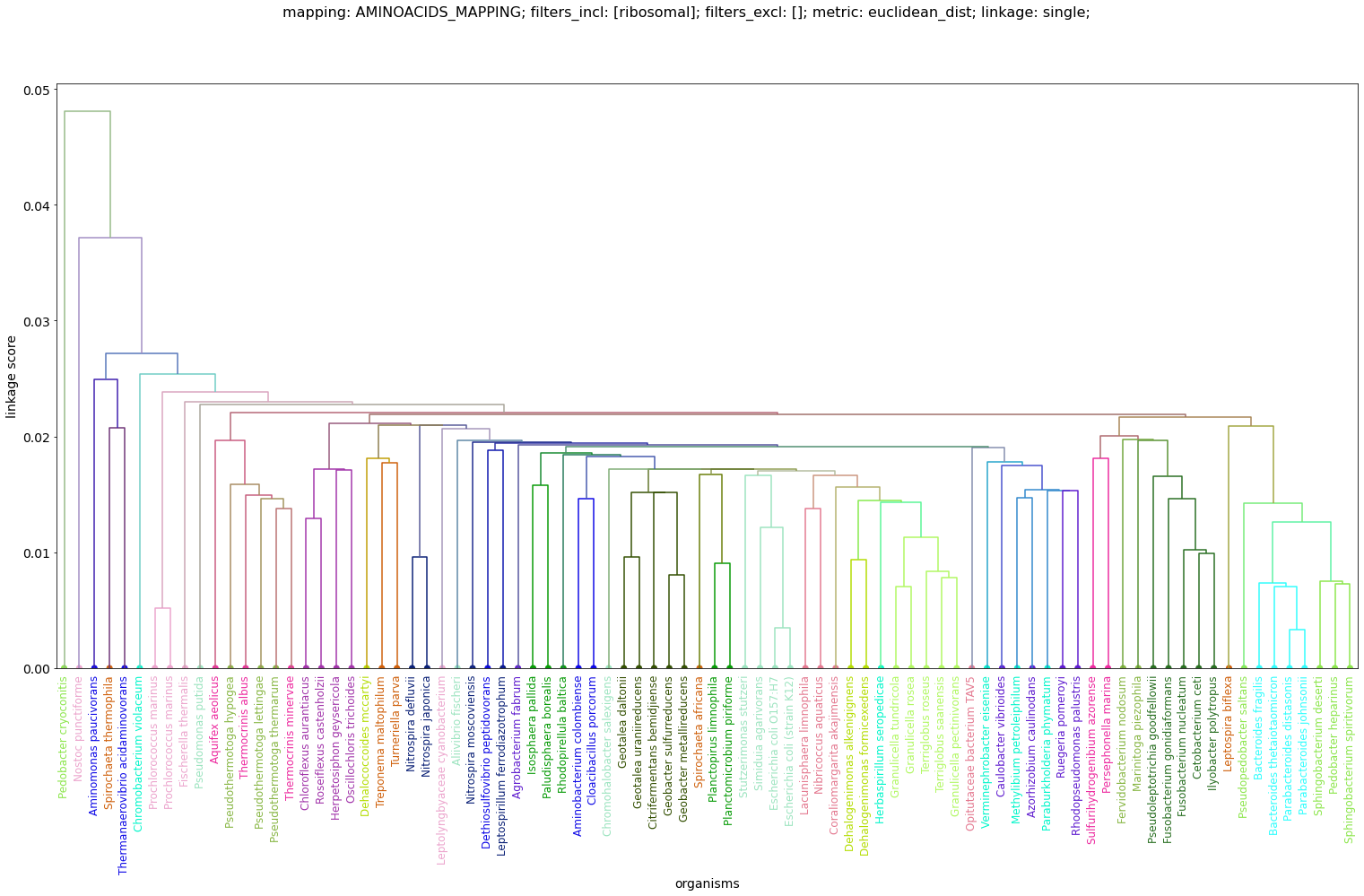

Supplement: Supplementary file 1 [file ijms-27-00109-s001.zip › kmers_supplementary/dendrograms/k1/AMINOACIDS_MAPPING/ribosomal_/euclidean_dist_single.png]

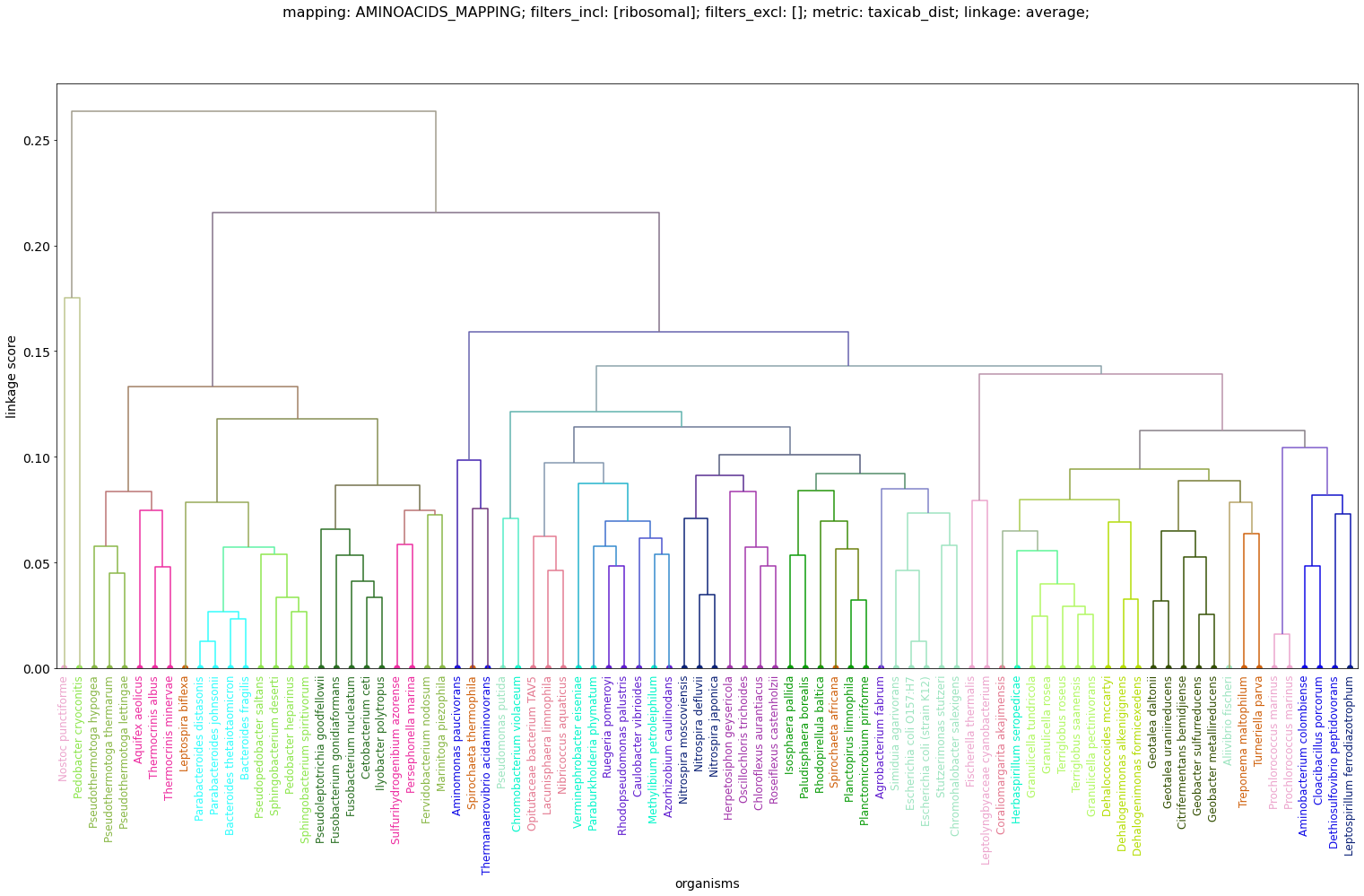

Supplement: Supplementary file 1 [file ijms-27-00109-s001.zip › kmers_supplementary/dendrograms/k1/AMINOACIDS_MAPPING/ribosomal_/taxicab_dist_average.png]

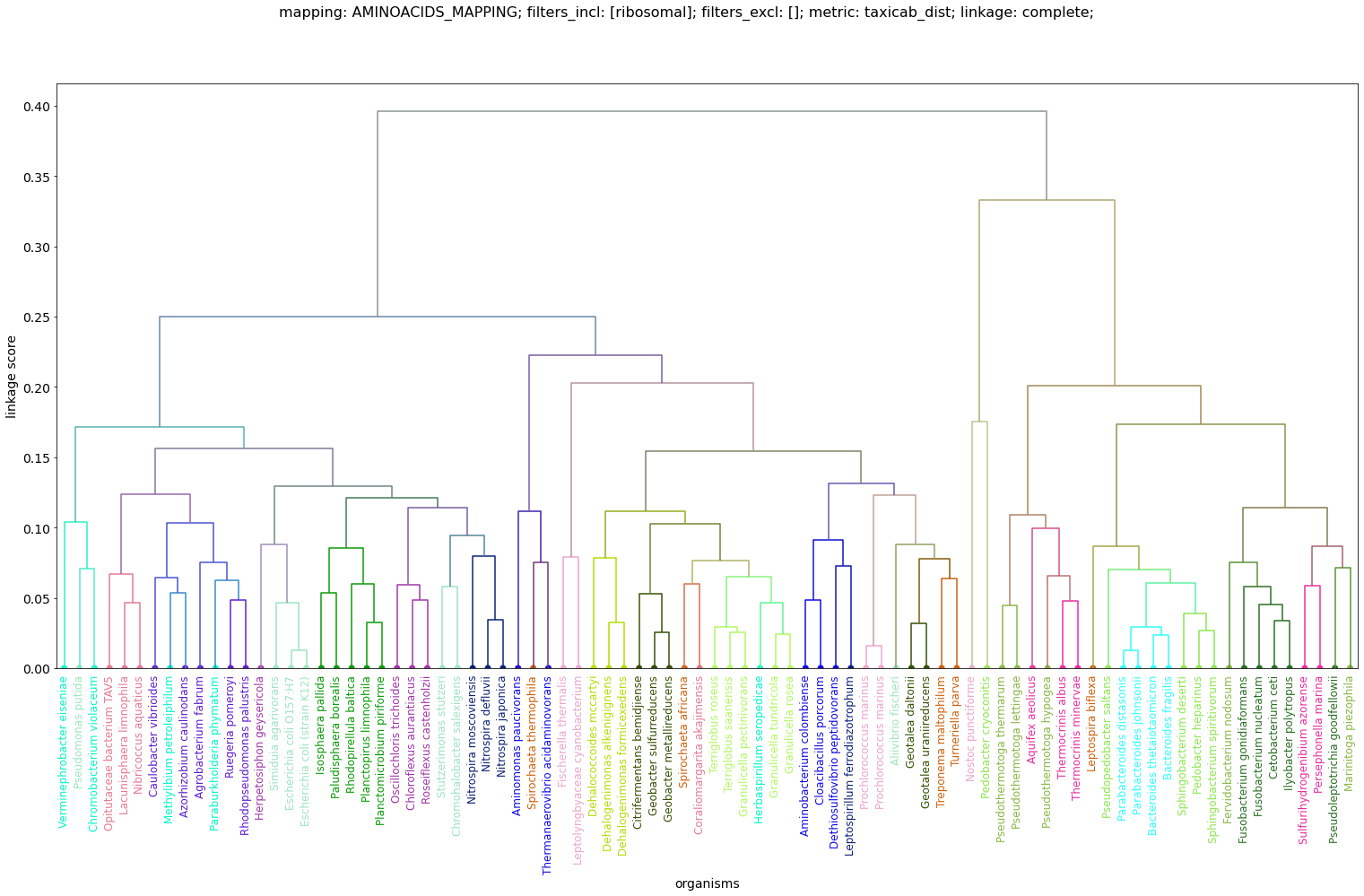

Supplement: Supplementary file 1 [file ijms-27-00109-s001.zip › kmers_supplementary/dendrograms/k1/AMINOACIDS_MAPPING/ribosomal_/taxicab_dist_complete.png]

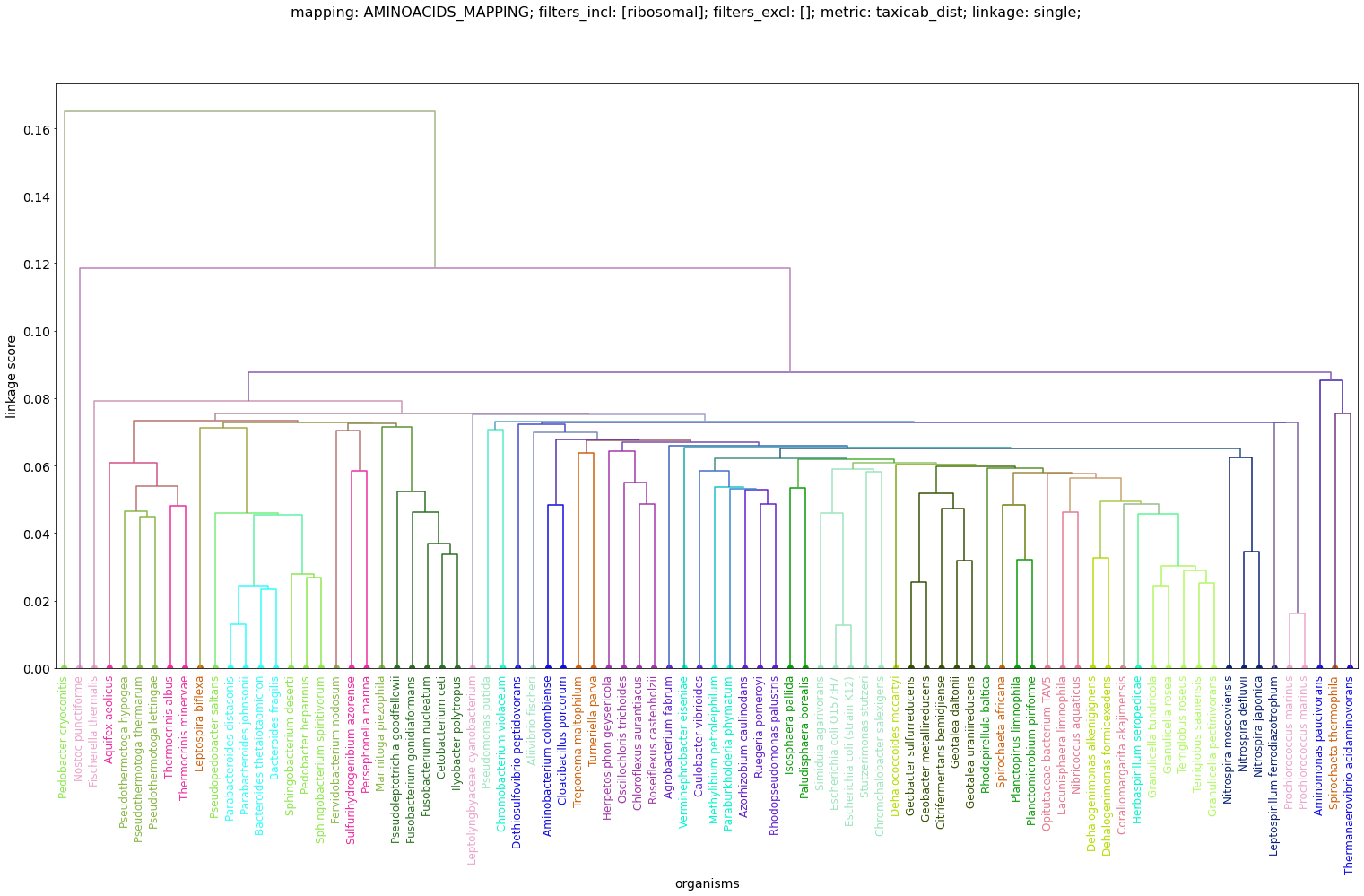

Supplement: Supplementary file 1 [file ijms-27-00109-s001.zip › kmers_supplementary/dendrograms/k1/AMINOACIDS_MAPPING/ribosomal_/taxicab_dist_single.png]

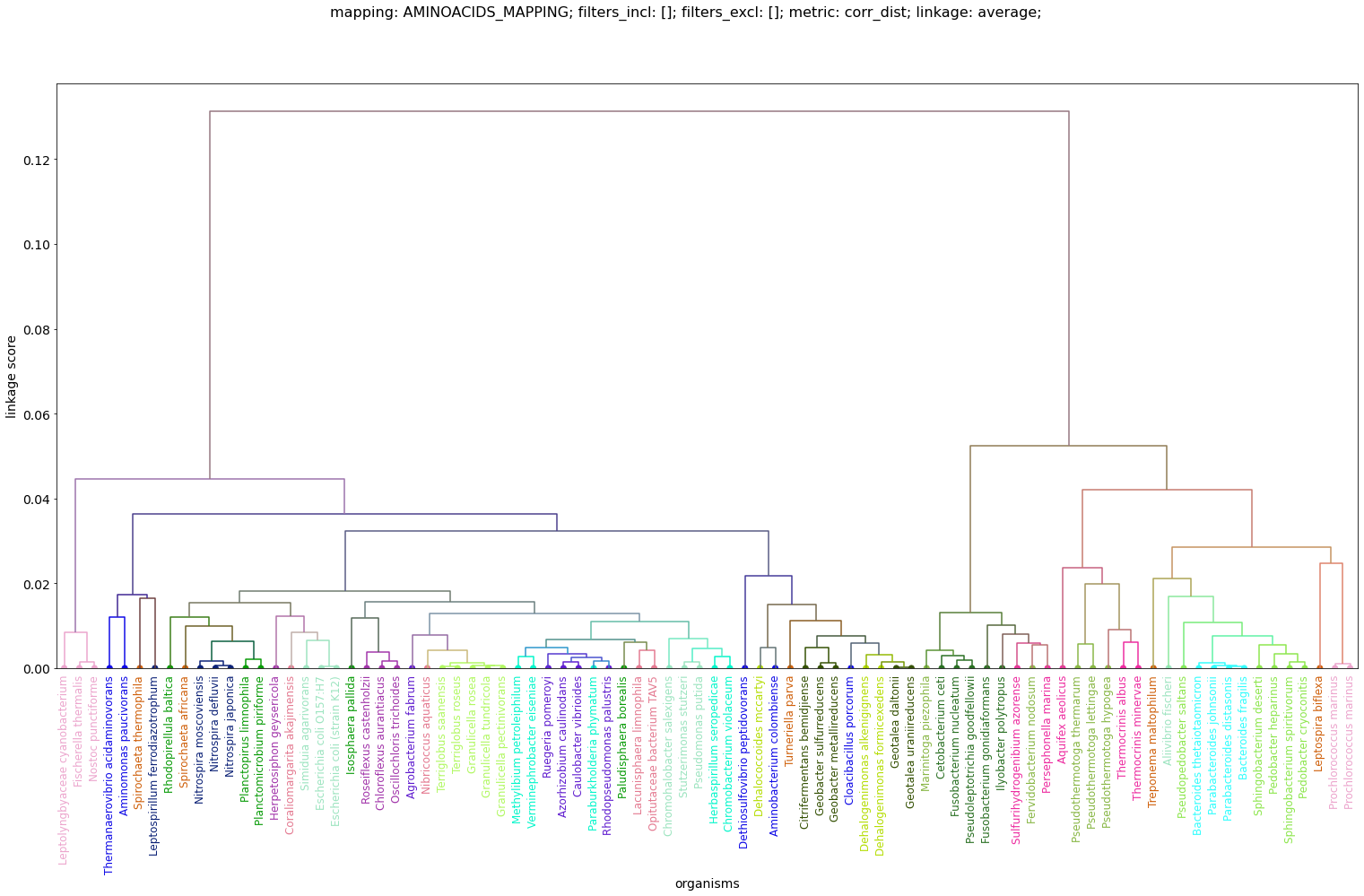

Supplement: Supplementary file 1 [file ijms-27-00109-s001.zip › kmers_supplementary/dendrograms/k1/AMINOACIDS_MAPPING/_/corr_dist_average.png]
